# New Oxazolo[5,4-*d*]pyrimidines as Potential Anticancer Agents: Their Design, Synthesis, and In Vitro Biological Activity Research

**DOI:** 10.3390/ijms231911694

**Published:** 2022-10-02

**Authors:** Aleksandra Sochacka-Ćwikła, Marcin Mączyński, Żaneta Czyżnikowska, Benita Wiatrak, Izabela Jęśkowiak, Albert Czerski, Andrzej Regiec

**Affiliations:** 1 Department of Organic Chemistry and Drug Technology, Faculty of Pharmacy, Wroclaw Medical University, 211A Borowska Street, 50-556 Wrocław, Poland; 2 Department of Inorganic Chemistry, Faculty of Pharmacy, Wroclaw Medical University, 211A Borowska Street, 50-556 Wrocław, Poland; 3 Department of Pharmacology, Faculty of Medicine, Wroclaw Medical University, 2 Mikulicza-Radeckiego Street, 50-345 Wrocław, Poland; 4 Department of Biostructure and Animal Physiology, Wroclaw University of Environmental and Life Sciences, 25/27 Norwida Street, 50-375 Wrocław, Poland

**Keywords:** oxazolo[5,4-*d*]pyrimidines, isoxazole, molecular docking, anticancer activity, cytotoxicity, antimetabolites, VEGFR-2 inhibitors

## Abstract

Cancer is a large group of diseases in which the rapid proliferation of abnormal cells generally leads to metastasis to surrounding tissues or more distant ones through the lymphatic and blood vessels, making it the second leading cause of death worldwide. The main challenge in designing a modern anticancer therapy is to develop selective compounds that exploit specific molecular targets. In this work, novel oxazolo[5,4-*d*]pyrimidine derivatives were designed, synthesized, and evaluated in vitro for their cytotoxic activity against a panel of four human cancer cell lines (lung carcinoma: A549, breast adenocarcinoma: MCF7, metastatic colon adenocarcinoma: LoVo, primary colon adenocarcinoma: HT29), along with their P-glycoprotein-inhibitory ability and pro-apoptotic activity. These oxazolo[5,4-*d*]pyrimidine derivatives, which are structurally similar to nucleic purine bases in general, are characterized by the presence of a pharmacologically favorable isoxazole substituent at position 2 and aliphatic amino chains at position 7 of the condensed heterocyclic system. In silico analysis of the obtained compounds identified their potent inhibitory activity towards human vascular endothelial growth factor receptor-2 (VEGFR-2). Molecular docking was performed to assess the binding mode of new derivatives to the VEGFR-2 active site. Then, their physicochemical, pharmacokinetic, and pharmacological properties (i.e., ADME—administration, distribution, metabolism, and excretion) were also predicted to assess their druglikeness. In particular, compound **3g** (with a 3-(*N*,*N*-dimethylamino)propyl substituent) was found to be the most potent against the HT29 cell line, with a 50% cytotoxic concentration (CC_50_) of 58.4 µM, exceeding the activity of fluorouracil (CC_50_ = 381.2 μM) and equaling the activity of cisplatin (CC_50_ = 47.2 µM), while being less toxic to healthy human cells (such as normal human dermal fibroblasts (NHDFs)) than these reference drugs. The results suggest that compound **3g** is a potentially promising candidate for the treatment of primary colorectal cancer.

## 1. Introduction

**Figure 1:**
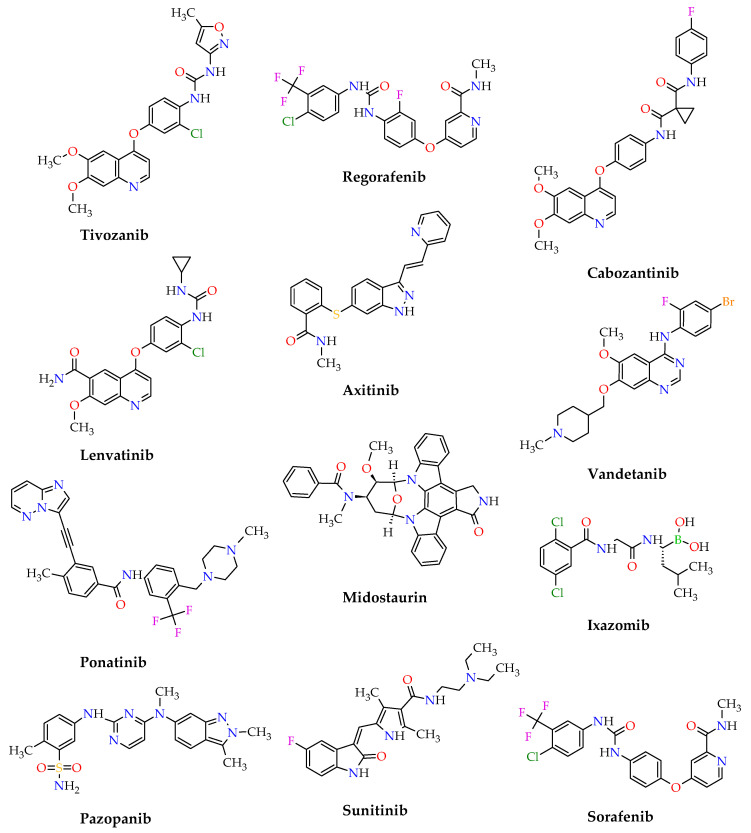
Small-molecule inhibitors with anti-VEGFR activity for the treatment of various types of cancer.

Cancer is a large group of diseases characterized by the abnormal, uncontrollable growth of cells, and can be classified as solid tumors or hematological (blood) cancers. Both types are often diagnosed worldwide and cause a large number of deaths each year. In recent decades, a number of new anticancer drugs with newly understood mechanisms of action have been introduced for treatment. Recently, vascular endothelial growth factor receptor-2 (VEGFR-2) inhibitors have become more widely used in the cancer therapy. For example, the following drugs—which are, among others, VEGFR-2 inhibitors—can be mentioned: tivozanib, regorafenib, cabozantinib, lenvatinib, axitinib, vandetanib, ponatinib, midostaurin, ixazomib, pazopanib, sunitinib, and sorafenib (Figure 1) [1,2,3].

VEGFR-2 is a key factor involved in tumor angiogenesis, since it leads to the activation of downstream signaling, which mediates vascular permeability as well as endothelial cells’ migration, proliferation, and survival. It is also responsible for the reduction in cell apoptosis and changes in cytoskeletal function. Therefore, VEGFR-2 inhibition blocks angiogenesis and, subsequently, reduces tumor growth [4]. The overexpression of VEGFR-2 has been identified in several types of cancer, namely, non-small-cell lung cancer, ovarian cancer, breast cancer, colorectal cancer, hepatocellular carcinoma, and renal carcinoma [5].

**Figure 2:**
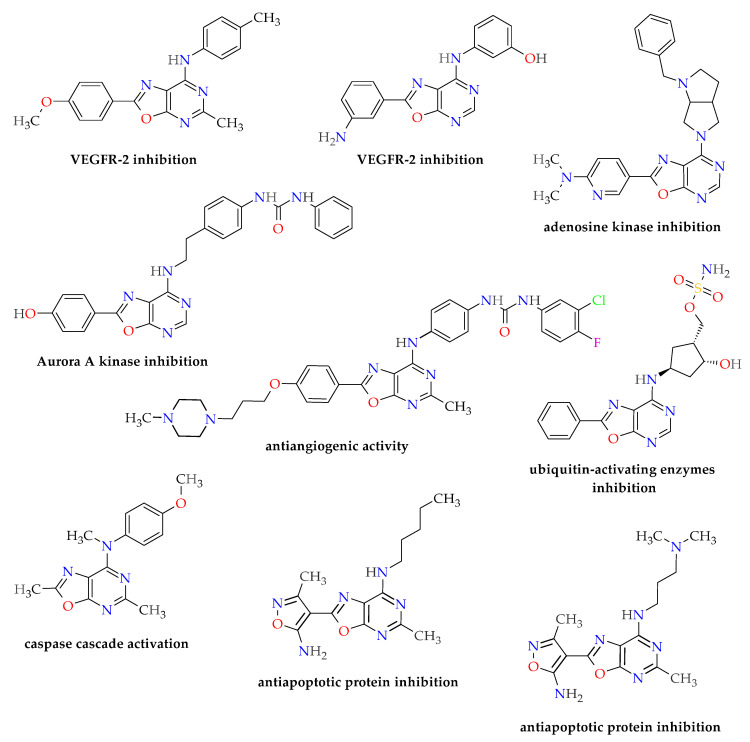
Oxazolo[5,4-*d*]pyrimidines reported as anticancer agents with identified molecular mechanisms of action.

**Figure 3:**
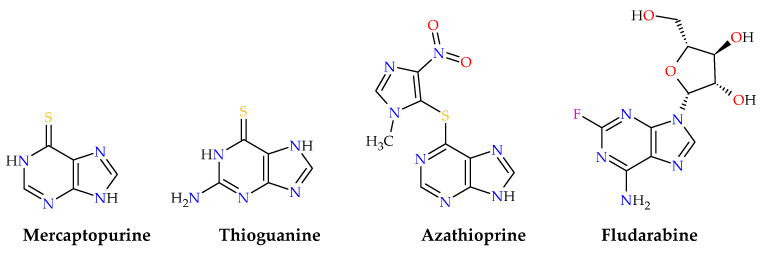
Purine antagonists used as drugs in cancer therapies.

In the biological evaluation of oxazolo[5,4-*d*]pyrimidines as anticancer agents, they were found to be inhibitors of VEGFR-2 [6,7,8], adenosine kinase [9], Aurora A kinase [10], Janus kinase 1 (JAK1) [11], Janus kinase 2 (JAK2) [12] and ubiquitin-activating enzymes (E1 enzymes) [13] (Figure 2). Oxazolo[5,4-*d*]pyrimidines can also activate the caspase cascade [14] or inhibit angiogenesis [15,16], which may result in their inhibition of the growth of certain tumor cell lines. Moreover, some of them show anticancer activity without an identified mode of action [17]. In previous in vitro studies conducted by the authors, oxazolo[5,4-*d*]pyrimidine derivatives have shown anticancer as well as antiviral and immunosuppressive activity through the inhibition of various anti-apoptotic proteins [18]. The oxazolo[5,4-*d*]pyrimidine pharmacophore represents an important heterocyclic moiety in oncology due to its structural similarity to purine bases, in which the imidazole ring is replaced with the oxazole motif. Purine bases such as adenine and guanine are normal components of DNA or coenzymes involved in nucleic acid synthesis. For this reason, oxazolo[5,4-*d*]pyrimidines are potential antimetabolites as purine antagonists similar to known drugs, such as mercaptopurine, thioguanine, azathioprine, or fludarabine (Figure 3). Therefore, they can competitively inhibit the use of the correct substrate (e.g., adenine or guanine) or build in it to form dysfunctional macromolecules.

On the other hand, many published articles have reported that isoxazole-based compounds show anticancer activity via inhibition of heat shock protein 90 (Hsp90) [19,20,21,22], aromatase enzymes [23,24,25], histone deacetylase (HADAC) [26], phospholipase A2 (PLA2) [27], thymidylate synthase (TS) [28,29], or various protein kinases [30,31,32]. Tivozanib, which is a recently registered anticancer drug based on an isoxazole scaffold, was found to be a potent ATP-competitive type II inhibitor of VEGFRs [33]. Moreover, some isoxazole derivatives have exhibited pro-apoptotic activity [34,35,36] or inhibitory activity towards tumor cell migration [37]. The results of previous studies conducted by the authors of this paper concerning the group of isoxazole derivatives have revealed many compounds with immunomodulatory—including immunosuppressive, immunostimulatory, and anti-inflammatory—activities [38,39,40,41,42,43,44,45,46].

Because previous studies on the biological properties of derivatives of the oxazolo[5,4-*d*]pyrimidine system indicate that these compounds exhibit a wide range of diverse bioactivities, they are therefore a valuable source in the search for potential drugs. Given the above, the present paper aimed to design, synthesize, and carry out the biological testing of a few new oxazolo[5,4-*d*]pyrimidine derivatives with potential anticancer activity as possible antimetabolites (due to their structural similarity to purines) and/or, presumably, as potential inhibitors of human VEGFR-2. According to the results of research conducted by the authors on a previously reported series of 7-aminooxazolo[5,4-*d*]pyrimidines (**SCM1-10**) that have shown immunosuppressive, antiviral, and anticancer activities [18], the newly designed compounds also contain a pharmacologically favorable 5-amino-3-methyl-isoxazole moiety at position 2. However, the main difference between the previous and current series of the compounds is the presence of the methyl group at position 5 of the oxazolo[5,4-*d*]pyrimidine system in **SCM1–10** derivatives. It seems reasonable to suppose that replacing the methyl group with a proton in newly designed compounds may improve their anticancer effects. Four cancer cell lines were selected to test the activity of new derivatives in vitro, namely, lung carcinoma (A549), breast adenocarcinoma (MCF7), metastatic colon adenocarcinoma (LoVo), and primary colon adenocarcinoma (HT29). Cisplatin and 5-fluorouracil (5-FU) were chosen as reference drugs for this study because they show potent anticancer activity against the cancer cell lines used in this research. The long-established antimetabolic anticancer drug 5-fluorouracil, which acts as an antagonist of pyrimidine nucleic bases, is mainly administered to treat gastrointestinal cancers such as colorectal cancer, esophageal cancer, stomach cancer, and pancreatic cancer, but also breast cancer and cervical cancer. Moreover, 5-fluorouracil is similar to designed oxazolo[5,4-*d*]pyrimidines by analogy as an antagonist (antimetabolite) of nucleic bases. Additionally, the report stating that 5-FU inhibits VEGF-mediated angiogenesis is noteworthy [47]. According to Reddy et al. [48], 5-FU interacts with the VEGFR-2 active site through the formation of conventional hydrogen bonds by the –NH and carbonyl groups of the uracil moiety with Glu885 (2.37 Å) and Asp1046 (2.03 Å), respectively. Cisplatin, on the other hand, is particularly effective in treating various types of lung cancer [49].

## 2. Results

### 2.1. Synthesis of Oxazolo[5,4-d]pyrimidines ***3a–j***

**Scheme 1:**
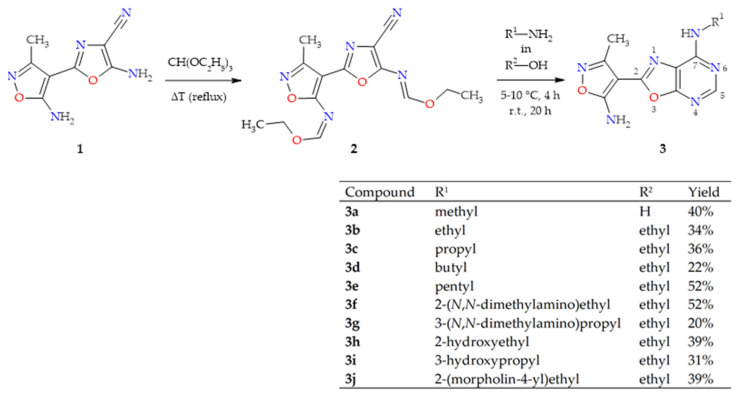
Synthetic pathway for the preparation of new oxazolo[5,4-*d*]pyrimidines **3a–j**. r.t.: room temperature.

Generally, an oxazolo[5,4-*d*]pyrimidine scaffold can be constructed starting from a functionalized oxazole or pyrimidine. Since a C(2)-functionalized 5-aminooxazole-4-carbonitrile is a versatile building block for the construction of different oxazolo[5,4-*d*]pyrimidines, the novel oxazolo[5,4-*d*]pyrimidine derivatives were synthesized via formation of the pyrimidine ring in the two-step synthetic pathway outlined in Scheme 1.

The synthesis of substrate **1**—i.e., 5-amino-2-(5-amino-3-methylisoxazol-4-yl)-oxazole-4-carbonitrile—was carried out using a multistep method described in detail previously [18]. The reaction of oxazole **1** with triethyl orthoformate in reflux made it possible to obtain intermediate imidoester derivative **2** with a moderate yield. Then, ring closure with an aqueous solution of methylamine allowed us to obtain an oxazolo[5,4-*d*]pyrimidine in which the 7-amino group is replaced with methyl. Instead of the aqueous solution, ethanol solutions of selected primary amines were used to obtain other derivatives of the desired oxazolo[5,4-*d*]pyrimidines with low-to-moderate yields. It is necessary to underline that the low yield of the reactions of imidoester **2** with amines was attributed to the facile decomposition of compound **2** to substrate **1**. The structures of all of the new compounds were determined with the use of spectroscopic methods such as MS, IR, and NMR (for details, see Section 4 (Materials and Methods), Section 4.1 (Chemistry)).

### 2.2. In Vitro Cytotoxicity

The cytotoxic activity of 10 newly synthesized oxazolo[5,4-*d*]pyrimidine derivatives (**3a–j**) against two normal cell lines—i.e., murine fibroblasts (L929) and normal human dermal fibroblasts (NHDF)—and a panel of four human cancer cell lines—i.e., lung adenocarcinoma (A549), breast adenocarcinoma (MCF7), metastatic colon adenocarcinoma (LoVo), and primary colon adenocarcinoma (HT29)—was evaluated in vitro using the standard MTT method based on viability assay. The reference drugs selected for the study were 5-fluorouracil (5-FU) and cisplatin. The estimated half-maximal cytotoxic concentrations (CC_50_) for compounds **3a–j** and the reference drugs are presented in Table 1. Regarding toxicity against NHDF cells, it was observed that oxazolo[5,4-*d*]pyrimidines **3a–b** and **3h–j** were non-toxic. Compounds **3e–f** had low toxicity against NHDF, with CC_50_ values ranging from 124.65 to 171.81 µM. Generally, all of the tested compounds were much less toxic against healthy NHDF cells than the reference drugs. The results of cytotoxicity tests against the L929 cell line revealed that only two compounds—namely, **3a** and **3d**—showed no toxic activity. Only the cytotoxicity of **3j** in a test against L929 cells appeared to be comparable with that of the reference drugs. The rest of the compounds appeared to be statistically less toxic than the reference drugs against L929 cells.

Investigation of cytotoxicity results against the HT29 colorectal adenocarcinoma line indicated that the CC_50_ of the tested oxazolo[5,4-*d*]pyrimidines ranged from 58.44 to 224.32 µM in comparison with 5-FU and cisplatin, the CC_50_ values of which were 381.16 ± 25.51 and 47.17 ± 7.43 µM, respectively. The compounds with hydroxyalkyl substituents (i.e., **3h** and **3i**) appeared to lack cytotoxic activity against the tested cancer cell lines. In the group of compounds active against the HT29 line, the derivatives **3g** (with a 3-(*N*,*N*-dimethylamino)propyl substituent), **3j** (with a 2-(morpholin-4-ylo)ethyl substituent), and **3e** (with a pentyl substituent) were particularly noteworthy, for which the cytotoxic activity (CC_50_) was 58.44 ± 8.75, 99.87 ± 10.90, and 129.41 ± 10.04 µM, respectively. These three compounds (i.e., **3e**, **3g**, and **3j**) have aliphatic substituents at position 7 of the oxazolo[5,4-*d*]pyrimidine system of a comparable length, but significantly different relative lipophilicity (see the calculated values of LogP in Table S21 in the Supplementary Materials), which enabled the evaluation of the effect of chain hydrophobicity on cytotoxic activity. Of course, it should be made clear that the LogP values calculated alone do not have greater worth without their experimental verification. However, for comparison purposes, the calculated values of LogP can be taken as relative between the compared compounds and may indicate whether an increase or decrease in lipophilicity is beneficial for the activity of the considered group of compounds. It should be emphasized that statistical analysis of the results showed that there was no statistically significant difference in cytotoxicity (CC_50_) between compound **3g** (58.44 ± 8.75 μM) and cisplatin (47.17 ± 7.43 μM) regarding the HT29 cell line, so it can be concluded that both of them have equal activity against HT29 cells and that it is the **3g** derivative that is the most active against HT29in this oxazolo[5,4-*d*]pyrimidine series. Moreover, **3g** was significantly more active than 5-FU against this cancer. It should also be noted that compound **3g** had a much better selectivity index (SI ≈ 3) for the HT29 line than the reference drugs, which were much more toxic to healthy human cells (NHDFs) than to cancer cells, and whose selectivity indexes (SIs) were well below 1 in this case. It is also important to emphasize that the difference in CC_50_ between the NHDF and HT29 cell lines was statistically significant for compound **3g**, which means almost three times less toxicity to healthy NHDF cells than to HT29 cancer cells. Less active than compound **3g**, albeit the most active among the rest compounds, were the derivatives **3j** and **3e,** whose cytotoxicity (CC_50_) was comparable (99.87 ± 10.90 μM and 129.41 ± 10.04 μM for **3j** and **3e,** respectively). However, although **3j** was slightly less active than **3g** against HT29 cancer cells, compound **3j** had the best selectivity index (SI) in this test, as it showed no toxicity at all against healthy NHDF cells at concentrations up to 500 μM, and its CC_50_ value against HT29 cells was 99.87 ± 10.90 μM. On the other hand, compound **3e** (with a pentyl group), despite showing activity similar to **3j**, was equally toxic to healthy cells and cancer cells (i.e., no statistically significant differences between means—CC_50_). Hence, **3e** had the worst selectivity index (SI ≈ 1) among the three considered derivatives (**3e**, **3g,** and **3j**). In addition, it is also worth noting that compound **3g**, which is the most active of these three compounds, has an aliphatic chain at position 7 of the oxazolo[5,4-*d*]pyrimidine system, with intermediate lipophilicity relative to the weaker compounds **3e** and **3j** (see the calculated values of LogP in Table S21 in the Supplementary Materials). The other compounds (**3a–d,f**) active against HT29 were found to be significantly weaker than cisplatin, but all of them were statistically significantly stronger than 5-FU.

In the case of the A549 cell line, four compounds (**3d**–**g**) showed statistically significantly stronger cytotoxic activity than the reference drug 5-FU but were much weaker than cisplatin. The three best compounds of the series—i.e., **3e**, **3f,** and **3g**—had comparable activity against lung cancer cells (A549). The activity of compound **3a** was comparable with that of 5-FU. The remaining five compounds (i.e., **3b**, **3c**, **3h–j**) showed no activity against A549.

In the case of the MCF7 cell line, two oxazolo[5,4-*d*]pyrimidines (i.e., **3a** and **3h**) showed no cytotoxic activity, and the rest of the tested compounds appeared to be much less active than the reference cisplatin, but two of them with lipophilic butyl and pentyl substituents (i.e., **3d** and **3e**, respectively) showed statistically better activity than 5-FU.

In contrast to the encouraging results of the tested oxazolo[5,4-*d*]pyrimidines in terms of their activity against primary colon adenocarcinoma cells (HT29), they showed much weaker or no activity against metastatic colon adenocarcinoma cells (LoVo) in comparison with both reference drugs. Compound **3e,** with a lipophilic pentyl substituent, showed the highest cytotoxicity (CC_50_ = 177.52 ± 6.65 μM) against LoVo colon cancer cells in the group of the analyzed oxazolo[5,4-*d*]pyrimidines and, despite its weaker activity compared to both reference drugs, this compound had a slightly better selectivity index (SI = 0.702) than both reference drugs (SI = 0.419 and 0.282 for cisplatin and 5-fluorouracil, respectively) due to significantly lower toxicity relative to the reference drugs. Generally, the reference drugs showed statistically significantly stronger cytotoxic activity against healthy NHDF cells than against cancer cells in all of the performed tests. Therefore, they have an unfavorable selectivity index (SI < 1). The only exception was cisplatin, whose cytotoxicity (CC_50_ = 8.17 ± 2.39) against A549 cells was statistically equal to its cytotoxicity (CC_50_ = 11.49 ± 3.02) against healthy NHDF cells (hence, SI ≈ 1).

In conclusion, the significantly lower toxicity of the tested compounds against healthy NHDF cells compared to the reference drugs, along with their better selectivity indices, put the tested oxazolo[5,4-*d*]pyrimidine derivatives in a favorable light compared to the reference drugs.

### 2.3. P-glycoprotein-Inhibitory Ability

**Figure 4:**
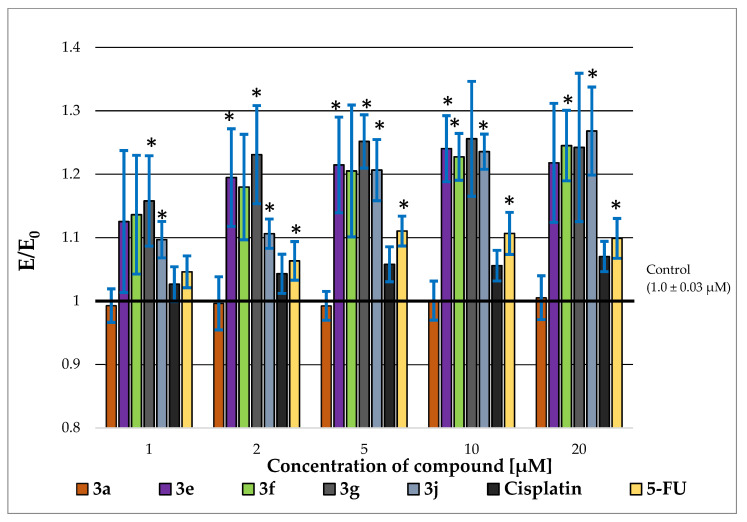
Rhodamine accumulation after incubation with the tested compounds at various concentration ranges in HT29 cells; * *p* < 0.05—significant difference compared to the negative control. E/E_0_—E is the result for the measured sample, and E_0_ is the result for the control.

It has been reported that P-glycoprotein (P-gp) is a membrane transporter functioning as a drug efflux pump. Encoded by the multidrug resistance (MDR) 1 gene, P-gp is overexpressed in many human cancer cells, contributing to multidrug resistance and representing a major cause of cancer treatment failure. In vitro measurement of the P-gp-inhibitory potential of drug candidates is important in the context of their evaluation in terms of multidrug resistance reversal activity and subsequent drug–drug interaction [50]. For selected compounds—i.e., **3a**, **3e**, **3f**, **3g**, and **3j**—and reference cytostatics, the rhodamine-123 (Rh-123) accumulation assay was carried out in order to evaluate their effects on the efflux activity of P-gp in HT29 cells. In this study, variation in cellular fluorescence was determined in the presence of selected derivatives—cisplatin and 5-fluorouracil—allowing the evaluation of their P-gp-inhibitory ability. As shown in Figure 4, the fluorescence intensity of Rh-123 was higher for compounds **3e–f** and **3j** in comparison to control cells and the reference drugs. Compound **3g** was found to demonstrate the highest increase in Rh-123 accumulation at concentrations of 1 μM (E/E0 = 1.15), 2 μM (E/E0 = 1.23), 5 μM (E/E0 = 1.25), and 10 µM (E/E0 = 1.25) (see Table S14 in the Supplementary Materials). The highest accumulation of rhodamine-123 occurred after the incubation of the HT29 colon adenocarcinoma cell line at a concentration of 20 μM for compound **3j** (E/E0 = 1.26). For compound **3a**, there was no intracellular increase in rhodamine accumulation. In the case of compounds **3e** and **3g**, a reduction in the accumulation of rhodamine at the highest tested concentration (20 µM) was observed compared to the concentration of 10 µM, which may have been related to the reduced number of living cells (accumulation of rhodamine is possible only in living cells, as well as when the P-glycoprotein receptor is saturated with the test compounds—which occurs already at a concentration of 10 µM). On the other hand, for the compounds **3f**, **3j,** and the used cytostatics, a concentration-dependent increase in the accumulation of rhodamine-123 was observed. It is expected that the increase in expression of the rhodamine dye in the context of assessing P-glycoprotein activity will be at least twofold. However, Cheraghi et al. have shown that treating HT29 cells with conferone for 24 h, 48 h, and 72 h affects the accumulation of Rh-123 [51]. In their study, they used verapamil as a positive control. The increase in Rh-123 accumulation of both the test compound and verapamil in HT29 cultures was not particularly strong after 24 h. The intracellular fluorescence intensity of Rh-123 after treatment with verapamil for 48 h and 72 h was increased 1.77- and 1.9-fold, respectively [51]. Our study observed an intracellular increase in rhodamine accumulation of no more than 1.25-fold after 24 h of incubation with the tested compounds, cisplatin, or 5-FU. Due to the low intracellular accumulation of rhodamine and its slight increase in the subsequent concentrations of the tested compounds, additional studies should be carried out (e.g., Multidrug-Resistance-Related Protein 1 Expression Assay)—especially for compounds **3e–3j**—before selecting a compound for further in vivo studies.

### 2.4. Pro-Apoptotic Activity

**Figure 5:**
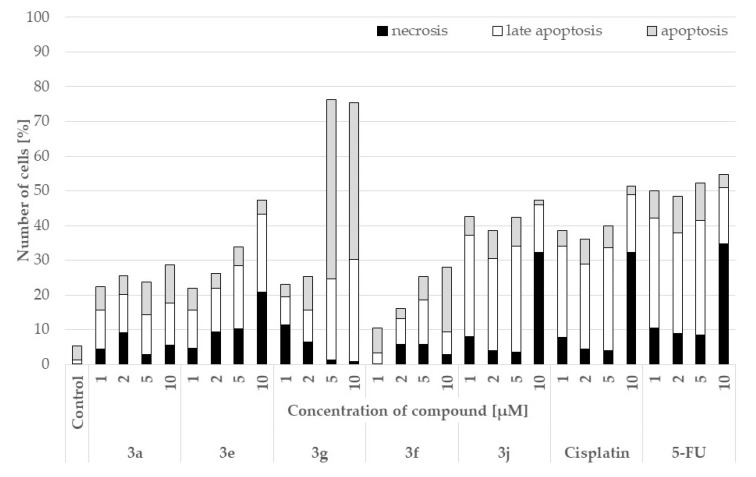
Apoptosis in cells treated with 1, 2, 5, and 10 µM concentrations of compounds **3a**, **3e**, **3f**, **3g,** and **3j** for 24 h in comparison to cisplatin and 5-fluorouracil.

Since apoptosis is a process of programmed cell death, many anticancer drugs are designed to induce tumor cell apoptosis [52]. In contrast, necrosis is an irreversible cell injury that results in uncontrolled cell death along with an inflammatory response in the surrounding tissues. This process is often recognized as a side effect of using anticancer agents for the treatment of various types of cancer [53]. An increase in the frequency of apoptosis by 50% after 24 h was determined for five of the selected compounds—i.e., **3a**, **3e**, **3f**, **3g,** and **3j**—and the reference drugs. The apoptosis assay results are illustrated in Figure 5 and presented in Table S15 in the Supplementary Materials. All of the tested compounds exhibited pro-apoptotic activity, although for the derivatives **3a**, **3e,** and **3j** it was weaker than for the reference drugs. Moreover, compound **3j** at a concentration of 10 μM (32.35) showed an effect on cell necrosis at a level similar to both cytostatics (cisplatin = 32.35 and 5-FU = 34.82). As can be seen, for compound **3g** (for concentrations of 5 μM—51.54 and 10 μM—45.24), there was a strong increase in cell apoptosis compared to the reference drugs (cisplatin: 6.28, 2.34; 5-FU: 10.75, 3.81, respectively). The results also indicated only a weak effect of compound **3g** on cell necrosis at a concentration of 10 μM (0.90). Further evaluation was carried out to explore the mechanism of apoptosis induced by compound **3g**.

### 2.5. Effect on Cell Migration

**Figure 6:**
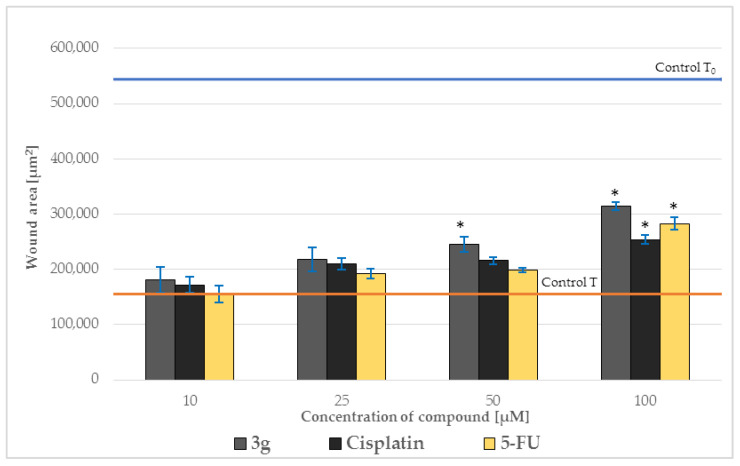
Effects of the tested compounds on the migration of cells in the scratch assay after 24 h of incubation for the HT29 cell line. Control T_0_ is the width of the scratch, i.e., the wound at the beginning before the administration of the compound (T_0_ = 544,366.45 ± 22,895.3). Control T is the width of the wound when no compound was administered (T = 155,558.2 ± 35,476.2). * *p* < 0.05—significant difference compared to the negative control.

**Figure 7:**
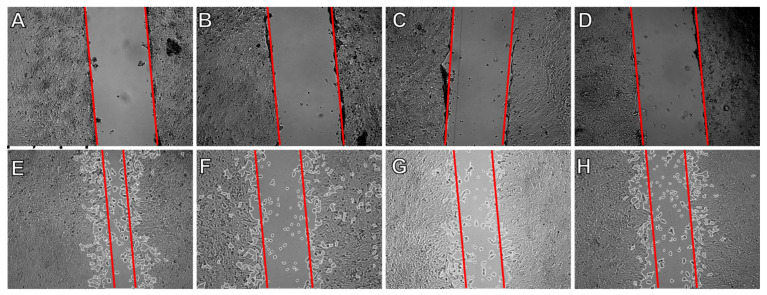
Scratch tests: (**A**) control T_0_; (**B**) compound 3g T_0_; (**C**) cisplatin T_0_; (**D**) 5-FU T_0_; (**E**) control T; (**F**) compound **3g** T; (**G**) cisplatin T; (**H**) 5-FU T. Compound **3g**, cisplatin, and 5-FU at concentrations of 100 µM. The red lines indicate the width of the scratch. Microscope magnification was set at 4×.

Cell migration is a premise to evaluate cancer for invasion and metastasis, suggesting cell motility as a potential therapeutic target for cancer treatment [37]. Cell migration was tested for the most active compound (i.e., **3g**) and the reference drugs (i.e., 5-fluorouracil and cisplatin). The scratch test was performed to verify that the tested compound **3g** could inhibit the formation of metastases by the HT29 cell line. The seeded HT29 cells were incubated until they formed a monolayer over the entire surface of the well. Then, a scratch test was performed by making a scratch in the monolayer and measuring its width—that is, T_o_, amounting to 544,366.45 μm^2^. Freshly prepared compounds were added to the HT29 cells at concentrations of 10, 25, 50, and 100 μM. Breeding plates were incubated for 24 h. The next day, scratch width was measured again using the ImageJ open-source platform. With the increase in the concentration of the reference drugs and compound **3g**, inhibition of the migration of HT29 cells also increased (Figure 6 and Table S16 in the Supplementary Materials). The lowest cellular overgrowth of HT29 culture occurred at the concentration of 100 μM for the compound. Compound **3g** inhibited the migration of HT29 cells more strongly than the reference drugs, showing that it has the potential to inhibit metastasis. The width of the scratch after 24 h when no compound was applied to the cells was used as a control (T = 155,558.2 μm^2^). The photos from the scratch assay are presented in Figure 7.

### 2.6. Effects on Levels of p53, Caspase-3, and BCL-2

Apoptotic markers—namely, the p53 protein and caspase-3—mediate the process of programmed cell death, which limits the process of neoplasia. The p53 protein is actively involved in both extrinsic (i.e., receptor) and intrinsic (i.e., mitochondrial) apoptosis pathways [54]. However, the intracellular pathway of cell death is mainly related to the interaction of the p53 protein with proteins belonging to the BCL-2 family [55]. The p53 protein also plays an important role in the induction of apoptosis mediated by TNFR1, which includes death receptors (DRs). It is the formation of a complex consisting of the DR death domain with the FADD adapter protein and initiating caspases such as caspase-8 or -10 that leads to the direct activation of the effector caspase-3 and, consequently, the initiation of the irreversible process of cell death [56,57]. Caspase-3 in particular is the proteolytic enzyme responsible for the intracellular induction of an apoptosis-inducing signal [58]. In contrast, BCL-2 is a pro-survival (i.e., anti-apoptotic) protein that suppresses cell death by inhibiting the release of apoptosis regulators such as cytochrome c [59]. The concentrations of p53, caspase-3, and BCL-2 were analyzed in precise quantitative measurements with the use of appropriate human enzyme-linked immunosorbent assay (ELISA) kits for the most potent cytotoxic compound **3g** in HT29 cells (Table 2). In the results of the ELISA tests, the concentration of BCL-2 was the lowest compared to caspase-3 and p53. Compound **3g** caused the highest concentration of caspase-3 which, compared to the levels of the other tested markers, leads to the conclusion that said compound induces the apoptosis process to the greatest extent, i.e., through the extracellular pathways.

### 2.7. Molecular Docking

As was mentioned in the Introduction, the overexpression of vascular endothelial growth factor receptor-2 is often related to different types of tumors [5]. In consequence, one of the strategies for treating cancer is using VEGFR-2-targeted compounds. According to previous studies, oxazolo[5,4-*d*]pyrimidine derivatives were found to be inhibitors of VEGFR-2 [6,7,8,9]. Therefore, in the present paper, an attempt was made to predict the binding mode of the designed ligands to the active site of VEGFR-2 using molecular docking. The validation of the docking procedure was performed by docking the co-crystalized ligand tivozanib to the binding site of VEGFR-2, as described in detail in previous studies [60,61]. Due to its ability to bind in the hinge region and hydrophobicity region of the protein (HYD-II)—also referred to as an allosteric pocket—tivozanib was classified as a type II selective inhibitor of VEGFR-2 [33]. Such compounds are able to bind to the kinase in both an ATP-binding site and an adjacent hydrophobic pocket formed by the activation loop. They form 1–3 hydrogen bonds with the amino acid residues (i.e., Glu885, Glu917, and Cys919) located in the hinge region of the protein. Additionally, they bind to the allosteric pocket formed by hydrophobic amino acids, namely, Leu889, Ile892, Val898, and Ile1044. Inhibitors of this type were proven to have higher efficacy and selectivity compared to type I inhibitors.

According to the results of this study, all of the designed oxazolo[5,4-*d*]pyrimidine derivatives are able to bind to the binding site of VEGFR-2, with free energies of binding between −38.5. and −47.3 kJ/mol. The scoring functions—including in terms of free energy of binding (∆G_binding_), inhibition constants (K_i_) rated for the best docking conformations, and intermolecular interaction—are presented in Table 3. As can be expected, the geometry of the inhibitors affects their binding properties and inhibitory activity. The present paper offers a description of the properties observed for compounds that were selected for extended biological studies (i.e., **3a**, **3e–g**, **3j**).

**Figure 8:**
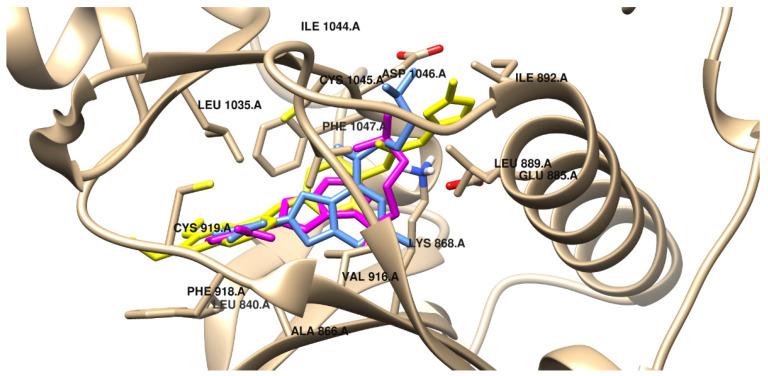
The binding pose of the most potent designed inhibitor **3g** (magenta), the 5-methyl analog of compound **3g** (**SCM9**) (blue), and tivozanib (yellow) in the active center of VEGFR-2.

**Figure 9:**
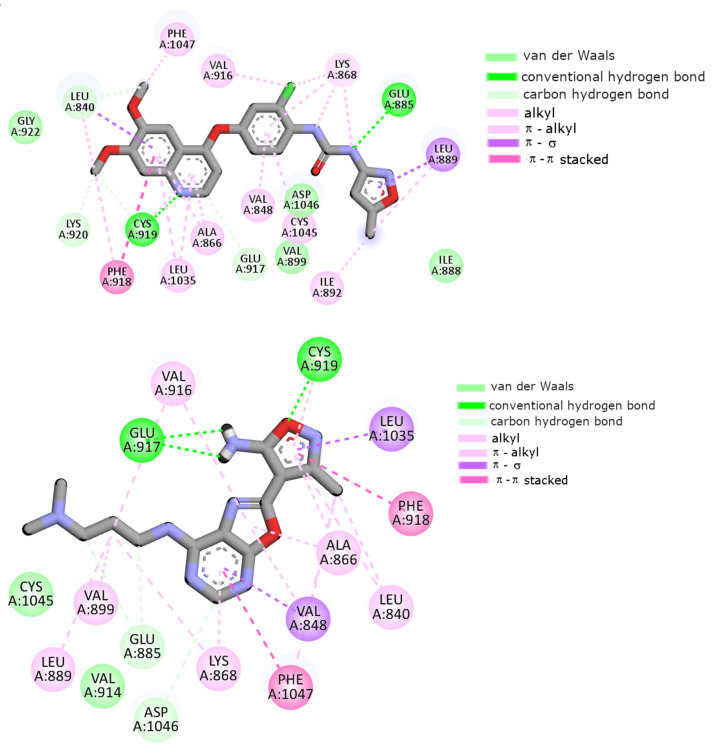

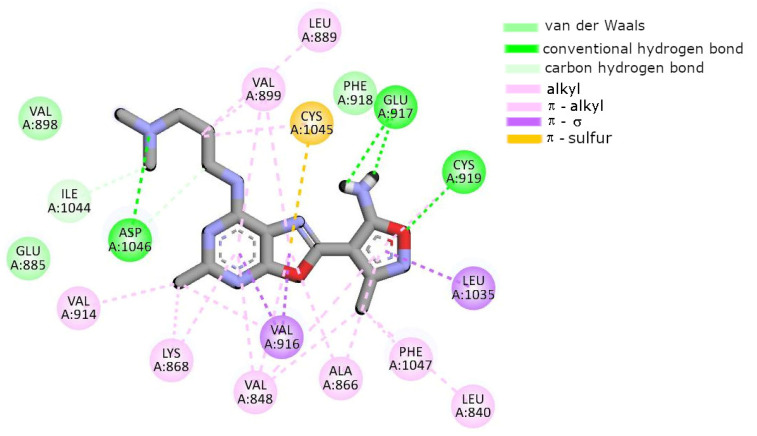
The comparison of the intermolecular interactions of the drug tivozanib, compound **3g**, and the 5-methyl analog of compound **3g** (**SCM9**) (shown from top to bottom, respectively) with the active center of VEGFR-2.

As presented in Figure 8 and Figure 9, tivozanib was involved in π–π stacking interactions with Phe918 via the 6,7-dimethoxyquinoline ring of the drug molecule. One hydrogen bond was formed between Cys919 and the nitrogen atom of the dimethoxyquinoline ring, while π–alkyl interactions were established with Ala866, Leu1035, and Phe1047. The interactions with Phe918 and Cys919 amino acid residues seemed to be very important for the active conformation of tivozanib. The 2-chloro-oxyphenyl moiety was localized in the pocket formed by Val848, Val899, Val916, Cys1045, and Asp1046 and interacted via van der Waals and π–alkyl forces (see Table 3 for details). Moreover, the isoxazole moiety was involved in a unique interaction with Leu889 in the HYD-II region of the receptor. These results are consistent with the previous data obtained for tivozanib [60].

According to the results of molecular docking, the most potent inhibitor was compound **3j** (estimated K_i_ = 0.04 μM). As can be observed in this case, the 2-(morpholin-4-yl)ethyl moiety interacts through hydrogen bonding and van der Waals forces with Asp1046 and Leu889 (see Figure S48 in the Supplementary Materials and Table 3). The pyrimidine ring can form π–alkyl and π–σ interactions with the Ala866, Lys868, and Val848 amino acid residues. Next, a hydrogen bond between the oxygen heteroatom of isoxazole and Cys919 is created. Additionally, similar to tivozanib, this part of the molecule is stabilized through π–π stacking interactions with Phe918 and alkyl interactions with Leu840, Ala866, and Leu840 amino acid residues.

A similar mode of binding of the isoxazole moiety is exhibited by compounds **3a**, **3e**, and **3g** (see Figures S45 and S46 in the Supplementary Materials, as well as Figure 9 and Table 3). In all considered cases, hydrogen bonds between Glu917 and Cys919 were formed. Furthermore, π–σ interactions and π–π stacking interactions with Leu1035 and Phe918, respectively, were also present. The results indicate that compound **3a** is the weakest inhibitor of proteins. The estimated free energy of binding, in this case, was −38.5 kJ/mol. This is clearly related to the size of the molecule. In this case, the 7-*N*-methylaminooxazolo[5,4-*d*]pyrimidine ring exhibited a unique binding configuration. The best docking pose of the inhibitor revealed van der Waals interactions with the Val867, Glu885, and Val899 amino acid residues. The inhibitor was also stabilized in the active center of VEGFR-2 via π–alkyl and alkyl interactions with Leu840, Val848, Ala866, Lys868, Val916, Leu1035, and Cys1045. In the case of compound **3e**, a pentyl substituent is surrounded mainly by hydrophobic amino acid residues and interacts with protein through van der Waals and alkyl forces. The oxazolo[5,4-*d*]pyrimidine rings are stabilized in the binding cavity of VEGFR-2 by π–π stacking interactions with Phe1047 and π–alkyl interactions with Val848 and Lys868. An important role in stabilization is also played by hydrogen bonds formed in the hinge region with the Glu917 and Cys919 amino acid residues. The binding pose of compound **3f** is presented in Figure S47 in the Supplementary Materials. As can be seen, oxazole and pyrimidine rings interact via π–π stacking with Phe1047 and π–σ interactions with Leu1035. The 2-(*N*,*N*-dimethylamino)ethyl chain is localized in the hydrophobic cavity and stabilized mainly by van der Waals interactions. The intermolecular interactions of compound **3g** with VEGFR-2 are presented in Figure 9. The π–σ interactions with Leu1035 and Val848 play a role in the stabilization of the oxazole and pyrimidine rings, respectively. Similar to tivozanib, compound **3g** is involved in π–π stacking interactions with Phe918 and forms a hydrogen bond with Cys919 via the isoxazole moiety. The aliphatic part of the molecule penetrates the allosteric pocket formed by Val899, Val916, Cys1045, and Asp1046 and interacts with it through van der Waals and alkyl forces.

### 2.8. Physicochemical Properties, Pharmacokinetics, and ADME Activity

The physicochemical properties, pharmacokinetics, and ADME (adsorption, distribution, metabolisms and excretion) activity of compounds **3a**, **3e–g**, and **3j** were estimated based on the comprehensive database ADMETlab (2.0) [62]. As can be seen in Table S18 in the Supplementary Materials, all of the considered compounds can be absorbed orally because certain rules are met (e.g., Lipinski rules, Pfizer rules, GSK rules). According to data, the molecular weight of the compounds does not exceed 500 Da, the decimal logarithm of the octanol/water coefficient at physiological pH (i.e., LogD) is less than 3, the number of hydrogen bonds is ≤10, the number of hydrogen bond donors is ≤5, and the TPSA (topological polar surface area) is less than 140 Å^2^ (see Table S17 in the Supplementary Materials). In addition, all of the compounds exhibit good bioavailability, plasma protein index binding below 85%, and low blood–brain barrier (BBB) permeability (see Table S19 in the Supplementary Materials). As shown by the results presented in Table S20 in the Supplementary Materials, compounds **3e–g** and **3j** are potential inhibitors of two enzymes from the cytochrome P450 superfamily, i.e., CYP1A2 and CYP3A4. Theoretical predictions indicate that all of the investigated compounds should show no inhibitory activity towards CYP2C19, CYP2C9, and CYP2D6, which are isozymes of cytochrome P450 involved in the metabolism of a wide array of medications in the liver, including anti-inflammatory drugs, oral hypoglycemics, angiotensin II blockers, proton-pump inhibitors, anti-epileptics, antidepressants, antipsychotics, beta-blockers, antiretroviral agents, and anti-arrhythmics [63].

## 3. Discussion and Conclusions

The new isoxazole-substituted oxazolo[5,4-*d*]pyrimidine derivatives **3a–j** were designed, synthesized, and characterized using spectral analysis, including ^1^H-NMR, ^13^C-NMR, HR-MS, and IR methods. The analytical data were in good agreement with the structures of the obtained compounds. Then, derivatives **3a–j** were screened in vitro for their cytotoxic activity against four human cancer cell lines, i.e., lung carcinoma (A549), breast adenocarcinoma (MCF7), colon cancer (LoVo), and colon adenocarcinoma (HT29). The obtained results indicated that the primary colon cancer (HT-29) cell line was the most sensitive to the influence of new oxazolo[5,4-*d*]pyrimidines. Conversely, the LoVo cell line, derived from metastatic colon cancer, was the most resistant to the effects of the tested derivatives. Compound **3g** (with a 3-(*N*,*N*-dimethylamino)propyl substituent) in particular was found to be the most potent, especially against the HT29 cell line, with a CC_50_ value of 58.44 ± 8.75 µM in comparison to the reference drugs—namely, cisplatin and 5-fluorouracil, with CC_50_ values of 47.17 ± 7.43 and 381.16 ± 25.51 µM, respectively. Hydroxylalkyl-containing compounds (i.e., **3h** and **3i**) did not exhibit considerable cytotoxic activity. The results obtained were very well correlated with a previous study conducted by the authors on the 7-aminoxazolo[5,4-*d*]pyrimidine derivatives **SCM1–10**, where the most favorable cytotoxic activity of the compounds was also observed for the HT-29 cell line [18]. Similar to the results of the current study, the most active compound overall in the series **SCM1–10** was a derivative with a 3-(*N*,*N*-dimethylamino)propyl substituent [18].

The compounds most active (**3a**, **3e–g**, and **3j**) against primary colon adenocarcinoma (HT29) were further evaluated for their P-glycoprotein (P-gp)-inhibitory ability and pro-apoptotic activity. The obtained results releveled that, generally, all of the tested compounds demonstrated the ability to inhibit P-gp, as well as pro-apoptotic activity. In particular, the oxazolopyrimidine derivative **3g** was found to be the most potent among the selected compounds. The analysis of the mechanism of action of compound **3g** indicated that it induces cell death through the extracellular pathway of apoptosis, characterized by a low level of the anti-apoptotic protein BCL-2 and a high level of the pro-apoptotic factor caspase-3. This study also found that compound **3g** potently inhibited HT29 cells’ migration. The compounds that exhibited the best cytotoxic activity in the series previously analyzed by the authors—**SCM1–10** [18]—were also investigated for their effects on apoptosis. The study was performed on the Jurkat T-cell model of the expression of signaling molecules that regulate apoptosis (including BCL2, caspase-3, and the p53 protein). The 5-methyl analog of compound **3e** (**SCM5**) completely blocked the synthesis of the anti-apoptotic protein BCL-2, in contrast to the 5-methyl analog of compound **3g** (**SCM9**), which did not alter the expression of apoptosis-modulating proteins. However, compound **SCM9** increased the expression of components of mitogen-activated protein kinase (MAPK) pathways such as JNK, p38α, and p38β [18].

**Figure 10:**
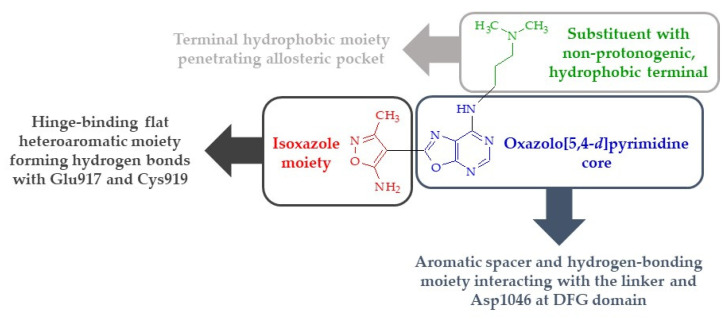
The main pharmacophoric features of newly designed VEGFR-2 inhibitors **3a–j**, as exemplified by the most active compound **3g**, developed on the basis of the theoretical study.

In the present work, a well-established molecular target (i.e., VEGFR-2) was chosen to analyze the docking interaction. Linking an isoxazole moiety with an oxazolo[5,4-*d*]pyrimidine core bearing different hydrophobic groups at position 7 to one covalently bonded hybrid compound afforded a new scaffold for the potential inhibition of VEGFR-2 (Figure 10). The design was based on the relevant pharmacophoric features of tivozanib, which is a type II VEGFR inhibitor used in the treatment of advanced renal cell carcinoma [33]. The obtained results of the theoretical study revealed that all of the designed compounds similar to the selected type II VEGFR-2 inhibitors should be able to bind to the linker (Ala866, Val914, Leu1035, and Cys1045) and HYD-II regions of the protein. In the hinge region, they should be able to form two hydrogen bonds with Glu917 and Cys919 (except for compound **3f**). Interestingly, an isoxazole moiety was fixed to be the hinge-binding head of all of the analyzed derivatives, in contrast to tivozanib. The oxazolo[5,4-*d*]pyrimidine core should be involved in interactions with VEGFR-2 in the linker and DFG domain, which is composed of the highly conserved triad Asp-Phe-Gly, and its role is to link the hinge-binding head (i.e., the isoxazole moiety) with the hydrophobic terminal of the substituent at position 7 of the oxazolo[5,4-*d*]pyrimidine core. The methine group, which is the fifth position of the oxazolo[5,4-*d*]pyrimidine system, acting as a hydrogen bond donor (HBD), may be especially involved in interaction with Asp1046, which forms an important part of the triad. Various substituents (here also referred to as tails) at position 7 can form van der Waals and other hydrophobic interactions with the allosteric pocket. Overall, compound **3g** should interact via hydrogen bonds with key amino acid residues at the active site of the receptor—i.e., Glu917, Cys919, Asp1046, and Glu885—as with most of the developed VEGFR-2 inhibitors. In contrast, its 5-methyl analog (**SCM9**) showed a different binding pattern with the DFG motif and the allosteric pocket of VEGFR-2 (Figure 9) and was characterized by a weaker inhibition constant (estimated K_i_ = 0.11 µM) compared to compound **3g**. It should be emphasized that **SCM9** lacked hydrogen bonds with the Glu885 residue—known as an essential feature of type II VEGFR-2 inhibitors. However, the above theoretical considerations must be confirmed experimentally to prove the mechanisms of activity of the tested compounds.

Data obtained from the literature indicate that well-known drugs targeting VEGFRs exhibit half-maximal inhibitory concentrations (IC_50_) ranging from 0.19 ± 0.04 to 431 µM (or undetermined) against the A549, MCF7, LoVo, and HT29 cell lines (see Table 4). In addition, one drug (lenvatinib) had almost no effect on the growth of A549 cells. It should be emphasized that none of the VEGFR inhibitors are registered by the Food and Drug Administration (FDA) and European Medicines Agency (EMA) for the treatment of lung adenocarcinoma or breast cancer, and only regorafenib is approved by the FDA and EMA for the treatment of colon cancer. It should also be noted that, overall, the tested compounds **3a–j** showed weaker cytotoxicity activity against the tested tumor cell lines than the VEGFR-2 inhibitors, except for lenvatinib, which was much weaker than the tested compounds against HT29 cells. Of course, whether or not the compounds are more active compared to the activity of the reference drugs does not provide any evidence as to whether or not their mechanism of action is identical to that of the reference drugs.

The structure–activity relationship (SAR) study, which was conducted amongst the newly synthesized oxazolo[5,4-*d*]pyrimidines, was closely investigated with a focus on the effects of changes in the substituent at position 7. The obtained results indicate clearly that the activity of the tested compounds depends mainly on the appropriate size (e.g., length) of the substituent, as well as the presence of appropriate functional groups in it. It was found that compounds **3a**, **3b**, and **3c,** bearing aliphatic substituents consisting of one, two, and three carbon atoms, respectively, as a terminal hydrophobic moiety, were less preferred biologically and showed a lack of (or relatively weak) cytotoxic activity against all of the selected cancer cell lines. The presence of a proton donor hydroxyl group (OH) at the end of the substituent in compounds **3h** and **3i** was also responsible for the lack of cytotoxic activity. In contrast, it was revealed that the incorporation of 2-(morpholin-4-yl)ethylamino (length: seven atoms), 3-(*N*,*N*-dimethylamino)propylamino (length: six atoms), and pentylamino (length: six atoms) moieties in compounds **3j**, **3g**, and **3e**, respectively, resulted in a significant increase in cytotoxic activity against HT29 cell activity in the series of the tested oxazolo[5,4-*d*]pyrimidines. Systematic investigation of activity against the LoVo and A549 cell lines indicated that the most potent cytotoxic activity was exhibited in the case of the pentyl group in compound **3e**. Surprisingly, the 7-*N*-butyl derivative exhibited the best activity against the MCF7 cell line. Further comparison of cytotoxic activity against the HT29 cell line among the series **SCM1–10** [18] and **3a–j** revealed that the replacement of the methyl group with a hydrogen atom at position 5 of the oxazolopyrimidine core generally resulted in an increase in activity compared to the reference drug, i.e., cisplatin. It is worth noting that the 5-methyl analog of compound **3g** (i.e., **SCM9**) had activity against HT29 with CC_50_ = 39.10 ± 7.79 µM, in comparison with cisplatin with CC_50_ = 16.86 ± 1.94 µM (estimated based on data taken from reference [18]). This indicates that compound **3g** has cytotoxic activity statistically equal to that of cisplatin and that its 5-methyl derivative **SCM9** is statistically less active than cisplatin.

In summary, based on the studies performed so far, general conclusions can be drawn about the structural elements that have a significant impact on the activity of the analyzed group of compounds. Specifically, the following structural elements have a beneficial effect on the anticancer activity of the tested compounds:

1. The presence of a substituent of appropriate size (i.e., a molecular tail with an optimal length of 6–7 atoms other than protons) and moderate lipophilicity at position 7 of the oxazolo[5,4-*d*]pyrimidine system.
2. The absence of proton donor groups (such as OH) at the end of this substituent at position 7 of the oxazolo[5,4-*d*]pyrimidine system. If there is a strongly electronegative element (such as oxygen or nitrogen) at the terminal of this tail, then protons connected to this atom should be replaced with lipophilic substituents (such as methyl groups).
3. The presence of an acidic proton at position 5 of the oxazolo[5,4-*d*]pyrimidine system, which will allow hydrogen bonds to form at this position with receptor structures.
4. The ability of the substituent (except its terminal lipophilic fragment) at position 7 of the oxazolo[5,4-*d*]pyrimidine system to strongly interact with the Glu885 residue of the receptor active site (e.g., by forming hydrogen bonds).

The presumption of potential activity against VEGFR-2 was based on the theoretical results of docking the compounds at the active center of the receptor, so further studies should be performed to definitively confirm that the tested compounds indeed inhibit the activity of this receptor. Therefore, in order to fully investigate and elucidate the mechanism of activity of this group of compounds, further research is required and should be continued. At this stage of work, molecular docking studies indicate that the appropriate combination of isoxazole and oxazolo[5,4-*d*]pyrimidine may be an important scaffold in the development of potentially effective and selective VEGFR-2 inhibitors.

## 4. Materials and Methods

### 4.1. Chemistry

Melting points were determined using the uniMELT 2 apparatus (LLG, Meckenheim, Germany) and were uncorrected. Thin-layer chromatography (TLC) analyses were conducted using silica gel on TLC Al foils with a 254 nm fluorescent indicator (Sigma-Aldrich, Merck Group, Darmstadt, Germany) and a solvent system of eluent composed of chloroform and ethyl acetate (7:3); detection was carried out with ultraviolet (UV) light (A. KRÜSS Optronic GmbH, Hamburg, Germany). The ESI-MS (electrospray ionization mass spectroscopy) spectra were recorded with the compact^TM^ Bruker Daltonics Electrospray Ionization Quadrupole Time-of-Flight (ESI-Q-TOF) apparatus (Bruker Daltonics GmbH, Bremen, Germany). The ESI-MS experiments were conducted in positive-ion mode, and LC–MS-grade methanol was used as a solvent. Bruker Compass Data Analysis 4.2 software (Bruker Daltonics GmbH, Bremen, Germany) was used for ESI-MS spectral analysis and calculation of the theoretical monoisotopic mass of detected ions. The attenuated total reflectance IR (ATR-FT-IR) spectra (4000–450 cm^−1^) were recorded with a Nicolet iS50 FTIR spectrophotometer (Thermo Fisher Scientific Inc., Waltham, MA, USA) on a diamond crystal surface (32 scans, resolution: 1 cm^−1^, measurement temperature: 20–25 °C), using a clean solid form of the compounds, with ATR intensity correction. IR spectral analysis was performed using Omnic Spectra software (Thermo Fisher Scientific Inc., Waltham, MA, USA). Baselines of all IR spectra were corrected with autocorrection (fit order 2), and then all of the spectra were normalized. The ^1^H-NMR (300.15 MHz) and ^13^C-NMR (75.47 MHz, broadband full decoupling method) spectra were recorded using a Bruker ARX-300 spectrometer (Bruker Analytische Messtechnik GmbH, Rheinstetten, Germany). The NMR measurement experiments were carried out in deuterated dimethyl sulfoxide (DMSO-*d_6_*). ^1^H-NMR and ^13^C-NMR chemical shifts were referenced to the solvent signal, i.e., for ^1^H-NMR: δ (quintet of residual DMSO-*d_6_*) = 2.50 ppm, and for ^13^C-NMR: δ (non-decoupled septet of DMSO-*d_6_*) = 39.52 ppm. The reported data contain chemical shifts in parts per million (ppm), signal multiplicities (abbreviations used: br—broad, s—singlet, d—doublet; t—triplet, q—quartet, m—multiplet), and the number of protons. The values of the coupling constant were reported as *J* in Hz. NMR spectral analyses were performed using ACD/NMR Processor Academic Edition software (Advanced Chemistry Development, Inc., Toronto, ON, Canada) and MestReNova (Mnova version 14.2) software (Mestrelab Research S.L., Santiago de Compostela, Spain). The NMR, IR, and MS experiments were performed in the Laboratory of Elemental Analysis and Structural Research, Faculty of Pharmacy, Wroclaw Medical University. Statistical analysis of the results was performed with Statistica (data analysis software system) version 13.3 (TIBCO Software Inc., Palo Alto, CA, USA. (2017)) and PTC Mathcad Express Prime 6.0.0.0. (Copyright 2019, PTC Inc., Boston, MA, USA). The following programs were used to calculate LogP: ALOGPS 2.1 software, MarvinSketch (Chemaxon software), ChemSketch (ACDLabs software), BIOVIA Draw 2019 software, and ADMETlab software.

### 4.2. Preparation and Experimental Properties of Compounds ***2*** and ***3a–j***

#### 4.2.1. N-{4-cyano-2-[5-(ethoxymethylidene)amino-3-methylisoxazol-4-yl]oxazol-5-yl}methanimidate (**2**)

To 5-amino-2-(5-amino-3-methyl-isoxazol-4-yl)-oxazole-4-carbonitrile **1** (2.09 g, 10 mmol), 27 mL of triethyl orthoformate was added, and the reaction mixture was heated with stirring at boiling temperature (145 °C) for 10 h. Upon completion of the reaction, as shown by TLC, it was cooled to 0 °C and the formed precipitate was filtered off and dried to give compound **2** as a beige solid. Compound **2** was used for further reactions as a crude product without purification. Yield 1.976 g, 61.1%; m.p. 110–112 °C; ESI-MS: m/z[u/e] calculated for formula C_14_H_16_N_5_O_4_ [M+H]^+^ 318.1197, found 318.1237 (see Figure S1, Table S1 and Table S2 in the Supplementary Materials); ^1^H-NMR (DMSO-*d_6_*, 300 MHz): δ (ppm) 1.34 (2xt, 6H, ^3^*J* = 7.1 Hz, mutually overlapping triplets of two terminal methyl groups of ethyl fragments); 2.43 (s, 3H, CH_3_), 4.40 (q, ^3^*J* ≈ 7.0 Hz, 2H, CH_2_, the methylene group of the ethyl fragment), 4.42 (q, ^3^*J* 6.9 Hz, 2H,CH_2,,_ triplet, partially overlapped by a triplet at 4.40 ppm, of the methylene group of the ethyl fragment), 8.53 (s, 1H, CH), 8.68 (s, 1H, CH) (see Figure S12 in the Supplementary Materials); ^13^C-NMR (DMSO-*d_6_*, 75.5 MHz): δ (ppm) 11.95, 13.79, 13.83, 64.39, 64.79, 93.57, 99.61, 113.23, 150.02, 154.30, 157.98, 159.45, 162.33, 163.97 (see Figure S13 in the Supplementary Materials); ATR-FTIR: ν_max_ (cm^−1^) 3204, 2990, 2231 (CN), 1651, 1616, 1464, 1281, 1241, 1052, 999 (see Figure S14 in the Supplementary Materials).

#### 4.2.2. General Procedure for the Preparation of oxazolo[5,4-d]pyrimidines (**3a–j**)

To compound **2** (0.95 mmol for synthesis of compounds **3a**, **3c**–**d**, **3f**, and **3h**–**j**, or 0.63 mmol for synthesis of compounds **3b**, **3e**, and **3g**), a solution of the appropriate amine cooled to 5–10 °C (1.5 mL of a 33% solution in water for synthesis of compound **3a**, 1 mL of a 30% solution in ethanol for synthesis of compounds **3b**, **3e**, and **3g**, or 1.5 mL of a 30% solution in ethanol for synthesis of compounds **3c**–**d**, **3f**, and **3h**–**j**) was added in one portion. The reaction mixture was stirred and maintained at a temperature of 5–10 °C for 4 h, and then at room temperature for 20 h. The completion of the reaction was confirmed with TLC, and the formed precipitate was filtered off and dried. The crude product was purified by crystallization from ethanol, and white crystals of the final oxazolo[5,4-*d*]pyrimidines were obtained.

**3a**: 2-(5-Amino-3-methylisoxazol-4-yl)-7-*N*-methylaminooxazolo[5,4-*d*]pyrimidine. Yield 0.093 g, 40%; m.p. 240–241 °C (dec.); HR-ESI-MS: m/z[u/e] calculated for formula C_10_H_11_N_6_O_2_ [M+H]^+^ 247.0938, found 247.0950 (see Figure S2, Table S3 and Table S4 in the Supplementary Materials); ^1^H-NMR (DMSO-*d_6_*, 300. 15 MHz): δ (ppm) 2.34 (s, 3H, the methyl group of the isoxazole moiety), 3.45 (s, 3H, *N*-methyl group), 7.27 (br s, 1H, –NH), 7.90 (s, 2H, –NH_2_), 8.13 (s, 1H, proton at position 5 of the oxazolopyrimidine system) (see Figure S15 in the Supplementary Materials); ^13^C-NMR (DMSO-*d_6_*, 75.47 MHz): δ (ppm) 11.4, 35.0, 81.6, 118.8, 149.0, 153.2, 154.3, 155.9, 157.3, 169.4 (see Figure S16 in the Supplementary Materials); ATR-FTIR: ν_max_ (cm^−1^) 3397, 3294, 1652, 1628, 1600, 1568, 1442, 1323, 1271, 1040 (see Figure S17 in the Supplementary Materials).

**3b**: 2-(5-Amino-3-methylisoxazol-4-yl)-7-*N*-ethylaminooxazolo[5,4-*d*]pyrimidine. Yield 0.056 g, 34%; m.p. 253–254 °C (dec.); HR-ESI-MS: m/z[u/e] calculated for formula C_11_H_13_N_6_O_2_ [M+H]^+^ 261.1096, found 261.1084 (see Figure S3, Table S3 and Table S5 in the Supplementary Materials); ^1^H-NMR (DMSO-*d_6_*, 300 MHz): δ (ppm) 1.27 (t, ^3^*J* = 6.83 Hz, 3H, the methyl group of the ethyl chain), 2.34 (s, 3H, the methyl group of the isoxazole moiety), 4.04 (q, ^3^*J* = 6.70 Hz, 2H, *N*-methylene group of the ethyl chain), 7.24 (s, 1H, –NH), 7.90 (s, 2H, –NH_2_), 8.12 (s, 1H, proton at position 5 of the oxazolopyrimidine system) (see Figure S18 in the Supplementary Materials); ^13^C-NMR (DMSO-*d_6_*, 75.5 MHz): δ (ppm) 11.5, 14.1, 42.2, 81.7, 119.1, 148.7, 152.5, 154.4, 155.8, 157.4, 169.5 (see Figure S19 in the Supplementary Materials); ATR-FTIR: ν_max_ (cm^−1^) 3359, 3280, 1662, 1624, 1578, 1539,1382, 1348, 1286, 1183, 1046 (see Figure S20 in the Supplementary Materials).

**3c**: 2-(5-Amino-3-methylisoxazol-4-yl)-7-*N*-propylaminooxazolo[5,4-*d*]pyrimidine. Yield 0.092 g, 36%; m.p. 220–221 °C (dec.); HR-ESI-MS: m/z[u/e] calculated for formula C_12_H_14_N_6_O_2_ [M+H]^+^ 275.1251, found 275.1262 (see Figure S4, Table S3 and Table S6 in the Supplementary Materials); ^1^H-NMR (DMSO-*d_6_*, 300.15 MHz): δ (ppm) 0.88 (t, ^3^*J_32_* = 7.3 Hz, 3H, terminal-chain methyl), 1.73 (m, ^3^*J_23_* = 7.3 Hz, ^3^*J_21_* = 7.8 Hz, 2H, middle-chain methylene), 2.34 (s, 3H, methyl group of isoxazole moiety), 3.97 (t, ^3^*J_12_* = 7.8 Hz, 2H, chain methylene connected with amine nitrogen at the 7th position), 7.25 (s, 1H, –NH), 7.91 (s, 2H, –NH_2_), 8.12 (s, 1H, proton at position 5 of the oxazolopyrimidine system) (see Figure S21 in the Supplementary Materials); ^13^C-NMR (DMSO-*d_6_*, 75.5 MHz): δ (ppm) (see Figure S22 in the Supplementary Materials); ATR-FTIR: ν_max_ (cm^−1^) 3377, 2873, 2753, 1657, 1606, 1576, 1430, 1377, 1285, 1181, 1041 (see Figure S23 in the Supplementary Materials).

**3d**: 2-(5-Amino-3-methylisoxazol-4-yl)-7-*N*-butylaminooxazolo[5,4-*d*]pyrimidine. Yield 0.060 g, 22%; m.p. 239–240 °C (dec.); HR-ESI-MS: m/z[u/e] calculated for formula C_13_H_17_N_6_O_2_ [M+H]^+^ 289.1408, found 289.1398 (see Figure S5, Table S3 and Table S7 in the Supplementary Materials); ^1^H-NMR (DMSO-*d_6_*, 300.15 MHz): δ (ppm) 0.91 (t, ^3^*J_43_* = 7.3 Hz, 3H, terminal-chain methyl), 1.31 (m-resultant apparent sextet, ^3^*J* ≈ 7.4 Hz, resultant average coupling constant; 2H, third middle-chain methylene coupled with the terminal methyl and the second methylene group of the aliphatic chain), 1.69 (m-resultant apparent pentet, ^3^*J* ≈ 7.4 Hz, resultant average coupling constant; 2H, second middle-chain methylene coupled with *N*-methylene and the third methylene group of the aliphatic chain), 2.43 (s, 3H, methyl group of the isoxazole moiety), 4.01 (t, ^3^*J_12_* = 7.2 Hz, 2H, chain *N*-methylene connected with amine nitrogen at position 7), 7.27 (low br s, 1H, –NH), 7.90 (s, 2H, –NH_2_), 8.12 (s, 1H, proton at position 5 of the oxazolopyrimidine system) (see Figure S24 in the Supplementary Materials); ^13^C-NMR (DMSO-*d_6_*, 75.5 MHz): δ (ppm) 11.4, 13.6, 19.2, 29.8, 46.6, 81.6, 119.0, 148.9, 152.6, 154.3, 155.7, 157.3 (see Figure S25 in the Supplementary Materials); ATR-FTIR: ν_max_ (cm^−1^) 3394, 3110, 2958, 2871, 1659, 1611, 1581, 1542, 1379, 1282, 1180, 1042 (see Figure S26 in the Supplementary Materials).

**3e**: 2-(5-Amino-3-methylisoxazol-4-yl)-7-*N*-pentylaminooxazolo[5,4-*d*]pyrimidine. Yield 0.099 g, 52%; m.p. 237–238 °C (dec.); HR-ESI-MS: m/z[u/e] calculated for formula C_14_H_19_N_6_O_2_ [M+H]^+^ 303.1564, found 303.1549 (see Figure S6, Table S3 and Table S8 in the Supplementary Materials); ^1^H-NMR (DMSO-*d_6_*, 300.15 MHz): δ (ppm) 0.87 (t, ^5^*J* ≈ 6.8 Hz, 3H, terminal methyl group of the pentyl chain), 1.29 (m, 4H, two methylene groups of the pentyl chain), 1.71 (m-resultant apparent pentet, ^3^*J* ≈ 7.0 Hz, resultant average coupling constant; 2H, second middle-chain methylene coupled with *N*-methylene and the third methylene group of aliphatic chain), 2.34 (s, 3H, methyl group of the isoxazole moiety), 4.00 (t, ^3^*J* ≈ 7.2 Hz, 2H, the methylene group of the pentyl chain connected with amine nitrogen at position 7), 7.25 (s, 1H, –NH), 7.91 (brs, 2H, –NH_2_), 8.13 (s, 1H, proton at position 5 of the oxazolopyrimidine system) (see Figure S27 in the Supplementary Materials); ^13^C-NMR (DMSO-*d_6_*, 75.5 MHz): δ (ppm) 11.4, 13.8, 21.8, 27.4, 28.0, 47.0, 81.6, 119.0, 148.9, 152.4, 154.5, 155.9, 157.3, 169.5 (see Figure S28 in the Supplementary Materials); ATR-FTIR: ν_max_ (cm^−1^) 3400, 3112, 2959, 1699, 1616, 1545, 1438, 1381, 1305, 1286, 1183, 1044 (see Figure S29 in the Supplementary Materials).

**3f**: 2-(5-Amino-3-methylisoxazol-4-yl)-7-*N*-[2-*(N,N*-dimethylamino)ethyl]aminooxazolo[5,4-*d*]pyrimidine. Yield 0.150 g, 52%; m.p. 248–249 °C (dec.); HR-ESI-MS: m/z[u/e] calculated for formula C_13_H_18_N_7_O_2_ [M+H]^+^ 304.1516, found 304.1527 (see Figure S7, Table S3 and Table S9 in the Supplementary Materials); ^1^H-NMR (DMSO-*d_6_*, 300.15 MHz): δ (ppm) 2.18 (s, 6H, dimethylamino group), 2.35 (s, 3H, methyl group of the isoxazole moiety), 2.55 (t, triplet partly covered with solvent, ^3^*J_21_* ≈ 5.9 Hz, 2H, methylene of the ethyl chain directly connected to the nitrogen of the dimethylamino group), 4.10 (t, ^3^*J_12_* = 5.9 Hz, 2H, methylene of the ethyl chain directly connected to the amine nitrogen at position 7 of the oxazolopyrimidine system), 7.25 (s, 1H, –NH), 7.90 (s, 2H, –NH_2_), 8.01 (s, 1H, proton at position 5 of the oxazolopyrimidine system) (see Figure S30 in the Supplementary Materials); ^13^C-NMR (DMSO-*d_6_*, 75.5 MHz): δ (ppm) 11.4, 44.3, 45.3, 56.2, 81.6, 118.9, 149.3, 152.6, 154.2, 155.7, 157.3, 169.4 (see Figure S31 in the Supplementary Materials); ATR-FTIR: ν_max_ (cm^−1^) 3311, 3209, 2840, 1663, 18609, 1580, 1537, 1380, 1370, 1285, 1193, 1157, 1056, 1026 (see Figure S32 in the Supplementary Materials).

**3g**: 2-(5-Amino-3-methylisoxazol-4-yl)-7-*N*-[3-(*N,N*-dimethylamino)propyl]aminooxazolo[5,4-*d*]pyrimidine. Yield 0.039 g, 20%; m.p. 244–245 °C (dec.); HR-ESI-MS: m/z[u/e] calculated for formula C_14_H_20_N_7_O_2_ [M+H]^+^ 318.1673, found 318.1677 (see Figure S8, Table S3 and Table S10 in the Supplementary Materials); ^1^H-NMR (DMSO-*d_6_*, 300.15 MHz): δ (ppm) 1.85 (m-resultant apparent pentet, ^3^*J_23_* ≈ 6.8 Hz, ^3^*J_21_* ≈ 7.0 Hz, 2H, middle methylene group of the propyl chain), 2.12 (s, 6H, two methyls of the dimethylamino group), 2.22 (t, ^3^*J_32_* = 6.8 Hz, 2H, methylene of the propyl chain directly connected to nitrogen of the dimethylamino group), 2.35 (s, 3H, methyl group of the isoxazole moiety), 4.02 (t, ^3^*J_12_* = 7.0 Hz, 2H, methylene of the propyl chain directly connected to the amine nitrogen at position 7 of the oxazolopyrimidine system), 7.26 (low br s, 1H, -NH), 7.90 (s, 2H, –NH_2_), 8.08 (s, 1H, proton at position 5 of the oxazolopyrimidine system) (see Figure S33 in the Supplementary Materials); ^13^C-NMR (DMSO-*d_6_*, 75.5 MHz): δ (ppm) 11.4, 25.1, 45.0, 45.3, 55.8, 81.6, 119.0, 149.0, 152.5, 154.3, 155.7, 157.2, 169.4 (see Figure S34 in the Supplementary Materials); ATR-FTIR: ν_max_ (cm^−1^) 3334,2958, 2861, 2836, 1652, 1599, 1577, 1532, 1422, 1371, 1344, 1261, 1160, 1021, 1010, 800 (see Figure S35 in the Supplementary Materials).

**3h**: 2-(5-Amino-3-methylisoxazol-4-yl)-7-*N*-(2-hydroxyethyl)aminooxazolo[5,4-*d*]pyrimidine. Yield 0.101 g, 39%; m.p. 213–214 °C (dec.); HR-ESI-MS: m/z[u/e] calculated for formula C_11_H_12_N_6_O_3_ [M+H]^+^ 277.1044, found 277.1053 (see Figure S9, Table S3 and Table S11 in the Supplementary Materials); ^1^H-NMR (DMSO-*d_6_*, 300.15 MHz): δ (ppm) 2.35 (s, 3H, methyl group of the isoxazole moiety), 3.68 (t, ^3^*J_1,2_* = 4.9 Hz, 2H, methylene group connected with the amine nitrogen at position 7), 4.08 (t, ^3^*J_21_* = 5.0 Hz, 2H, methylene group connected hydroxyl group), 5.00 (low br s, 1H, -OH), 7.25 (low br s, 1H, –NH), 7.91 (s, 2H, -NH_2_), 7.98 (s, 1H, proton at position 5 of the oxazolopyrimidine system) (see Figure S36 in the Supplementary Materials); ^13^C-NMR (DMSO-*d_6_*, 75.5 MHz): δ (ppm) 11.4, 49.5, 57.4, 81.6, 118.9, 149.5, 152.8, 154.2, 155.8, 157.2, 169.4 (see Figure S37 in the Supplementary Materials); ATR-FTIR: ν_max_ (cm^−1^) 3223, 3126, 1642, 1610, 1578, 1543, 1430, 1374, 1348, 1160, 1040 (see Figure S38 in the Supplementary Materials).

**3i**: 2-(5-Amino-3-methylisoxazol-4-yl)-7-*N*-(3-hydroxypropyl)aminooxazolo[5,4-*d*]pyrimidine. Yield 0.086 g, 31%; m.p. 225–226 °C (dec.); HR-ESI-MS: m/z[u/e] calculated for formula C_12_H_15_N_6_O_3_ [M+H]^+^ 291.1200, found 291.1214 (see Figure S10, Table S3 and Table S12 in the Supplementary Materials); ^1^H-NMR (DMSO-*d_6_*, 300.15 MHz): δ (ppm) 1.85 (m-resultant apparent pentet, ^3^*J* ≈ 6.2 Hz, resultant average coupling constant; 2H, middle methylene group of the propyl chain), 2.34 (s, 3H, methyl group of the isoxazole moiety), 3.41 (t, ^3^*J*_12_ = 5.9 Hz, 2H, methylene group connected with amine nitrogen at position 7 of the oxazolopyrimidine system), 4.09 (t, ^3^*J_32_* = 6.6 Hz, 2H, methylene group connected with a hydroxyl group), 4.88 (low br s, 1H, –OH), 7.26 (low br s, 1H, –NH), 7.88 (b rs, 2H, –NH_2_), 8.09 (s, 1H, proton at position 5 of the oxazolopyrimidine system) (see Figure S39 in the Supplementary Materials); ^13^C-NMR (DMSO-*d_6_*, 75.5 MHz): δ (ppm) 11.4, 31.0, 44.1, 57.3, 81.6, 119.0, 149.0, 153.0, 154.4, 155.8, 157.3, 169.5 (see Figure S40 in the Supplementary Materials); ATR-FTIR: ν_max_ (cm^−1^) 3321, 3130, 1652, 1614, 1585, 1542, 1349, 1270, 1167, 1061, 1046 (see Figure S41 in the Supplementary Materials).

**3j**: 2-(5-Amino-3-methylisoxazol-4-yl)-7-*N*-[2-(morpholin-4-yl)ethyl]aminooxazolo[5,4-*d*]pyrimidine. Yield 0.126 g, 38%; m.p. 225–226 °C (dec.); HR-ESI-MS: m/z[u/e] calculated for formula C_15_H_20_N_7_O_3_ [M+H]^+^ 346.1622, found 346.1639 (see Figure S11, Table S3 and Table S13 in the Supplementary Materials); ^1^H-NMR (DMSO-*d_6_*, 300.15 MHz): δ (ppm) 2.35 (s, 3H, methyl group of the isoxazole moiety), 2.43 (m—visible as a distorted apparent triplet, 4H, two morpholine ring methylene groups connected directly with the morpholine ring nitrogen), 2.61 (t, ^3^*J_21_* ≈ 5.7 Hz, 2H, methylene group of the ethyl chain directly connected with the morpholine ring nitrogen), 3.53 (m—visible as an apparent triplet, ^3^*J ≈* 4.1 Hz, 4H, two morpholine ring methylene groups connected directly with the morpholine ring oxygen), 4.12 (t, ^3^*J_12_* ≈ 5.7 Hz, 2H, methylene group of the ethyl chain directly connected with the amine nitrogen at position 7 of the oxazolopyrimidine system), 7.26 (brs, 1H, –NH), 7.90 (brs, 2H, –NH_2_), 8.04 (s, 1H, proton at position 5 of the oxazolopyrimidine system) (see Figure S42 in the Supplementary Materials); ^13^C-NMR (DMSO-*d_6_*, 75.5 MHz): δ (ppm) 11.4, 43.4, 53.3, 55.2, 66.3, 81.6, 118.9, 149.4, 152.7, 154.2, 155.7, 157.3, 169.4 (see Figure S43 in the Supplementary Materials); ATR-FTIR: ν_max_ (cm^−1^) 3348, 3280, 2835, 1645, 1608, 1581, 1537, 1428, 1374, 1281, 1180, 1113, 1066, 1032, 1009, 866 (see Figure S44 in the Supplementary Materials).

### 4.3. Biology

#### 4.3.1. Cell Lines and Conditions

All cell lines were grown in 5% CO_2_ at 95% humidity and 37 °C, with both morphology and confluence assessed twice per week. When the confluence was greater than 70%, adherent cell lines were detached from the bottle surface with a TrypLE solution and then used in assays or reduced. The cells were collected into centrifuge tubes and centrifuged at 1000× *g* for 5 min at room temperature. The supernatant was removed to dry. The cell pellets were then resuspended in an appropriate medium. The cells were counted using a Bürker chamber and resuspended in 96-well plates to a cell density of 10,000 cells per well for MTT assay. For the rhodamine accumulation test and the detection of apoptosis assay, cells were seeded at a density of 30,000 per well. In the migration assay, cells were seeded at a density of 20,000 per well, and monolayer formation was awaited.

In this study, normal human dermal fibroblasts (NHDFs), breast cancer cells (MCF7), human lung adenocarcinoma cells (A549), murine fibroblasts (L929), colon adenocarcinoma cells (HT29), and colorectal adenocarcinoma cells (LoVo) were used. All cell lines were cultured in the recommended media. NHDF cells (CC-2511, Lonza, Basel, Switzerland) were cultured in DMEM without phenol red. The human lung adenocarcinoma cell line A549 and breast cancer cell line MCF-7 were obtained from the ECACC. These cell lines were cultured in Eagle’s minimal essential medium (MEM). Murine fibroblast L929 (Sigma-Aldrich, Merck Group, Headquarters, Darmstadt, Germany; the European Collection of Authenticated Cell Cultures—ECACC) cells were grown in DMEM. The colon adenocarcinoma cell line HT29 (ATCC) was grown in RPMI 1640, and LoVo cells were grown in DMEM F12. All media were supplemented with 10% FBS and 100 µg/mL of penicillin–streptomycin.

#### 4.3.2. Preparation of Solutions of the Tested Compounds

All compounds were dissolved in DMSO and the reference drugs were dissolved in physiological saline to concentrations of 10 mM. Concentrations from 1 to 500 μM in the media recommended for the cell lines were prepared for the tests.

#### 4.3.3. Viability Assay

All of the synthesized derivatives were evaluated via the MTT assay. All cell lines were seeded into 96-well plates at a density of 10,000 cells per well. To allow the cells to adhere to the surface of the plates, they were incubated overnight before the application of the test compounds. Cells that did not stick to the medium were removed along with the supernatant. The tests were performed for concentrations of 1, 5, 10, 25, 50, 100, 250, and 500 μM. The solutions of the test compounds were removed from the culture on the 96-well plates. The MTT solution had a 1 mg/mL concentration with PBS and was added for 2 h in 5% CO_2_ and 95% humidity at 37 °C. The supernatant was then removed. The violet crystal was dissolved in isopropanol for 30 min by shaking. Absorbance was measured at 570 nm.

#### 4.3.4. Rh-123 Assay

The Rh-123 assay was conducted to estimate the activity of P-glycoprotein. The cells were plated on sterile, opaque-walled 96-well plates at a volume of 100 µL of culture medium and 20,000 cells per well of the HT29 cell line. The next day, the medium was cleared from the plates with cells, and then the freshly prepared tested compounds were added for 24 h. The rhodamine accumulation test was performed for concentrations of 1, 2, 5, 10, and 20 μM. Before the experiment, the solution of Rh-123 (Sigma) was prepared at a concentration of 10 mM in a 1:1 DMSO–water mixture. Solutions of the test compounds in nutrient broth (without FBS) were added to the test wells at a volume of 100 µL/well. Cells were incubated with the test compounds for 2 h. After this time, Rh-123 was added to the wells to a final concentration of 12.5 µM and incubated for 60 min. After incubation, the supernatant from the pellets was removed. The cell pellets were dissolved (150 µL/well) in 20 mM Tris-HCl at a pH of 7.7 (Sigma-Aldrich) containing 0.2% sodium dodecyl sulfate (SDS) (Pol-Aura) to lyse the cells and release the intracellular fluorescent substrate. Fluorescence was measured at ex: 485 nm/em: 538 nm using a VariuScan microplate reader.

#### 4.3.5. Detection of Apoptosis

HT29 primary colon adenocarcinoma cells were grown in 96-well plates for 24 h. The 5 selected derivatives were added at concentrations of 1, 2, 5, and 10 µM, and then incubated for 24 h. The mixture of Annexin V conjugated with fluorescein and propidium iodide in PBS with Mg^2+^ and Ca^2+^ ions (Thermo Fisher Scientific, Waltham, MA, USA) was then applied. The incubation lasted 20 min at 37 °C. Pictures were taken using a fluorescence microscope. Thirteen images were taken from each well using an EVOS FL fluorescence microscope, with three replications for each compound. The open ImageJ space was used to analyze the number of cells. The Thermo Fisher Dead Cell Apoptosis Kit with Annexin V FITC and Propidium Iodide (PI) detects phosphatidylserine extrusion in apoptotic cells using Annexin V conjugated with a green fluorescent FITC dye and dead cells stained by PI with red fluorescence. After applying the dyes, apoptotic cells showed only green fluorescence, late-apoptotic cells showed green and red fluorescence simultaneously, and necrotic cells showed only red fluorescence. Live cells showed neither green nor red fluorescence.

#### 4.3.6. Migration Assay

After seeding, HT29 cells were incubated until they formed a monolayer over the entire surface of the well. A scratch was made on the surface overgrown with cells, and then photomicrographs of the scratch were taken using the EVOS FL microscope. The freshly prepared compound **3g** and the reference drugs were added at concentrations of 10, 25, 50, and 100 µM. Then, they were incubated for 24 h, and micrographs were performed at that time using the EVOS FL microscope. The scratch width was measured using the ImageJ open-source platform. The measurement of scratch width was also performed after 24 h without adding compounds.

#### 4.3.7. ELISA

##### Human Caspase-3 (Active) ELISA Kit (KHO1091)

The test was performed on an HT29 colon adenocarcinoma cell line lysate treated with compound **3g**. Cells from the HT29 line were harvested by centrifugation (non-adherent cells) and scraping from the culture flasks (adherent cells), and then the cells were washed twice with cold PBS. The supernatant was removed and discarded, and cell pellets were collected. The cell pellets were then lysed in a cell extraction container for 30 min on ice and centrifuged every 10 min. The lysate was transferred to microcentrifuge tubes and centrifuged at 13,000 rpm for 10 min at 4 °C. The supernatant was transferred to clean microcentrifuge tubes. Caspase-3 standard (active) was prepared from recombinant protein. The caspase-3 standard was reconstituted with the standard solvent solution at a concentration of 2.5 ng/mL. The contents were swirled and then left for 10 min to ensure complete dissolution. Labeled at 2.5 ng/mL, human caspase was used typically within 1 h of dissolution. Then, 250 μL of standard diluent buffer was added to each of the 7 tubes labeled as follows: 1.25, 0.625, 0.313, 0.156, 0.078, 0.039, and 0 ng/mL human caspase-3. Serial dilutions of the standard were performed. The standards, controls, and samples were added (100 µL) to the appropriate dimples.

In this method, a monoclonal capture antibody specific to caspase-3 (cleaved) was coated onto the wells of a 96-well plate. During the first incubation, standards of known contents and unknown samples were pipetted into the wells, and the antigen was bound to the immobilized (capture) antibody. The plate was covered with a lid and incubated for 2 h at room temperature. The wells were washed 4 times with 1× wash buffer antigen that was bound by adding 100 µL of a caspase-3-detecting antibody solution to each well except for the chromogen blanks. After washing, an antibody specific to the caspase-3 protein was added to the wells and served as a detection antibody by binding to the immobilized protein captured during the first incubation. The plate was covered with a lid and incubated for 1 h at room temperature. After washing, anti-rabbit IgG labeled with horseradish peroxidase was added. This was bound to the detection antibody. The wells were washed 4 times with 1× wash buffer and, as a detection antibody, 100 µL of 1× anti-rabbit IgG HRP solution was added to each well except for the chromogen blanks. The plate was covered with a lid and incubated for 30 min at room temperature. After the third incubation and washing to remove all of the unbound enzymes, a substrate solution (TMB) was added, which was acted upon by the bound enzyme to produce color. The substrate solution began to turn blue. Then, it was incubated for 30 min at room temperature in the dark. Next, 100 µL of stop solution was added to the stabilized chromogen for each well. The solution in the wells turned from blue to yellow. The absorbance at 450 nm was read for 2 h after the stop solution was added. Curve software was used to generate a standard curve. Concentrations for unknown samples and controls were read from the standard curve. The intensity of the colored product was directly proportional to the concentration of the target protein present in the original specimen, and the optical density was read on a microplate reader.

##### p53 Human ELISA Kit (BMS256)

The assay was performed on a lysate of the HT29 colon adenocarcinoma cell line treated with compound **3g**. The microwells of the 96-well plate were washed 2 times with approximately 400 µL of washing buffer per well. The washing buffer was left in the wells for approximately 15 s prior to aspiration. Care was taken not to scratch the surface of the microwells. After the final rinsing step, the wells were emptied. An absorbent swab was used to remove excess wash buffer. Standard dilutions were applied to the microwell plate. Two rows of human p53 standard were prepared at dilutions ranging from 50.00 to 0.78 U/mL. Human p53, which was present in the sample and the standard, bound to the antibodies adsorbed in the microwells. The biotin-conjugated anti-human p53 antibody bound to human p53 was captured by the first antibody. Then, 50 μL of the prepared biotin conjugate was added to all wells. The plate was covered with adhesive film and incubated at room temperature (18 to 25 °C) for 2 h on a microplate shaker. Unbound p53 biotin-conjugated anti-human antibody was removed after incubation during the washing step. When streptavidin–HRP was added, it bound to the biotin-conjugated anti-human p53 antibody. Streptavidin–HRP was prepared. The adhesive film was removed and the wells were washed 3 times. Then, 100 μL of diluted streptavidin–HRP was added to all wells, covered with adhesive foil, and incubated at room temperature (18 to 25 °C) for 1 h on a microplate shaker. The adhesive foil was removed and the microwells were washed 3 times. After incubation, unbound streptavidin–HRP was removed during the washing step, and 100 µL of HRP-reactive TMB substrate solution was added to the wells. It was then incubated at room temperature (18 to 25 °C) for 10 min. The color change was monitored with an ELISA measured at 620 nm. The colored product was formed in proportion to the quantity of human p53 protein present in the sample and the standard. The substrate reaction was stopped as soon as Standard 1 (highest concentration) had an OD of 0.9–0.95. The standard with the highest concentration turned dark blue. The enzyme reaction was stopped by adding 100 µL of stop solution to each well. The results were read immediately after the addition of the enzyme stop solution. Absorbance was measured with a spectrophotometer at 450 nm. A standard curve was prepared from 7 dilutions of the human p53 standard from which the values for compound **3g** were read.

##### BCL-2 Human ELISA Kit (BMS244-3)

The assay was performed on a lysate of the HT29 colon adenocarcinoma cell line treated with compound **3g**. The wells of the assay microplate were coated with a BCL-2-specific antibody, and the BCL-2 present in the test and standards was bound to the antibodies adsorbed in the microwells, which were washed twice with 400 µL of washing buffer per well with careful aspiration. The washing buffer was left in the wells for 15 s before aspiration. Care was taken not to scratch the surface of the microwells. Following the final washing step, the wells were emptied and excess washing buffer was discarded. Two rows of the human standard BCL-2 were prepared at dilutions ranging from 32.0 to 0.5 ng/mL. Biotin conjugate was prepared, and 50 µL of biotin conjugate was added to all wells, covered with adhesive foil, and incubated at room temperature (18 to 25 °C) for 2 h on a microplate shaker. The adhesive film was removed and washed 3 times. The unbound anti-human BCL-2 antibody conjugated to biotin after incubation was removed during the washing step. The second detector antibody—streptavidin–HRP—was added to all wells, covered with cling film, and incubated at room temperature (18 to 25 °C) for 1 h on a microplate shaker, and then the microwells were washed 3 times. TMB substrate solution (100 μL), which reacts with the enzyme–antibody–BCL-2 complex, was added to all of the wells to generate a measurable signal. The incubation was at room temperature (18 to 25 °C) for 10 min. Direct exposure to intense light was avoided. The color change of the mixture was monitored with an ELISA reader at 620 nm. The substrate reaction was stopped by adding 100 µL of the melt solution to each well as soon as Standard 1 had an OD of 0.9–0.95. The results were read immediately after the stop solution was added. The reading was taken at the original wavelength of 450 nm. A standard curve was prepared from 7 dilutions of the human standard BCL-2. The signal intensity was directly proportional to the concentration of BCL-2 present in the tested sample.

#### 4.3.8. Statistical Analysis

Biological assays were performed in three independent replicates, and each replicate used four samples. Three repetitions were made for the ELISA determinations. For the assessment of cytotoxicity, mathematical models were developed using the GraphPad PRISM software, based on which CC_50_ was determined. The values of CC_50_ were estimated based on nonlinear regression using the dependence of the biological effect on the molar concentrations of the compounds (four-parameter logistic model with Hill slope). The CC_50_ is the concentration that inhibits 50% of cell viability. The CC_50_ values that were determined are summarized in Table 1.

Other results (such as P-glycoprotein-inhibitory activity, pro-apoptotic activity, effect on cell migration, and effects on the levels of p53, caspase-3, and BCL-2) are presented as the mean ± SD (standard deviation). P-glycoprotein-inhibitory activity was comparable to that of the corresponding control (E/E_0_), where E is the result for the measured sample and E_0_ is the result for the control. The control was a cell culture incubated only with the appropriate medium, without the tested derivatives. The statistical evaluation was performed using the Statistica v13.3 and GraphPad PRISM software. All graphs were created in Microsoft Excel. The data had a normal distribution and equality of variance, so a one-way ANOVA with Tukey’s post hoc test was performed. The level of significance was set at *p* < 0.05.

### 4.4. Molecular Modeling

The geometries of compounds **3a**, **3e–g**, **3j**, and tivozanib were optimized using the B3LYP/6-31+G** level of theory using the Gaussian 09 package [84]. In order to take into account the solvent effect (water solution), the polarizable continuum model (PCM) was used [85]. The standard protocol of the AutoDock 4.2 package was used during molecular docking to predict the binding mode and the free energy of binding [86]. The protein crystal structure of vascular endothelial growth factor receptor-2 (VEGFR-2) (PDB ID:4ASE) was downloaded from the Protein Data Bank (PDB) [87]. The validation of docking was performed by docking tivozanib into the crystal structures of VEGFR-2 and comparing its position in the original crystallographic structure. The root-mean-square deviation (RMSD) was calculated to measure the docking prediction accuracy on the LigRMSD web server [88]. The position was optimal when its RMSD was found to be less than 1.5 Å. The protein and ligand preparation and the docking procedure were described in detail in previous studies [60,61]. The data were visualized using a Chimera and a BIOVIA Discovery Studio visualizer [89,90]. Physicochemical properties, pharmacokinetics, and ADME activity were investigated in silico based on database function integrated with ADMETlab2.0 [62].

## Figures and Tables

**Table 1:** Cytotoxic activity of compounds **3a–j** and reference drugs against the tested cell lines determined in the MTT assay and expressed as the cytotoxic concentration (**CC_50_ ± SD**) that causes a 50% reduction in viable cells. **SD** is the standard deviation. **SI** is the selectivity index, which is the ratio of the cytotoxic concentration (**CC_50_**) against healthy cells (**NHDFs**) to the cytotoxic concentration against cancer cells. **N/A** (i.e., not assessed) means that CC_50_ could not be correctly determined in these cases.

Compound	L929	NHDF	A549		MCF7		LoVo		HT29	
	CC_50_ ± SD ^a^ (µM)	CC_50_ ± SD ^a^ (µM)	CC_50_ ± SD ^a^ (µM)	SI	CC_50_ ± SD ^a^ (µM)	SI	CC_50_ ± SD ^a^ (µM)	SI	CC_50_ ± SD ^a^ (µM)	SI
**3a**	Non-toxic ^b^	Non-toxic	245.15 ± 19.02	>>1 ^c^	N/A	-	N/A	-	158.76 ± 12.07	>>1
**3b**	235.08 ± 11.54	Non-toxic	N/A	-	192.79 ± 16.90	>>1	N/A	-	194.20 ± 15.36	>>1
**3c**	90.64 ± 8.75	171.81 ± 14.47	N/A	-	199.26 ± 16.06	0.862	N/A	-	224.32 ± 23.05	0.766
**3d**	Non-toxic	126.17 ± 9.87	178.95 ± 14.85	0.705	133.63 ± 14.03	0.944	226.05 ± 8.28	0.558	153.79 ± 13.38	0.820
**3e**	89.20 ± 6.04	124.65 ± 11.46	124.77 ± 12.05	0.999	155.03 ± 15.54	0.804	**177.52 ± 6.65**	0.702	**129.41 ± 10.04**	0.963
**3f**	207.49 ± 16.47	171.62 ± 20.70	147.81 ± 13.80	1.161	194.73 ± 11.97	0.881	235.26 ± 13.02	0.729	152.69 ± 15.02	1.124
**3g**	83.67 ± 4.42	155.58 ± 15.49	138.50 ± 12.86	1.123	208.65 ± 9.67	-	223.93 ± 17.09	0.695	**58.44 ± 8.75**	**2.662**
**3h**	145.75 ± 11.11	Non-toxic	N/A	-	N/A	-	N/A	-	N/A	-
**3i**	171.36 ± 22.77	Non-toxic	N/A	-	251.20 ± 22.05	>>1	N/A	-	N/A	-
**3j**	52.85 ± 9.51	Non-toxic	N/A	-	197.49 ± 13.08	>>1	N/A	-	**99.87 ± 10.90**	**>>1**
**Cisplatin**	30.05 ± 5.07	11.49 ± 3.02	8.17 ± 2.39	1.406	29.72 ± 5.25	0.387	27.39 ± 8.01	0.419	47.17 ± 7.43	0.244
**5-FU**	38.15 ± 7.13	20.37 ± 2.97	269.45 ± 17.15	0.076	217.47 ± 20.01	0.094	72.20 ± 14.43	0.282	381.16 ± 25.51	0.053

^a^ The values of CC_50_ were estimated based on nonlinear regression using the dependence of the biological effects on the molar concentrations of the compounds (four-parameter logistic model with Hill slope). The solutions of the compounds were prepared at 8 different concentrations (i.e., 1, 5, 10, 25, 50, 100, 250, and 500 μM). Four samples of each concentration were taken. The entire test was performed three times independently. Homogeneity of variances was checked with Levene’s and Brown–Forsythe tests. To avoid type I error, the statistical significance of differences in the mean CC_50_ values of all of the compounds within a single cell line and in the mean CC_50_ values of each individual compound for cancerous cell lines and healthy reference cells was tested with conservative Tukey’s HSD and Bonferroni post hoc tests for multiple comparisons. Moreover, the statistical significance of the differences in the mean CC_50_ values of the tested compounds in relation to the mean CC_50_ values of the reference drugs was also tested with Dunnett’s post hoc test. The threshold for significance was set at α = 0.05; hence, the differences in the means with a *p*-value (probability value) < 0.05 (i.e., for confidence level > 95%) were considered statistically significant. ^b^ No toxicity was observed in the tested concentration range of 1–500 µM. ^c^ >> 1 means a very high value due to no cytotoxic effect of the tested compound against the healthy NHDF cells.

**Table 2:** The changes in the expression of selected signaling molecules induced by **3g** in cultures of the colon adenocarcinoma cell line (HT29).

Signaling Molecules	Compound 3g	Standard Deviation (SD)
BCL-2	0.41 [pg/mL]	±0.11
Caspase-3	12.27 [ng/mL]	±3.03
p53	4.22 [U/mL]	±0.97

**Table 3:** The best docking conformations rated by scoring functions, including in terms of free energy of binding, inhibition constant, and intermolecular interaction observed during molecular docking to the active site of VEGFR-2.

	Compound 3a	Compound 3e	Compound 3f	Compound 3g	Compound 3j	Tivozanib
**∆G_binding_ (kJ/mol)**	−38.5	−39.3	−40.0	−41.0	−47.3	−48.1
**K_i_ (μM)**	0.16	0.11	0.09	0.06	0.04	0.02
**Conventional HB**	Glu917 Cys919	Glu917 Cys919	Glu917	Glu917 Cys919	Glu917 Cys919	Glu885 Cys919
**Carbon HB**	Ala866 Val914 Asp1046	-	Ala866 Val914	Glu885 Asp1046	Glu885 Val914 Cys1045	Leu840 Glu917 Cys919 Lys920
**π–π stacking**	Phe918	Phe918 Phe1047	Phe918 Phe1047	Phe918 Phe1047	Phe918	Phe918
**Alkyl, π–alkyl**	Leu840 Val848 Ala866 Lys868 Val916 Cys919 Leu1035 Cys1045 Phe1047	Leu840 Val848 Ala866 Lys868 Val914 Val916 Cys919 Phe1047	Leu840 Val848 Ala866 Leu889 Val916 Cys919 Phe1047	Leu840 Val848 Ala866 Lys868 Leu889 Val899 Val916 Cys919 Phe1047	Leu840 Ala866 Lys868 Leu1035 Phe1047	Val848 Ala866 Lys868 Leu889 Ile892 Val916 Phe918 Leu1035 Cys1045 Phe1047
**π–σ**	Leu1035	Leu1035	Leu1035	Val848 Leu1035	Val848 Leu1035	Leu889
**Van der Waals**	Val867 Glu885 Val899	Val867 Glu885 Leu889 Val899 Cys1045 Asp1046 Ala1050	Val867 Lys868 Glu885 Leu889 Ile915 Asp1046	Val914 Cys1045	Val867 Leu889 Val899 Val916 Gly922 Cys1045	Ile888 Val899 Gly992 Asp1046
**π–Sulfur**	-	-	Cys1045	-	-	-

**Table 4:** Cytotoxic activity (expressed as 50% inhibitory concentration) on the A549, MCF7, LoVo, and HT29 tumor cell lines, collected from the literature, of a few drugs that are confirmed inhibitors of the VEGFR-2 receptor, among others.

Drug Name	IC_50_ (µM)			
	A549	MCF7	LoVo	HT29
**Tivozanib**	0.19 ± 0.04 ^a^	0.25 ± 0.03 ^a^	No data	No data
**Regorafenib**	No data	No data	2 ^b^	2.75 ± 0.18 ^c^
**Cabozantinib**	9.52 ± 0.91 ^d^ 15.23 ± 0.6 ^e^	14.26 ± 0.87 ^e^	No data	11.5 ± 1.1 ^d^
**Lenvatinib**	No effect ^f^	No data	No data	431 ^g^
**Axitinib**	4.88 ± 0.83 ^h^	No data	No data	13.12 ± 1.84 ^h^
**Vandetanib**	2.7 ± 0.5 ^i^	12.23 ± 0.28 ^j^	3.5 ± 0.9 ^k^	10 ± 0.4 ^k^
**Pazopanib**	4–6 ^l^	6.29 ^m^	No data	>5 ^n^
**Sunitinib**	6.98 ^o^	7.30 ^o^	4.18 ± 0.27 ^p^	4.89 ^o^ 2.25 ± 0.27 ^p^
**Sorafenib**	5.62 ± 2.13 ^r^	7.26 ± 0.3 ^s^ 2.0 ^t^	31 ^u^	17.28 ± 3.8 ^r^

^a^ Cytotoxic data taken from reference [64], ^b^ cytotoxic data taken from reference [65], ^c^ cytotoxic data taken from reference [66], ^d^ cytotoxic data taken from reference [67], ^e^ cytotoxic data taken from reference [68], ^f^ cytotoxic data taken from reference [69], ^g^ cytotoxic data taken from reference [70], ^h^ cytotoxic data taken from reference [71], ^i^ cytotoxic data taken from reference [72], ^j^ cytotoxic data taken from reference [73], ^k^ cytotoxic data taken from reference [74], ^l^ cytotoxic data taken from reference [75], ^m^ cytotoxic data taken from reference [76], ^n^ cytotoxic data taken from reference [77], ^o^ cytotoxic data taken from reference [78], ^p^ cytotoxic data taken from reference [79], ^r^ cytotoxic data taken from reference [80], ^s^ cytotoxic data taken from reference [81], ^t^ cytotoxic data taken from reference [82], ^u^ cytotoxic data taken from reference [83].

## Data Availability

Not applicable.

## Supplementary Materials

The following supporting information can be downloaded at: https://www.mdpi.com/article/10.3390/ijms231911694/s1. Reference [91] is cited in the supplementary materials.
