# Supplementary material for: New Oxazolo[5,4-d]pyrimidines as Potential Anticancer Agents: Their Design, Synthesis, and In Vitro Biological Activity Research
Source: Int J Mol Sci. 2022 Oct 2;23(19):11694. doi: 10.3390/ijms231911694 (PMC9569971; doi:10.3390/ijms231911694)
Supplement: Supplementary file 1 [file ijms-23-11694-s001.zip › ijms-1867381-supplementary.pdf]

SUPPLEMENTARY MATERIAL

New Oxazolo[5,4-*d*]pyrimidines as Potential Anticancer Agents: Their Design, Synthesis and *In Vitro* Biological Activity Research

Aleksandra Sochacka-Ćwikła [1,*], Marcin Mączyński [1,*], Żaneta Czyżnikowska [2], Benita Wiatrak [3], Izabela Jęśkowiak [3], Albert Czerski [4], and Andrzej Regiec [1]

[1] Department of Organic Chemistry and Drug Technology, Faculty of Pharmacy, Wroclaw Medical University, 211A Borowska Street, 50-556 Wrocław, Poland
[2] Department of Inorganic Chemistry, Faculty of Pharmacy, Wroclaw Medical University, 211A Borowska Street, 50-556 Wrocław, Poland
[3] Department of Pharmacology, Faculty of Medicine, Wroclaw Medical University, 2 Mikulicza-Radeckiego Street, 50-345 Wrocław, Poland
[4] Department of Biostructure and Animal Physiology, Wroclaw University of Environmental and Life Sciences, 25/27 Norwida Street, 50-375 Wrocław, Poland

*****Corresponding authors: aleksandra.sochacka-cwikla@umw.edu.pl (ASC); Tel.: +48 717840634; marcin.maczynski@umw.edu.pl (MM); Tel.: +48 717840342

TABLE OF CONTENTS

CHEMISTRY 3

Electrospray Ionization Mass Spectrometry (ESI-MS) measurements of intermediate (2), and oxazolo[5,4-*d*]pyrimidines (3a-j) 3

Analysis and Visualizations of Electrospray Ionization Mass Spectrometry (ESI-MS) spectra of intermediate 2 3

Analysis and Visualizations of High Resolution Electrospray Ionization Mass Spectrometry (HR-ESI-MS) spectra of the compounds 3a-j 4

Visualizations of Nuclear magnetic resonance (NMR) and Infrared (IR) spectra of intermediate 2 12

Visualizations of Nuclear magnetic resonance (NMR) and Infrared (IR) spectra of the compound 3a 13

Visualizations of Nuclear magnetic resonance (NMR) and Infrared (IR) spectra of the compound 3b 15

Visualizations of Nuclear magnetic resonance (NMR) and Infrared (IR) spectra of the compound 3c 16

Visualizations of Nuclear magnetic resonance (NMR) and Infrared (IR) spectra of the compound 3d 18

Visualizations of Nuclear magnetic resonance (NMR) and Infrared (IR) spectra of the compound 3e 19

Visualizations of Nuclear magnetic resonance (NMR) and Infrared (IR) spectra of the compound 3f 21

Visualizations of Nuclear magnetic resonance (NMR) and Infrared (IR) spectra of the compound 3g 22

Visualizations of Nuclear magnetic resonance (NMR) and Infrared (IR) spectra of the compound 3h 24

Visualizations of Nuclear magnetic resonance (NMR) and Infrared (IR) spectra of the compound 3i 25

Visualizations of Nuclear magnetic resonance (NMR) and Infrared (IR) spectra of the compound 3j 27

BIOLOGY 29

MOLECULAR DOCKING 31

PHYSICOCHEMICAL PROPERTIES, PHARMACOKINETICS AND ADME ACTIVITY 33

CHEMISTRY

Electrospray Ionization Mass Spectrometry (ESI-MS) measurements of intermediate (2), and oxazolo[5,4-*d*]pyrimidines (3a-j)

Electrospray ionization mass spectrometry (ESI-MS) spectra of the all compounds were recorded with the compact^{TM} Electrospray Ionisation-Quadrupole-Time of Flight (ESI-Q-TOF) Mass Spectrometer (Bruker Daltonics) in methanol solutions in positive ionization. The samples of the compounds were dissolved in methanol in eppendorf tubes and immediately measured.

Requirements for the high resolution electrospray ionization mass spectrometry (HR-ESI-MS) measurement:

- mass spectrometer resolution must be above 16000, where the resolution, according to the definition recommended by IUPAC for mass spectrometry [1], is expressed with the following formula:

$$Resolution = \frac{M}{\Delta M}$$

Where M – is the value of ion mass to charge relation, ΔM – is resolving power (mass) that is the width of the peak measured at 50% fraction of the peak height (i.e. the full width at half maximum (FWHM))

- mass measurement relative error must be below |±5| ppm (indicating a good mass measurement accuracy). Relative error, which was expressed in ppm (parts per million), was calculated on the basis of the following formula:

$$RelError[ppm] = \left| \frac{Calculated\ m/z - Found\ m/z}{Calculated\ m/z} \bullet 10^6 \right|$$

Analysis and Visualizations of Electrospray Ionization Mass Spectrometry (ESI-MS) spectra of intermediate 2

Table S1. Comparative set of masses of quasi-parent molecular $[M+H]^+$ ion of intermediate **2**. Relative error associated with mass measurement of the intermediate **2**.

No.	Calculated m/z for $[M+H]^+$ ion	Found m/z for $[M+H]^+$ ion (percentage relative intensity)	Mass measurement relative error (ppm)
**2**	318.1197	318.1237 (100%)	12.57

1. IUPAC, Compendium of Chemical Terminology Gold Book (Version 2.3.3), **2014**, p. 1296

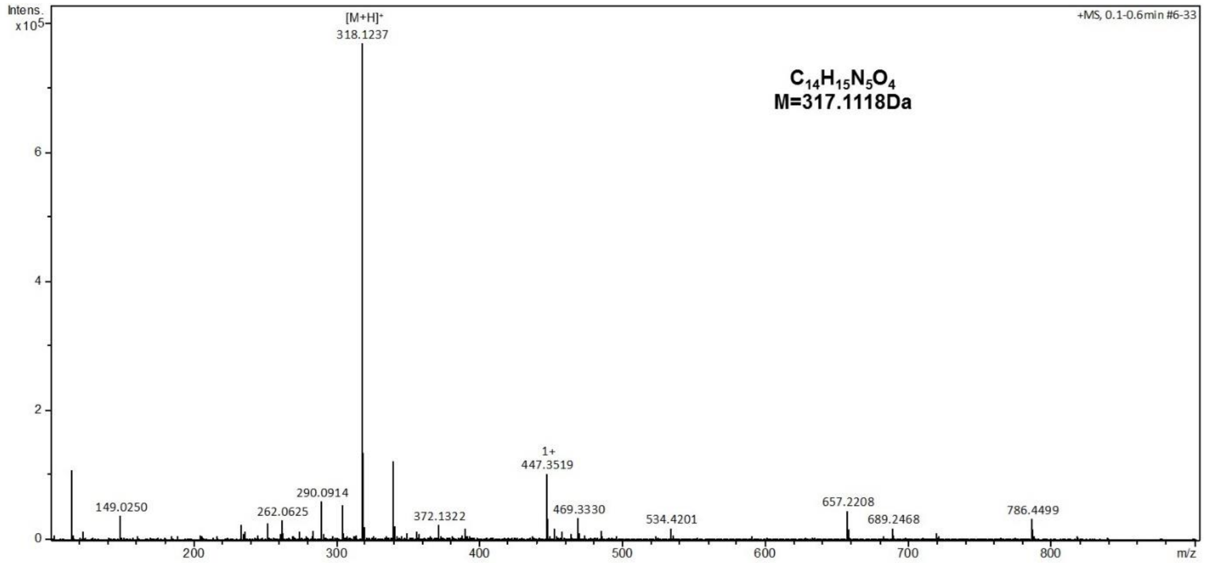

Figure S1. ESI-MS spectrum of the compound **2**.

Table S2. Analysis of other quasi-parent molecular ions of the compound **2**.

Other quasi-parent molecular ions of **2**	Calculated m/z	Found	
		m/z	Relative intensity (%)
$[M+H-C_2H_4]^+$	290.0884	290.0914	7.66
$[M+Na]^+$	340.1016	340.1056	15.86
$[2M+Na]^+$	657.2140	657.2208	5.78

Analysis and Visualizations of High Resolution Electrospray Ionization Mass Spectrometry (HR-ESI-MS) spectra of the compounds 3a-j

Table S3. Comparative set of masses of quasi-parent molecular $[M+H]^+$ ions of the compounds **3a-j**. Relative errors, resolution and the full width at half maximum (FWHM) associated with mass measurements of the compounds **3a-j**.

No.	Calculated m/z for $[M+H]^+$ ion	Found m/z for $[M+H]^+$ ion (percentage relative intensity)	Mass measurement relative error [ppm]	Resolution and FWHM (ΔM) [m/z]
**3a**	247.0938	247.0950 (100%)	4.86	20014<br>0.0123
**3b**	261.1096	261.1084 (100%)	4.60	20692<br>0.0126
**3c**	275.1251	275.1262 (100%)	4.00	21301<br>0.0129
**3d**	289.1408	289.1398 (100%)	3.17	19404<br>0.0149
**3e**	303.1564	303.1549 (100%)	4.95	22433<br>0.0135

**3f**	304.1516	304.1527 (100%)	3.62	20688<br>0.0147
**3g**	318.1673	318.1677 (100%)	1.26	22584<br>0.0141
**3h**	277.1044	277.1053 (100%)	3.25	21570<br>0.0128
**3i**	291.1200	291.1214 (100%)	4.81	20639<br>0.0141
**3j**	346.1622	346.1639 (100%)	4.91	21990<br>0.0157

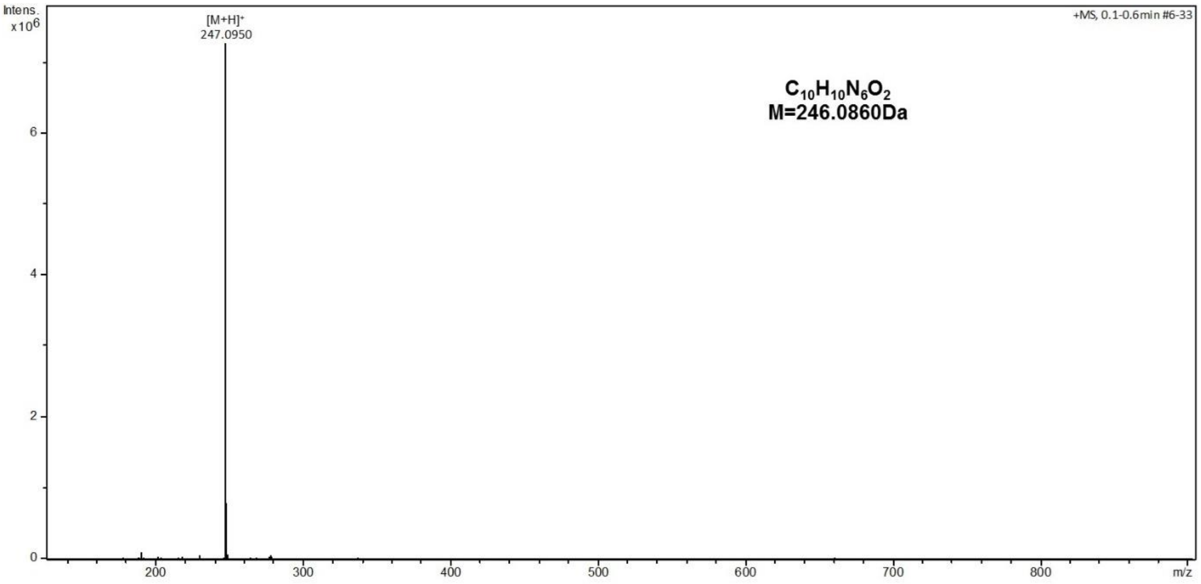

Figure S2. HR-ESI-MS spectrum of the compound **3a**.

Table S4. Analysis of other quasi-parent molecular ions of the compound **3a**.

Other quasi-parent molecular ions of **3a**	Calculated m/z	Found	
		m/z	Relative intensity (%)
$[M+H-NH_3]^+$	230.0673	230.0679	0.62
$[M+Na]^+$	269.0757	269.0763	0.24
$[M+CH_3OH+H]^+$	279.1200	279.1225	0.49
$[2M+Na]^+$	515.1623	515.1630	0.08

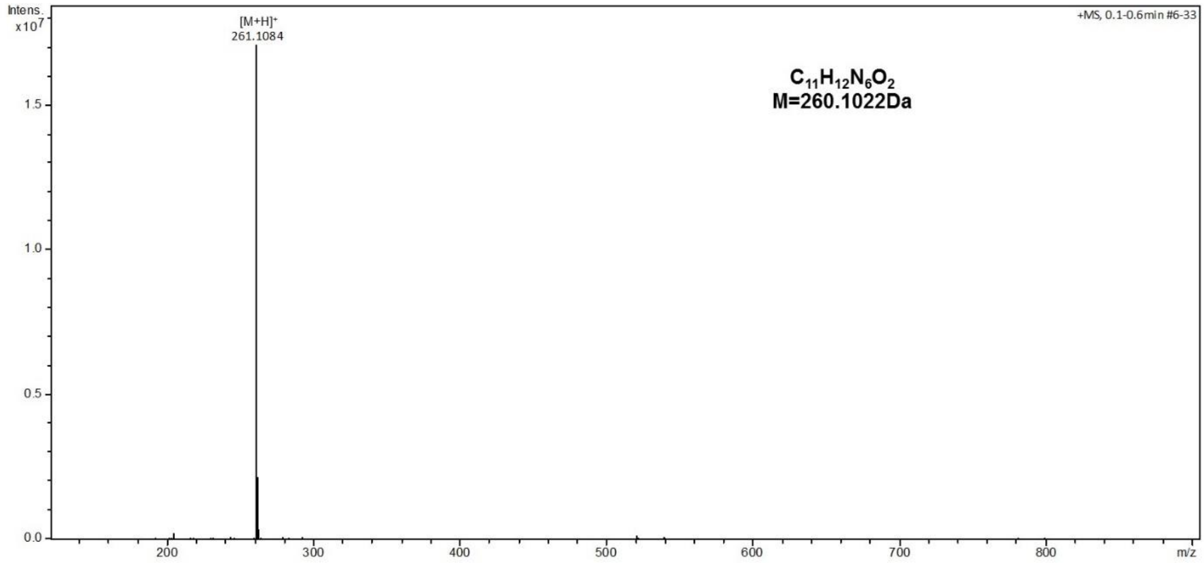

Figure S3. HR-ESI-MS spectrum of the compound **3b**.

Table S5. Analysis of other quasi-parent molecular ions of the compound **3b**.

Other quasi-parent molecular ions of **3b**	Calculated m/z	Found	
		m/z	Relative intensity (%)
$[M+H-NH3]^+$	244.0829	244.0826	0.56
$[M+Na]^+$	283.0914	283.0905	0.15
$[M+CH_3OH+H]^+$	293.1357	293.1349	0.57
$[2M+H]^+$	521.2116	521.2096	0.60
$[2M+Na]^+$	543.1936	543.1916	0.05

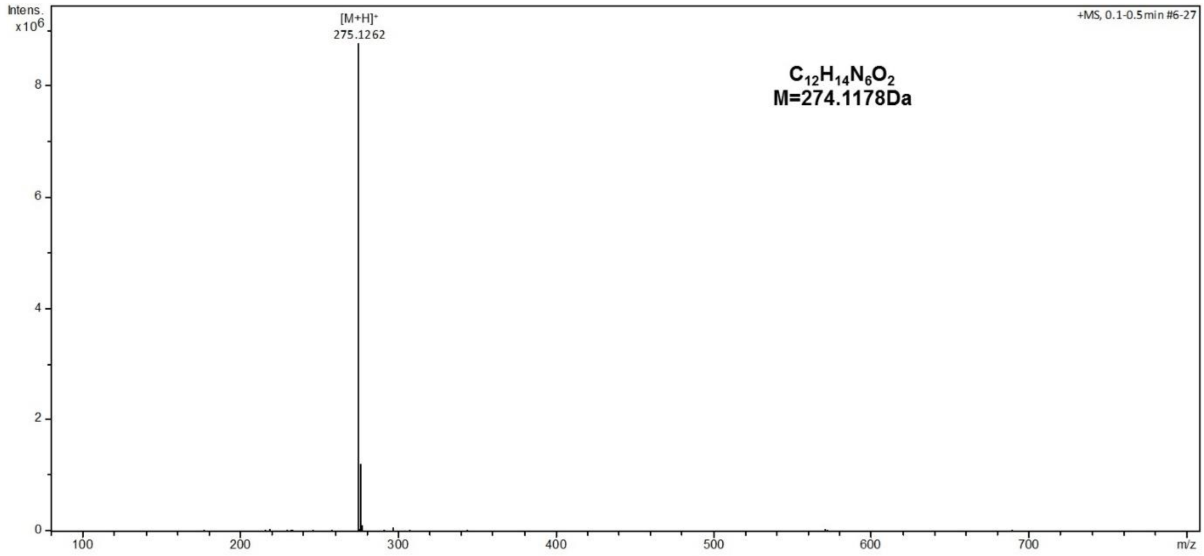

Figure S4. HR-ESI-MS spectrum of the compound **3c**.

Table S6. Analysis of other quasi-parent molecular ions of the compound **3c**.

Other quasi-parent molecular ions of **3c**	Calculated m/z	Found	
		m/z	Relative intensity (%)
$[M+H-NH_3]^+$	258.0986	258.0995	0.22
$[M+Na]^+$	297.1070	297.1084	0.48
$[M+CH_3OH+H]^+$	307.1513	307.1529	0.22
$[2M+Na]^+$	571.2249	571.2280	0.26

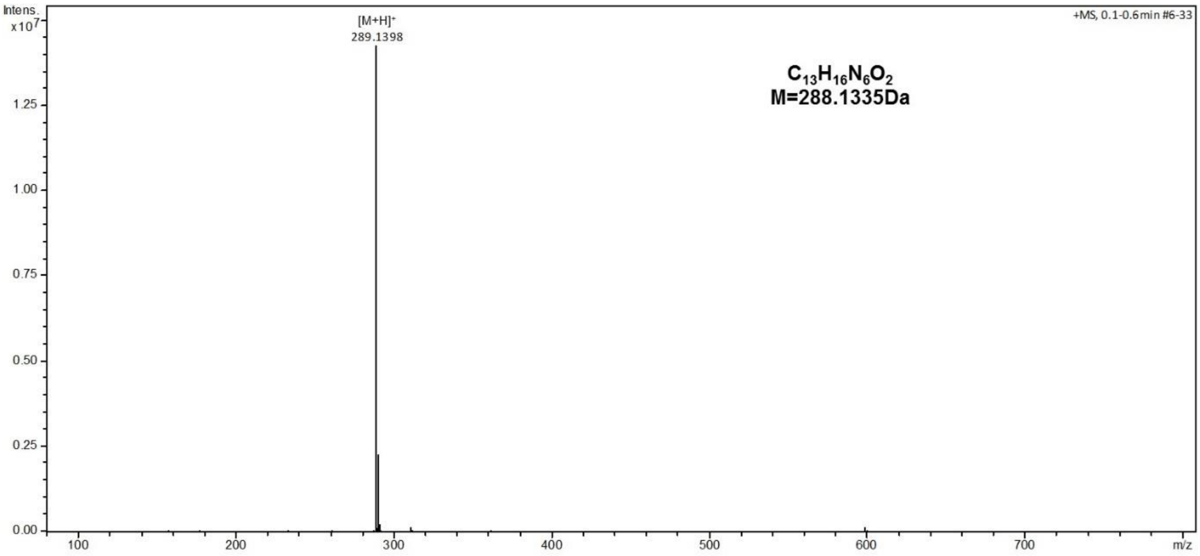

Figure S5. HR-ESI-MS spectrum of the compound **3d**.

Table S7. Analysis of other quasi-parent molecular ions of the compound **3d**.

Other quasi-parent molecular ions of **3d**	Calculated m/z	Found	
		m/z	Relative intensity (%)
$[M+Na]^+$	311.1227	311.1215	0.84
$[2M+Na]^+$	599.2562	599.2541	0.84

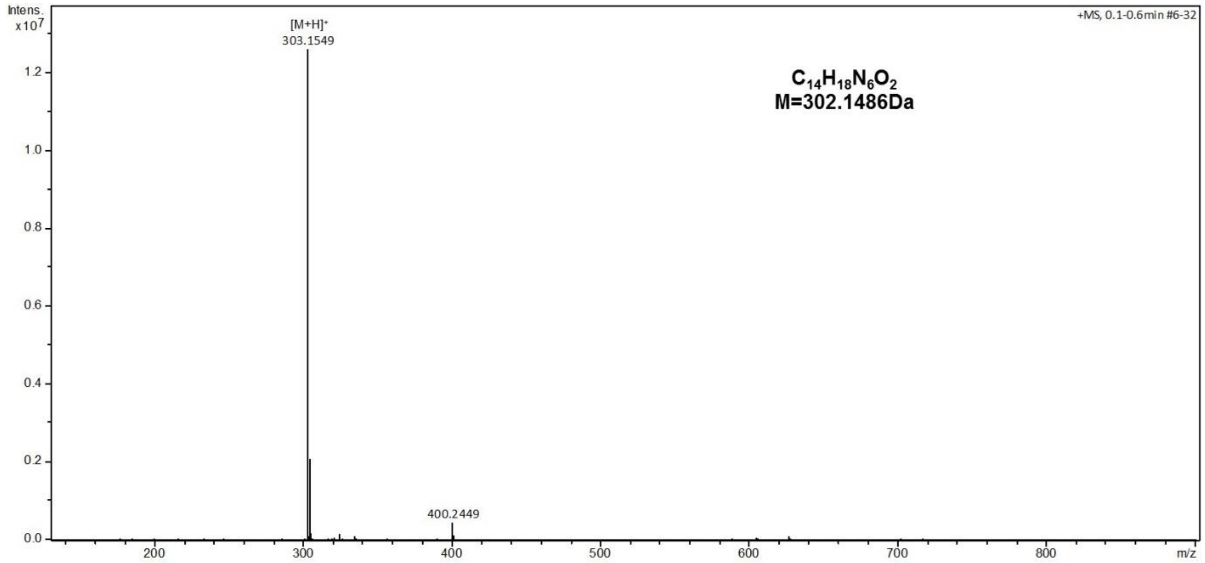

Figure S6. HR-ESI-MS spectrum of the compound **3e**.

Table S8. Analysis of other quasi-parent molecular ions of the compound **3e**.

Other quasi-parent molecular ions of **3e**	Calculated m/z	Found	
		m/z	Relative intensity (%)
$[M+Na]^+$	325.1383	325.1371	1.22
$[M+CH_3OH+H]^+$	335.1826	335.1809	0.67
$[M+Na+CH_3CN+2NH_4-2H]^+$	400.2180	400.2449	3.61
$[2M+H]^+$	605.3055	605.3041	0.43
$[2M+Na]^+$	627.2875	627.2863	0.69

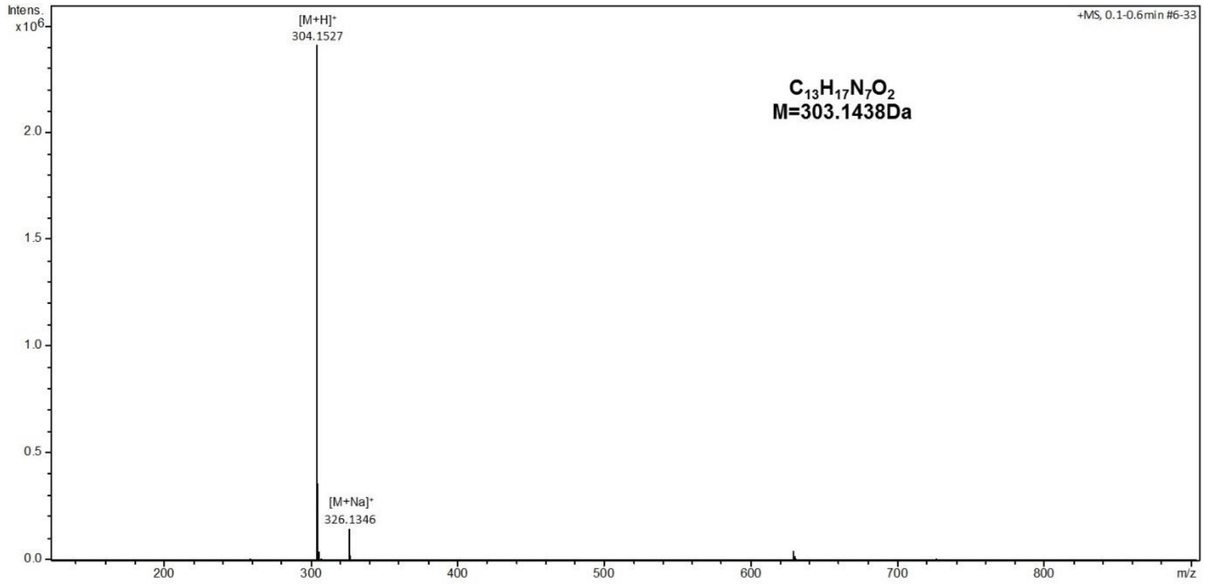

Figure S7. HR-ESI-MS spectrum of the compound **3f**.

Table S9. Analysis of other quasi-parent molecular ions of the compound **3f**.

Other quasi-parent molecular ions of **3f**	Calculated m/z	Found	
		m/z	Relative intensity (%)
$[M+Na]^+$	326.1336	326.1346	6.10
$[M+CH_3OH+H]^+$	336.1779	336.1788	0.07
$[2M+Na]^+$	629.2780	629.2805	1.84

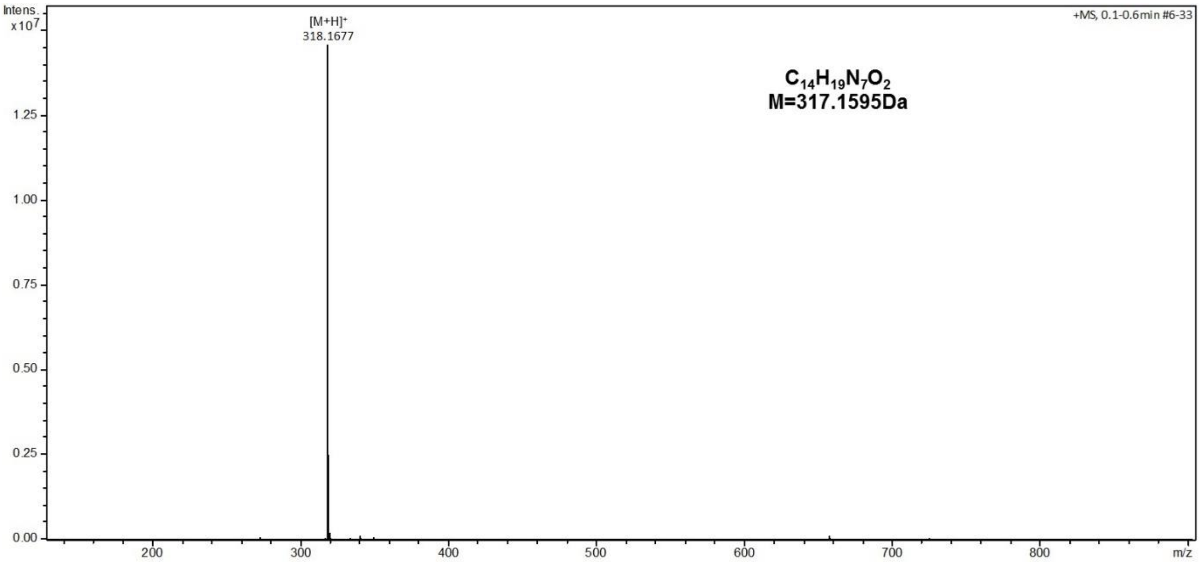

Figure S8. HR-ESI-MS spectrum of the compound **3g**.

Table S10. Analysis of other quasi-parent molecular ions of the compound **3g**.

Other quasi-parent molecular ions of **3g**	Calculated m/z	Found	
		m/z	Relative intensity (%)
$[M+Na]^+$	340.1492	340.1497	0.76
$[M+CH_3OH+H]^+$	350.1935	350.1938	0.56
$[2M+Na]^+$	657.3093	657.3113	0.65

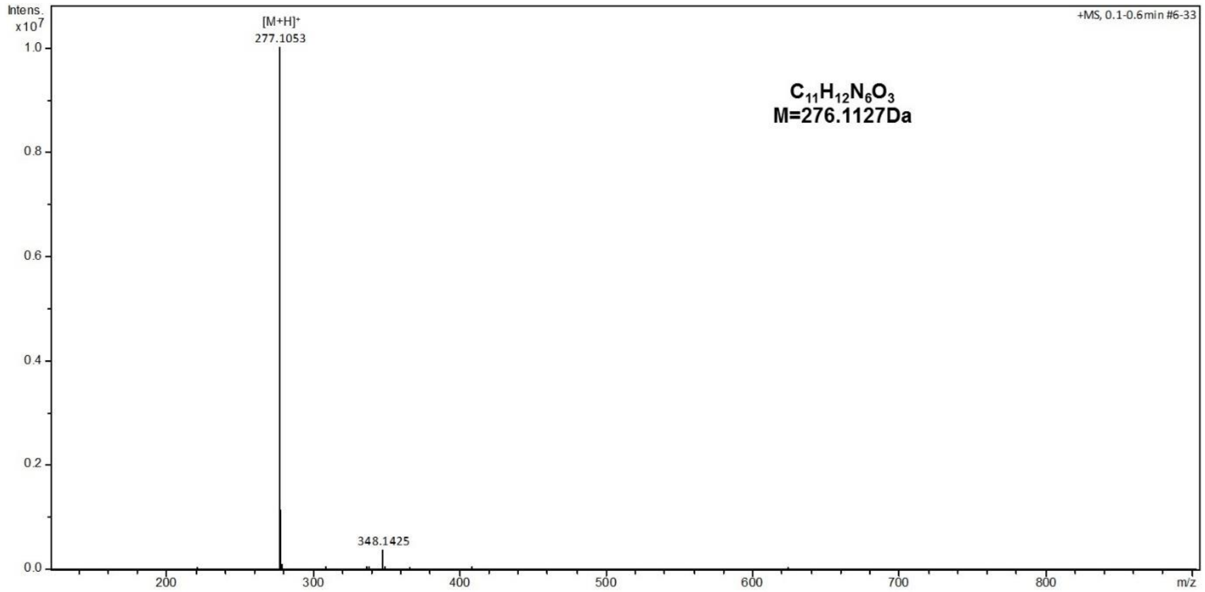

Figure S9. HR-ESI-MS spectrum of the compound **3h**.

Table S11. Analysis of other quasi-parent molecular ions of the compound **3h**.

Other quasi-parent molecular ions of **3h**	Calculated m/z	Found	
		m/z	Relative intensity (%)
$[M+Na]^+$	299.0863	299.0865	0.14
$[M+CH_3OH+H]^+$	309.1306	309.1309	0.68
$[M+Na+K-H]^+$	337.0422	337.1413	0.65
$[M+CH_3OH+Na+NH_4-H]^+$	348.1391	348.1425	3.78
$[2M+H]^+$	553.2015	553.2023	0.11
$[2M+CH_3OH+Na+NH_4-H]^+$	624.2362	624.2396	0.36

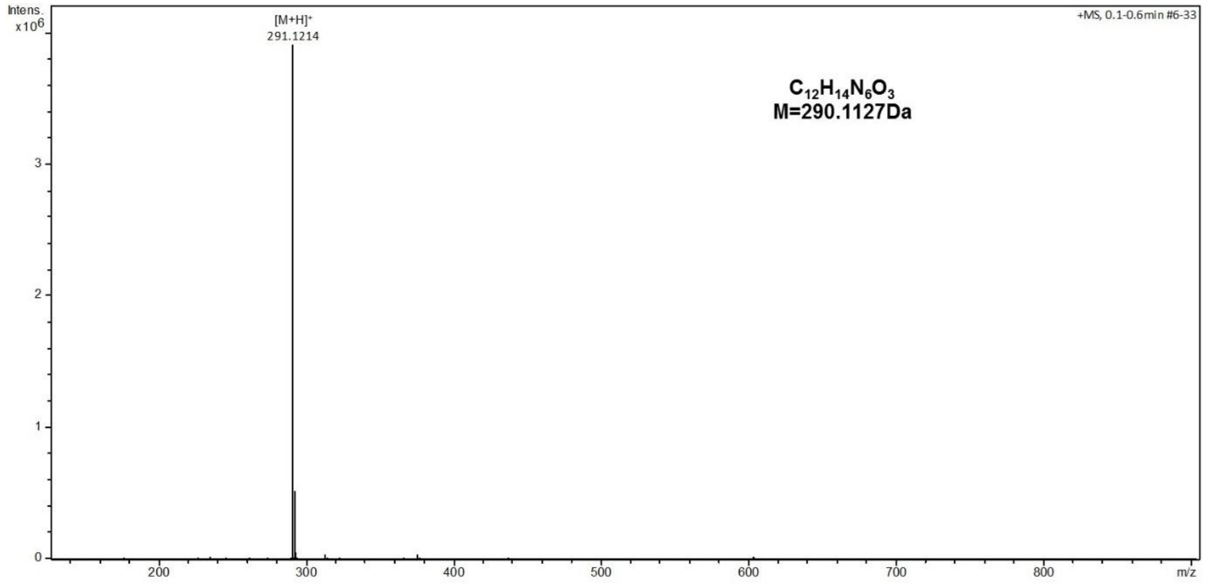

Figure S10. HR-ESI-MS spectrum of the compound **3i**.

Table S12. Analysis of other quasi-parent molecular ions of the compound **3i**.

Other quasi-parent molecular ions of **3i**	Calculated m/z	Found	
		m/z	Relative intensity (%) to base peak
$[M+Na]^+$	313.1020	313.1023	0.94
$[M+CH_3OH+H]^+$	323.1462	323.1467	0.21
$[2M+Na]^+$	603.2147	603.2153	0.36

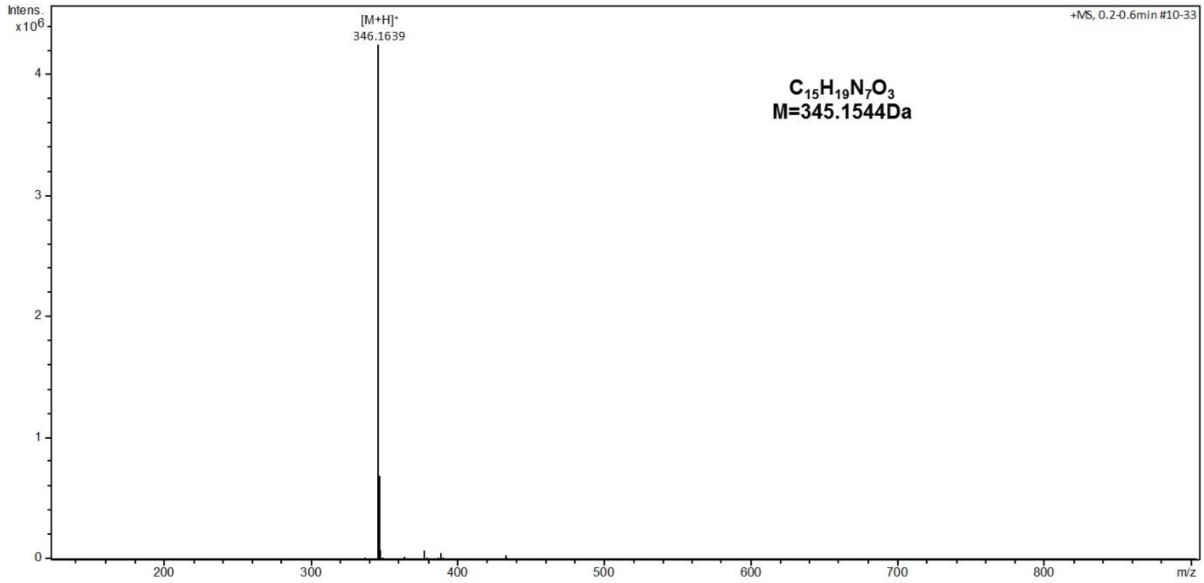

Figure S11. HR-ESI-MS spectrum of the compound **3j**.

Table S13. Analysis of other quasi-parent molecular ions of the compound **3j**.

Other quasi-parent molecular ions of **3j**	Calculated m/z	Found	
		m/z	Relative intensity (%) to base peak
$[M+Na]^+$	368.1442	368.1451	0.09
$[M+CH_3OH+H]^+$	378.1884	368.1891	1.48
$[2M+H]^+$	691.3171	691.3189	0.08

Visualizations of Nuclear magnetic resonance (NMR) and Infrared (IR) spectra of intermediate 2

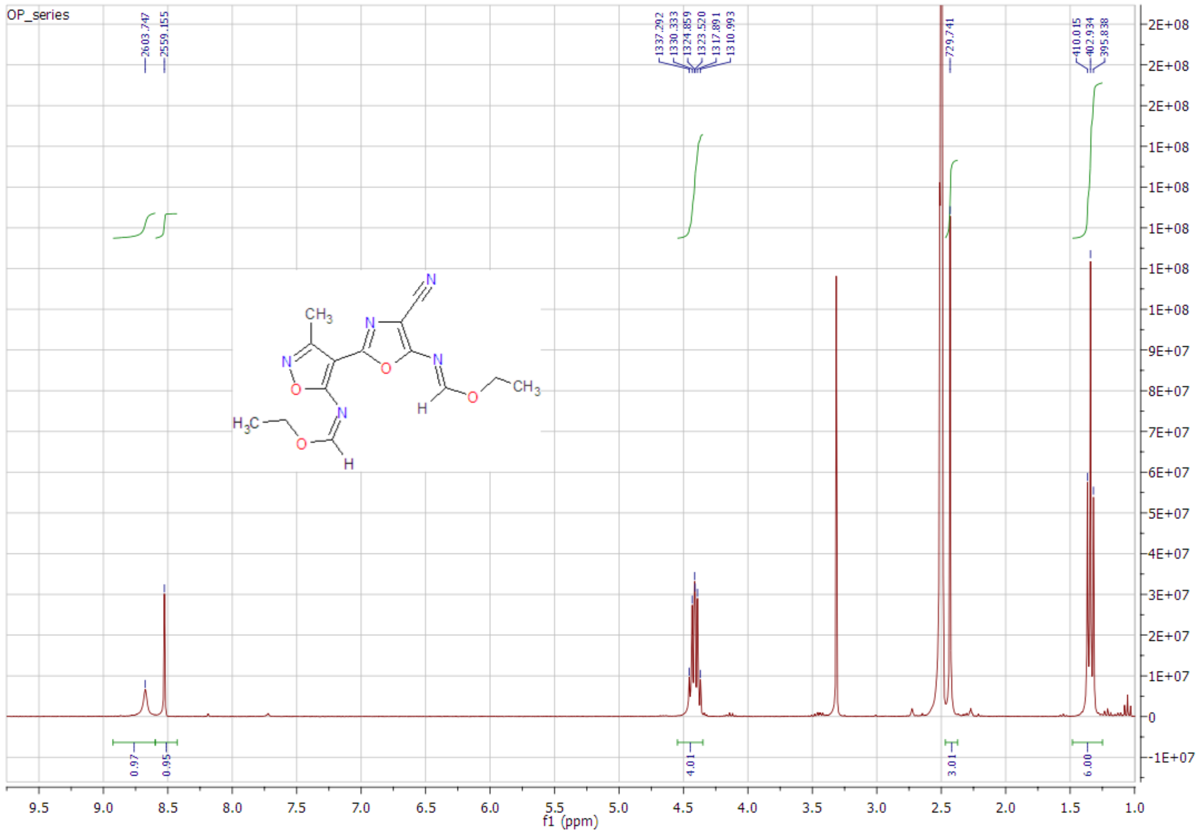

Figure S12. ^{1}H-NMR spectrum of intermediate **2** in DMSO-d_6.

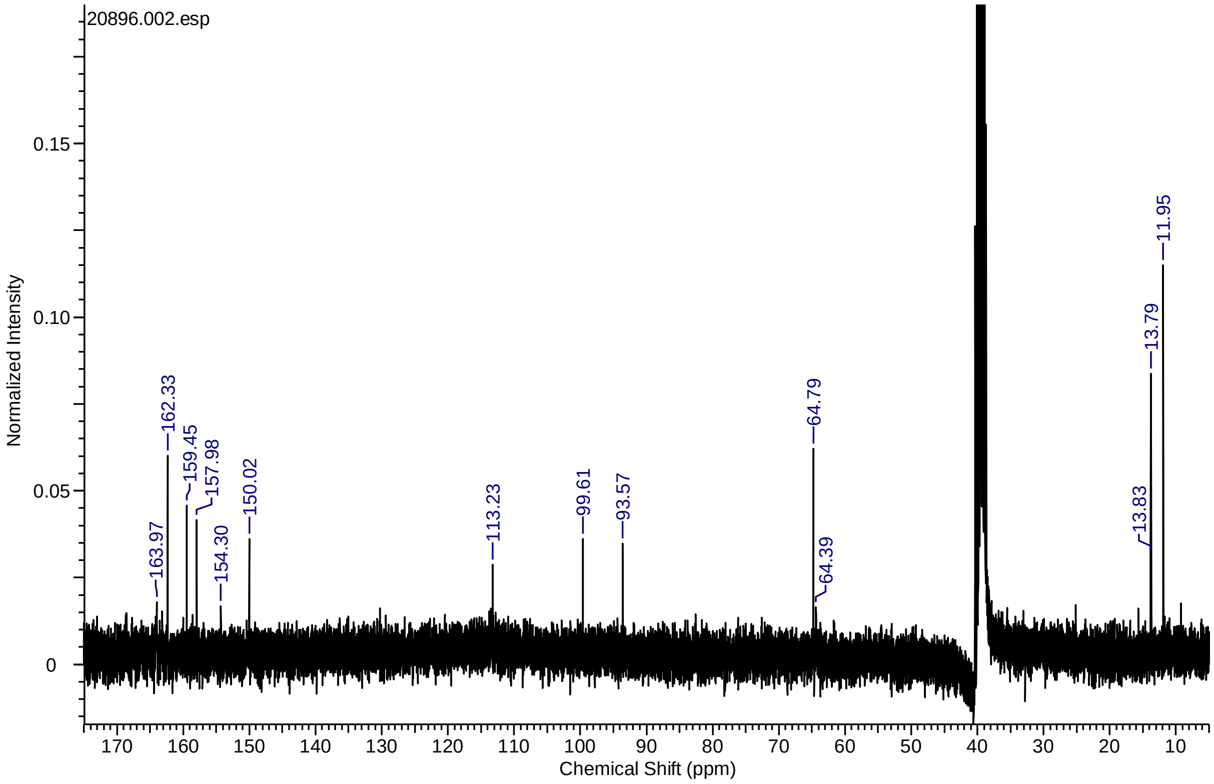

Figure S13. ^{13}C-NMR spectrum of intermediate **2** in DMSO-d_6.

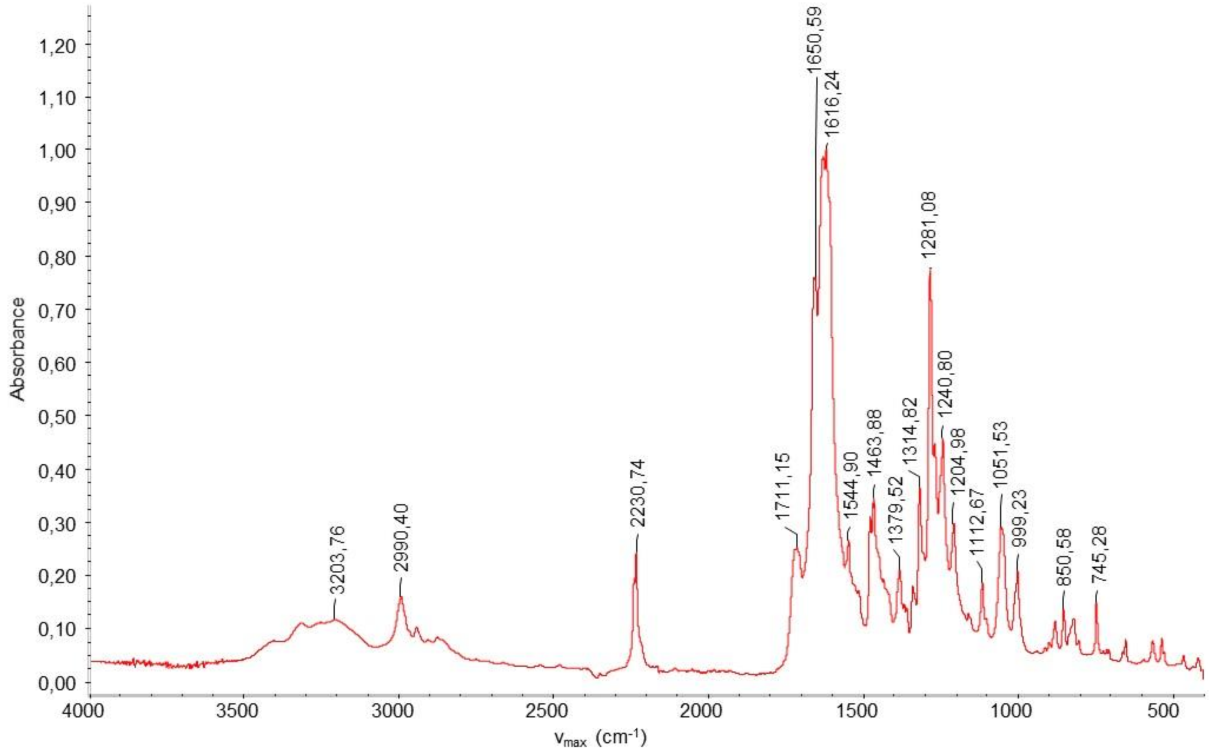

Figure S14. ATR-FTIR spectrum of intermediate **2**.

Visualizations of Nuclear magnetic resonance (NMR) and Infrared (IR) spectra of the compound 3a

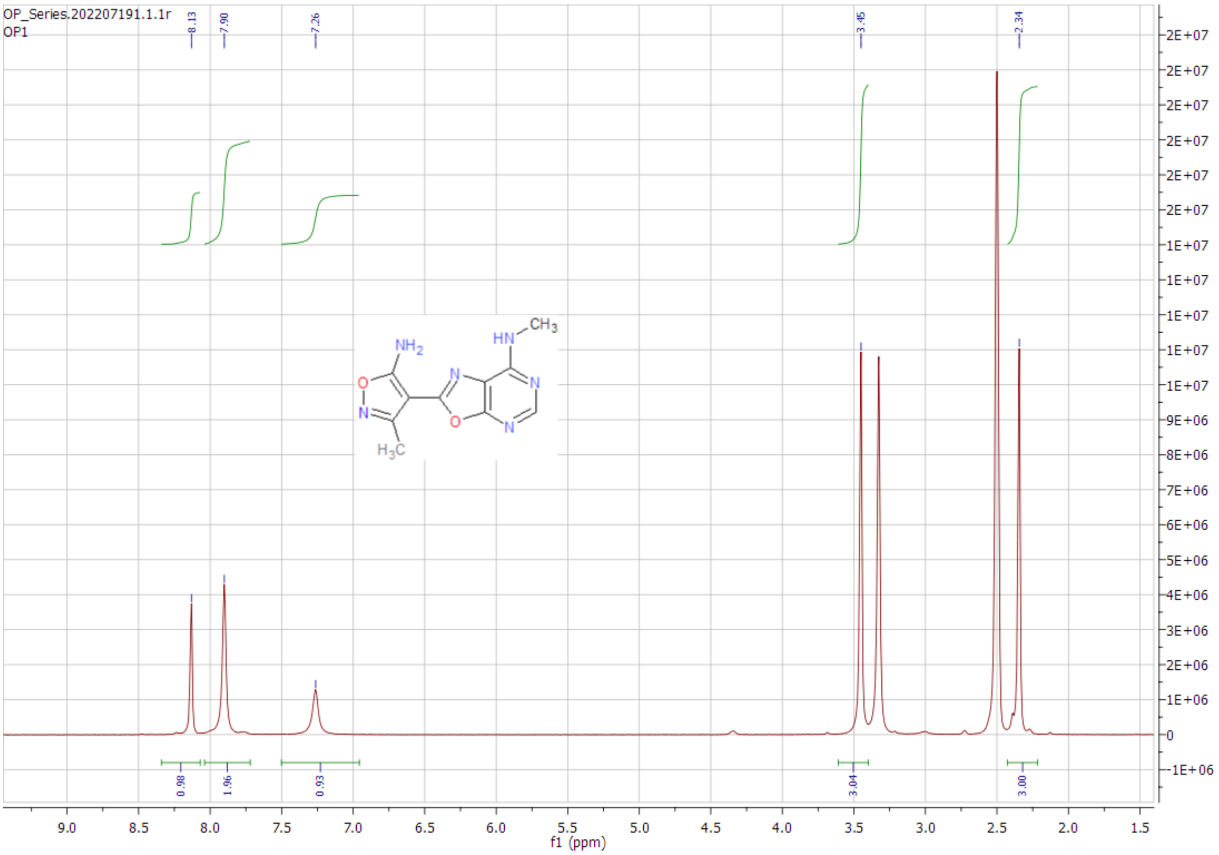

Figure S15. ^{1}H-NMR spectrum of the compound **3a** in DMSO-d_6.

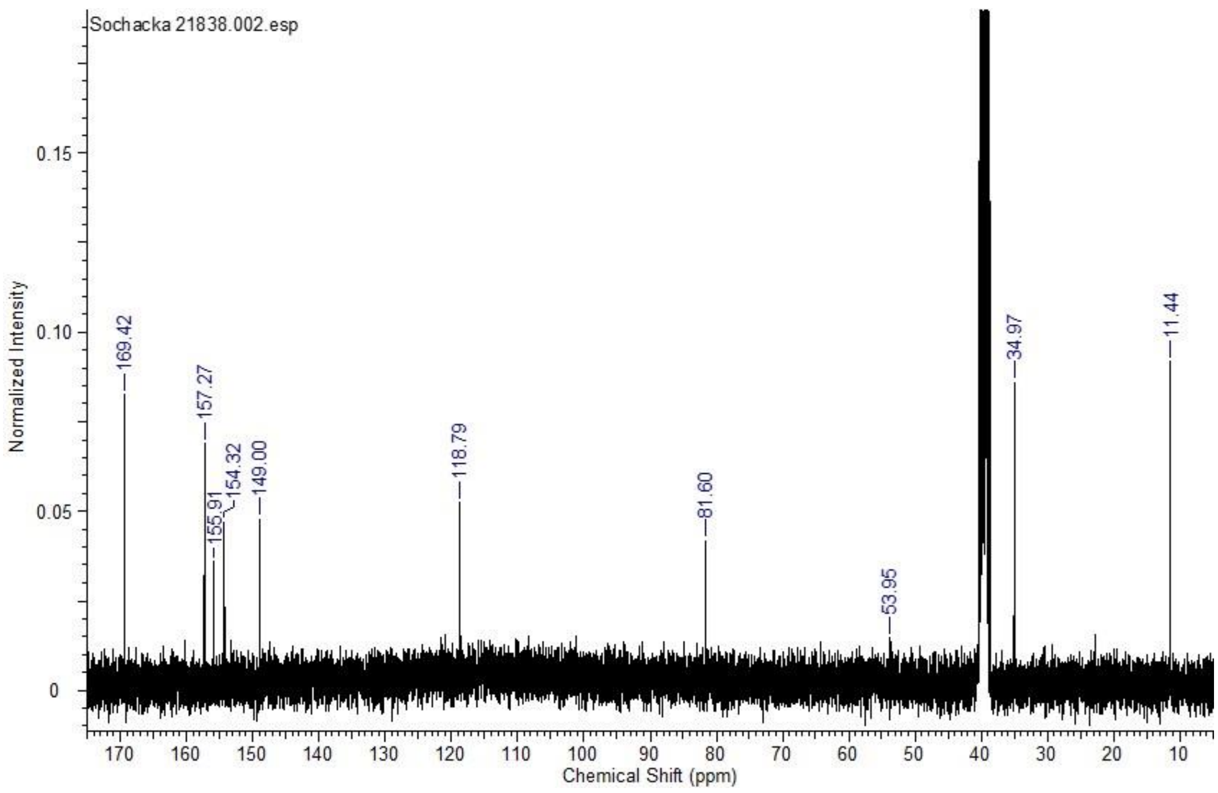

Figure S16. ^{13}C-NMR spectrum of the compound **3a** in DMSO-d_6.

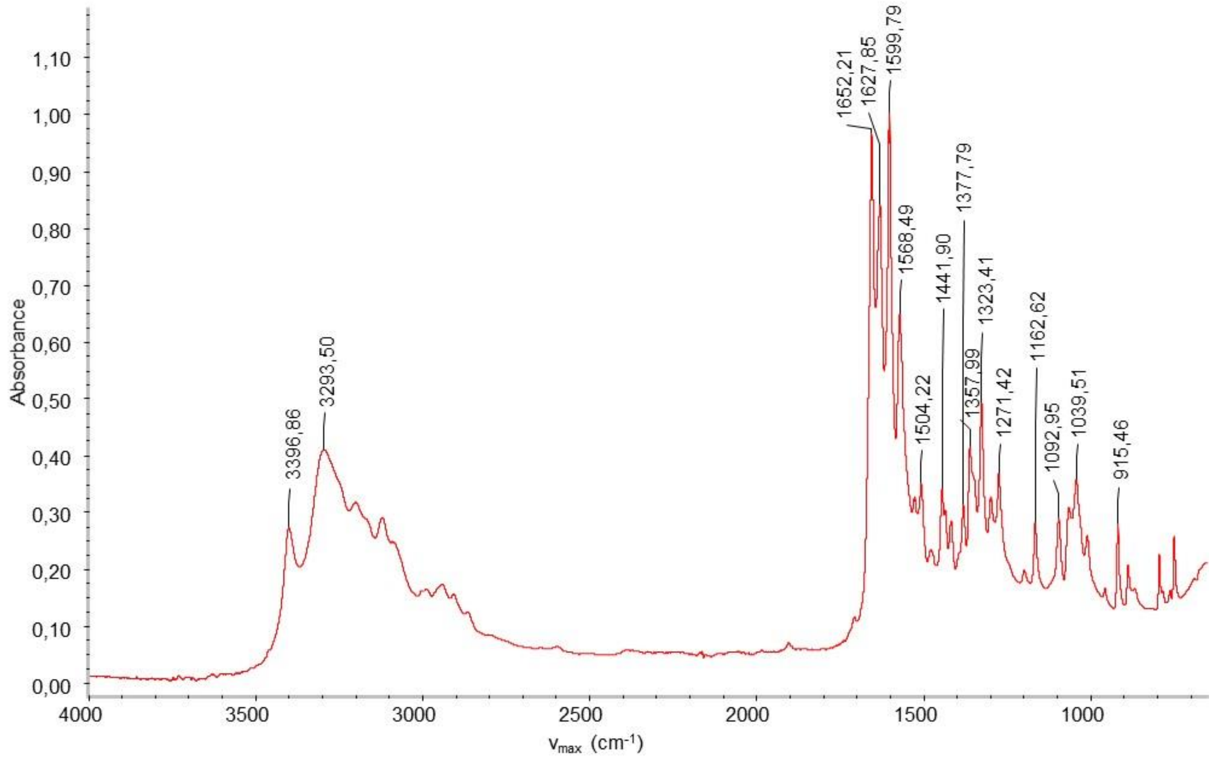

Figure S17. ATR-FTIR spectrum of the compound **3a**.

Visualizations of Nuclear magnetic resonance (NMR) and Infrared (IR) spectra of the compound 3b

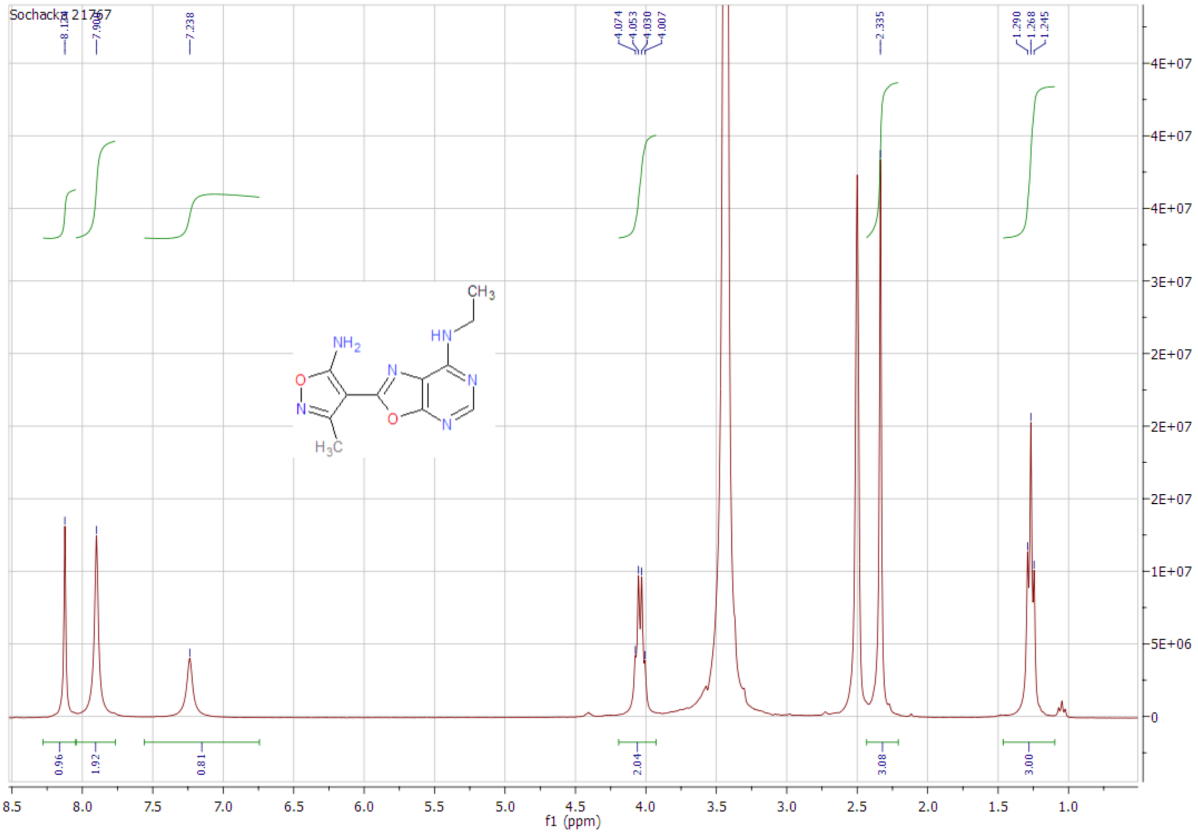

Figure S18. ^{1}H-NMR spectrum of the compound **3b** in DMSO-d_6.

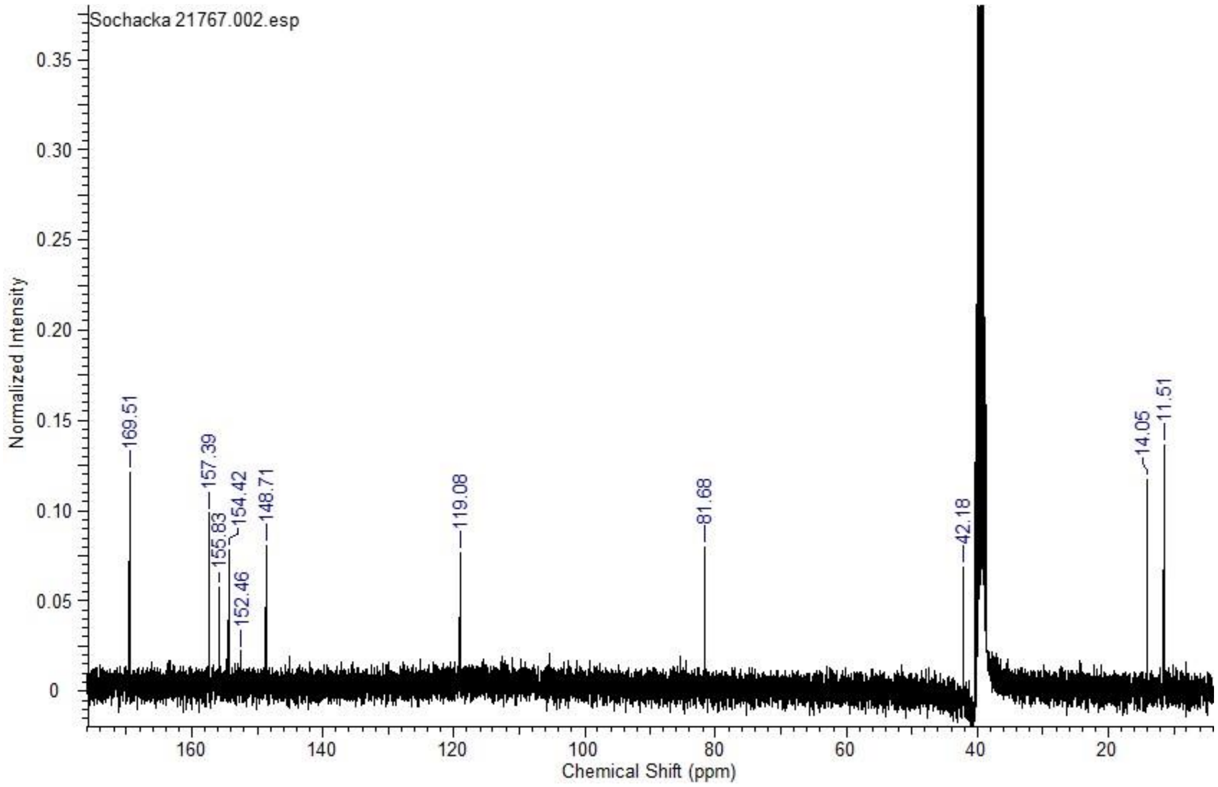

Figure S19. ^{13}C-NMR spectrum of the compound **3b** in DMSO-d_6.

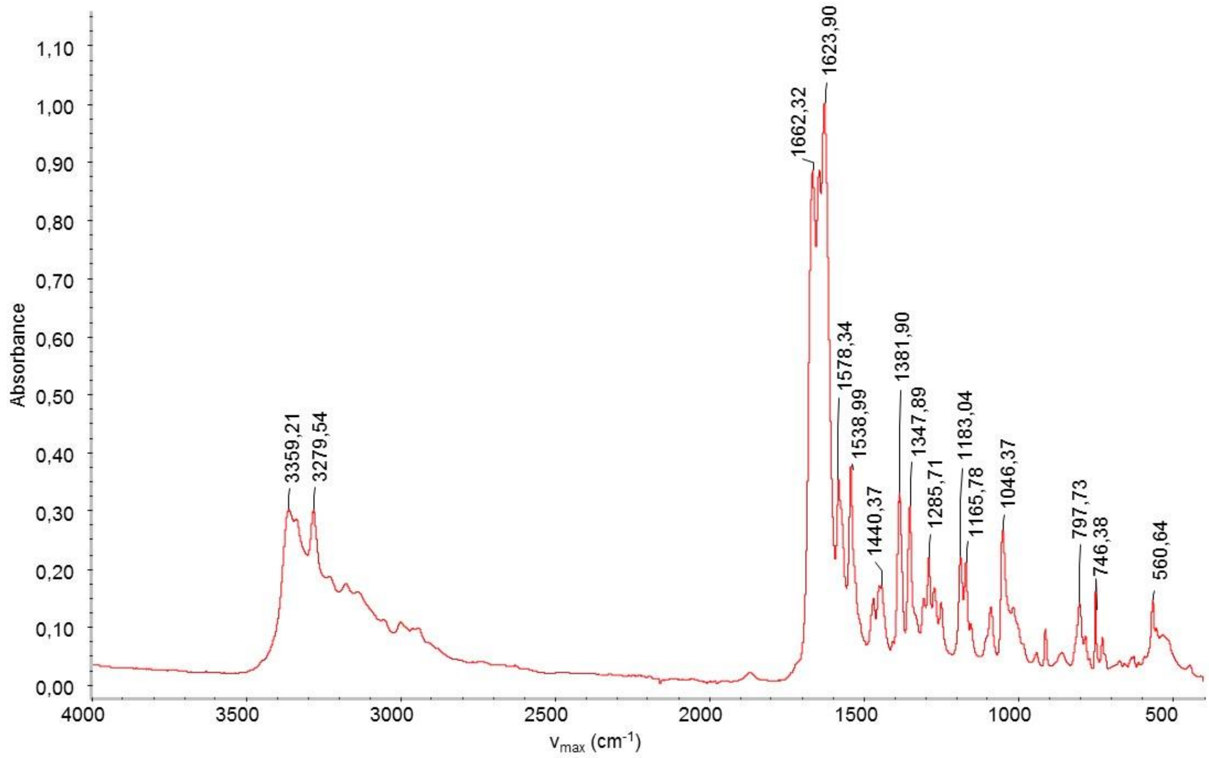

Figure S20. ATR-FTIR spectrum of the compound **3b**.

Visualizations of Nuclear magnetic resonance (NMR) and Infrared (IR) spectra of the compound 3c

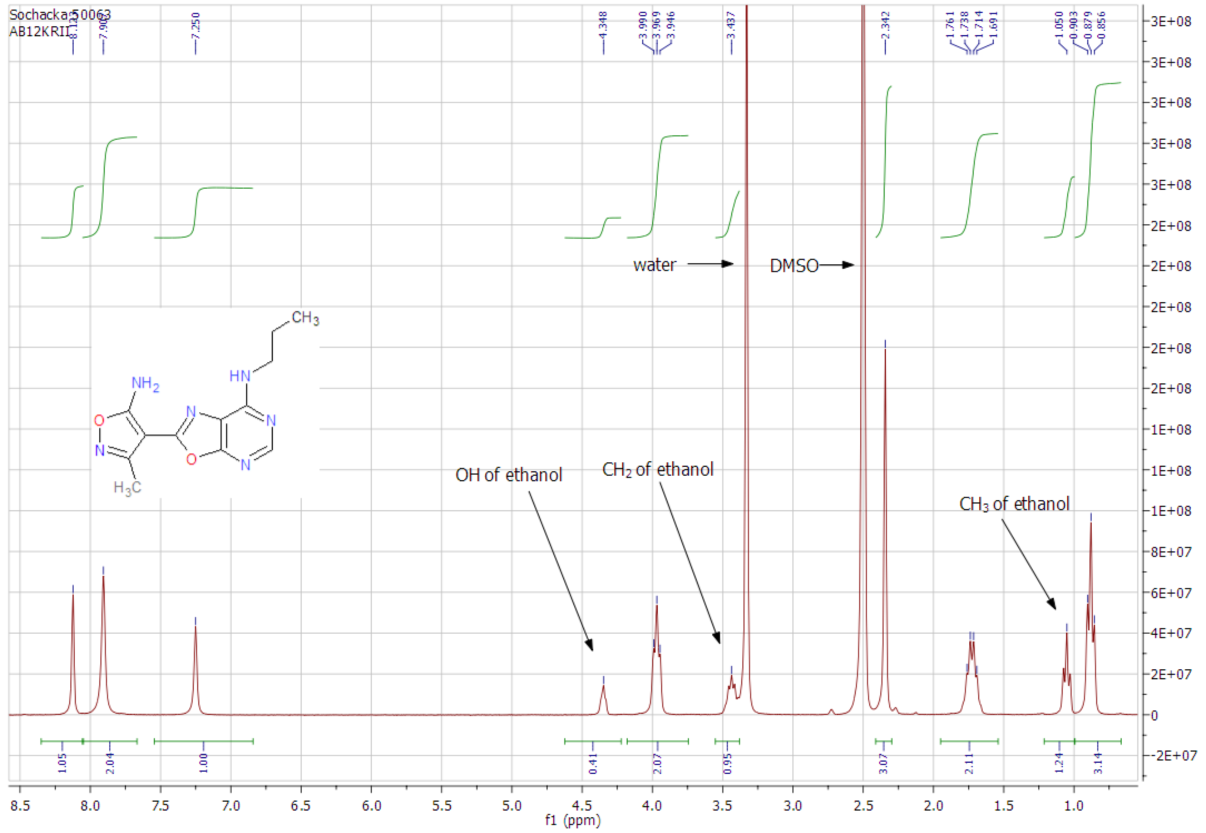

Figure S21. ^{1}H-NMR spectrum of the compound **3c** in DMSO-d_6.

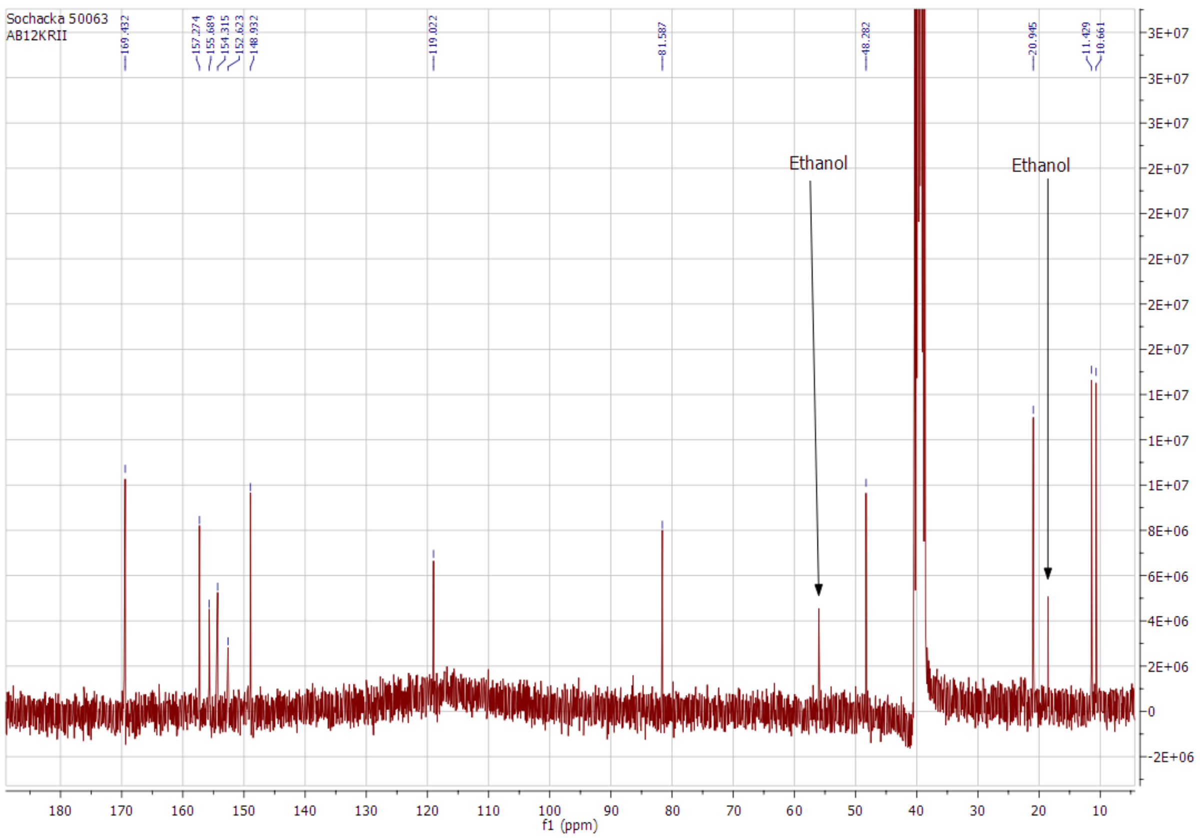

Figure S22. ^{13}C-NMR spectrum of the compound **3c** in DMSO-d_6.

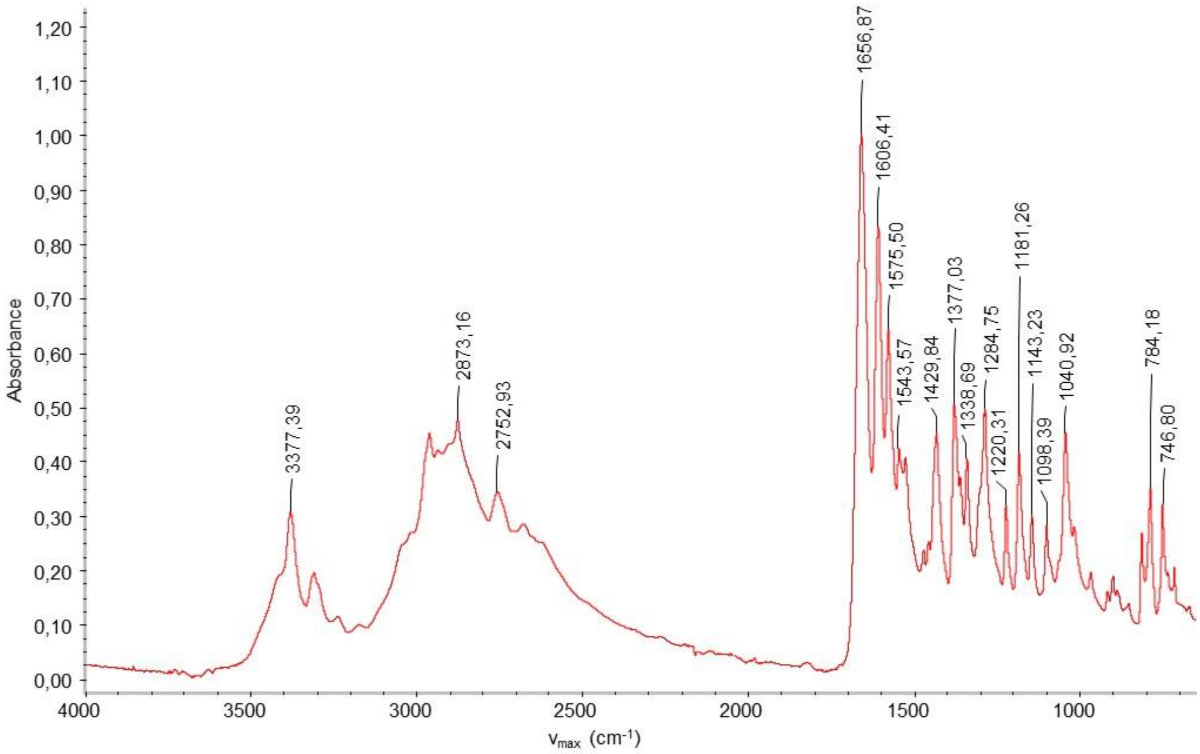

Figure S23. ATR-FTIR spectrum of the compound **3c**.

Visualizations of Nuclear magnetic resonance (NMR) and Infrared (IR) spectra of the compound 3d

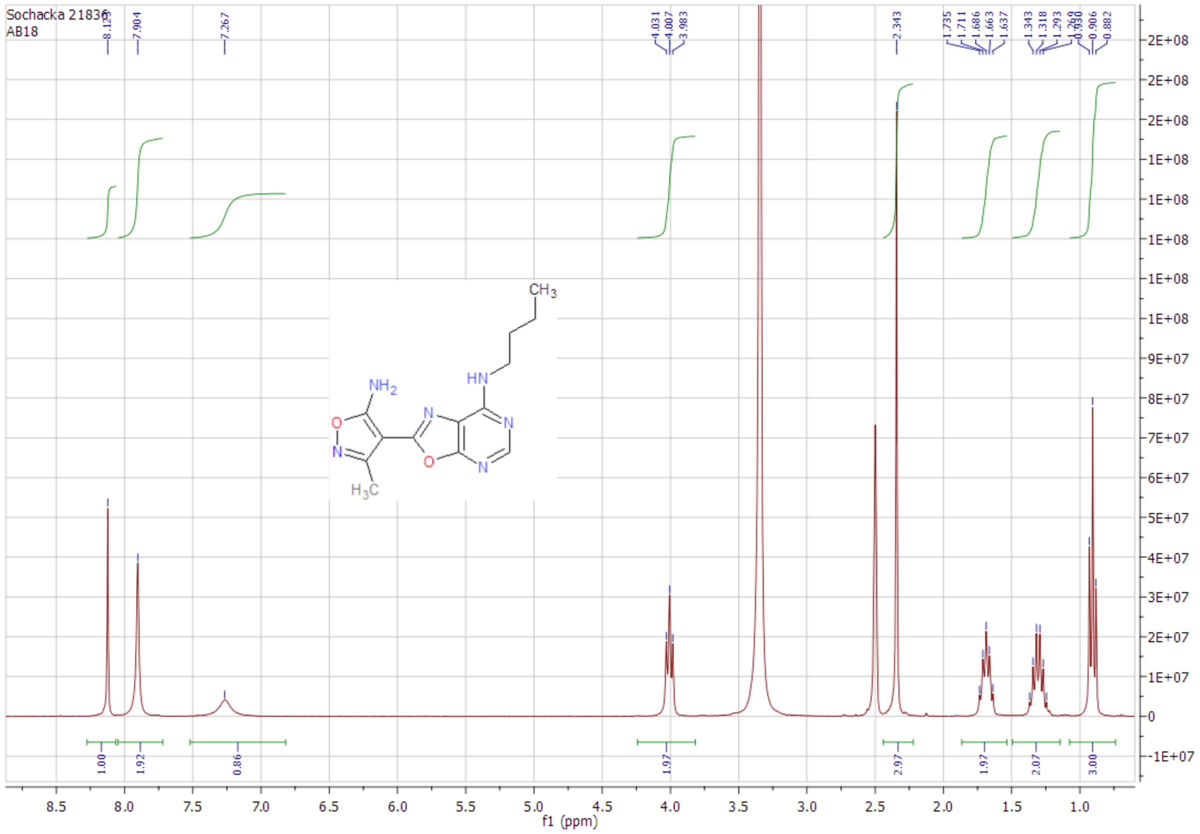

Figure S24. ^{1}H-NMR spectrum of the compound **3d** in DMSO-d_6.

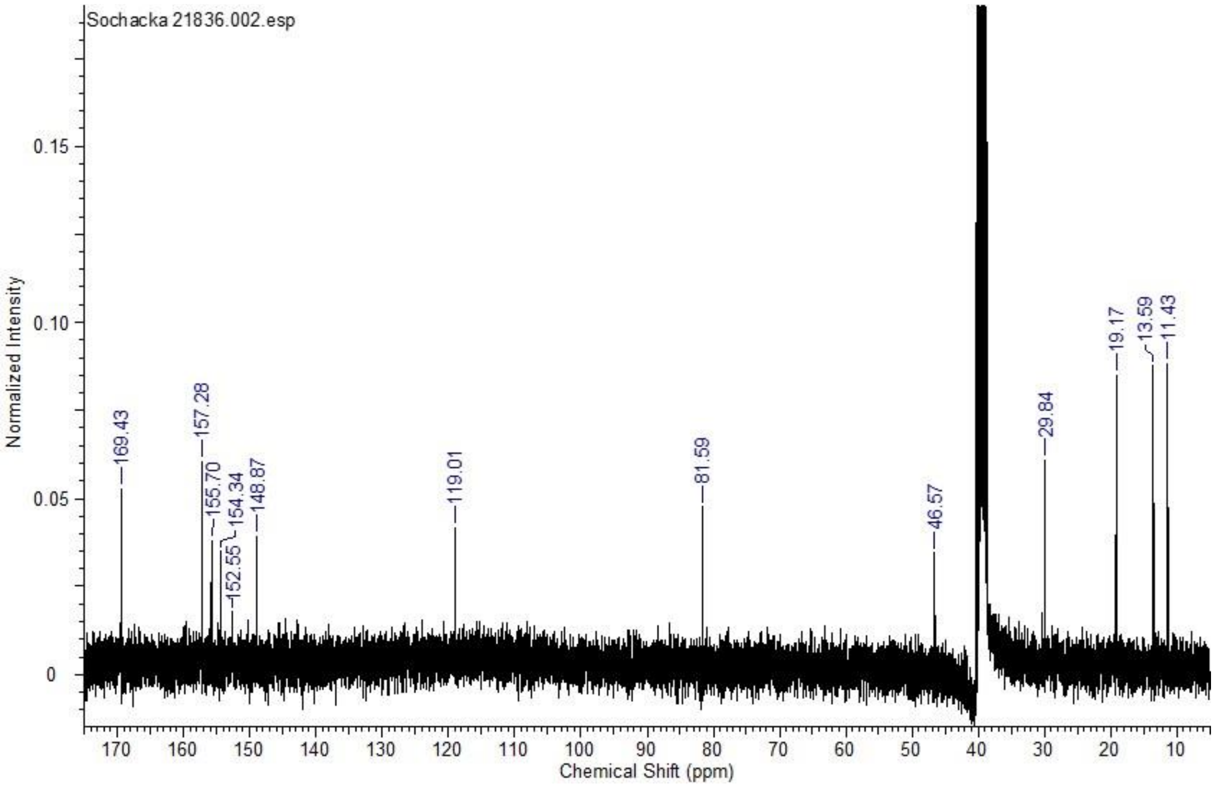

Figure S25. ^{13}C-NMR spectrum of the compound **3d** in DMSO-d_6.

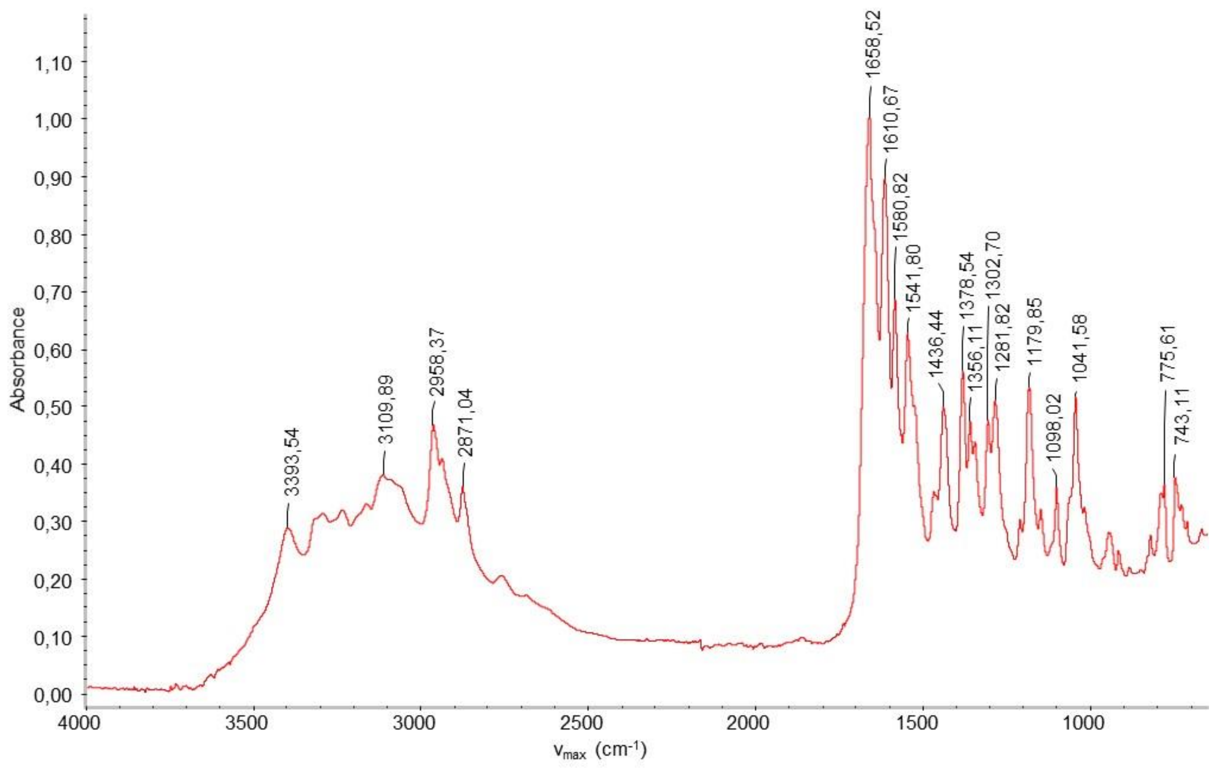

Figure S26. ATR-FTIR spectrum of the compound **3d**.

Visualizations of Nuclear magnetic resonance (NMR) and Infrared (IR) spectra of the compound 3e

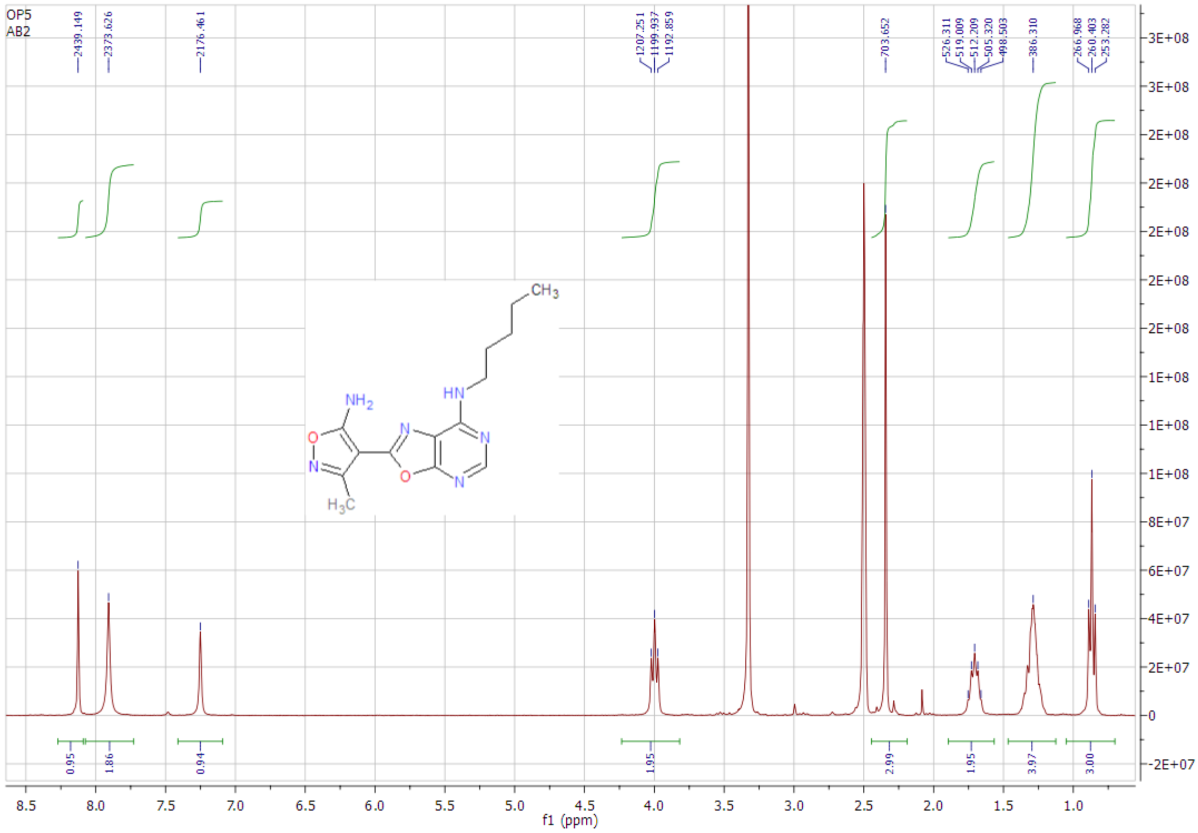

Figure S27. ^{1}H-NMR spectrum of the compound **3e** in DMSO-d_6.

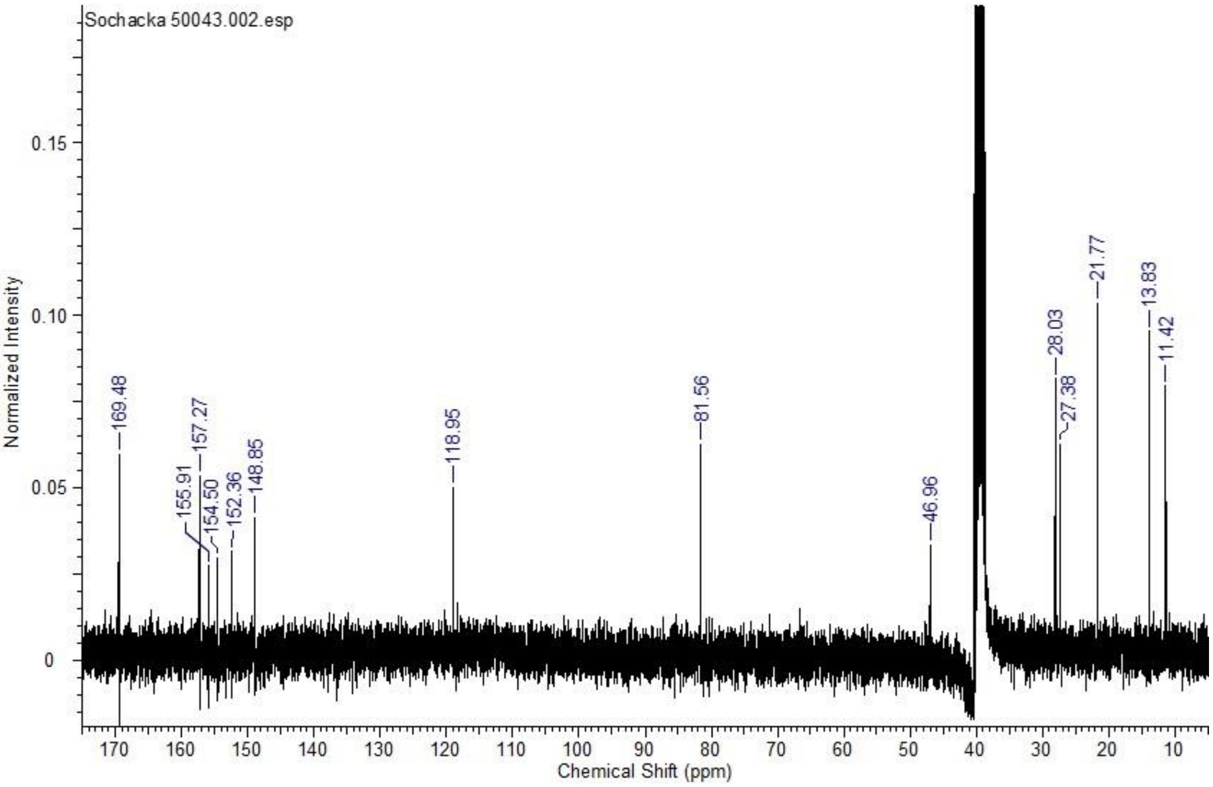

Figure S28. ^{13}C-NMR spectrum of the compound **3e** in DMSO-d_6.

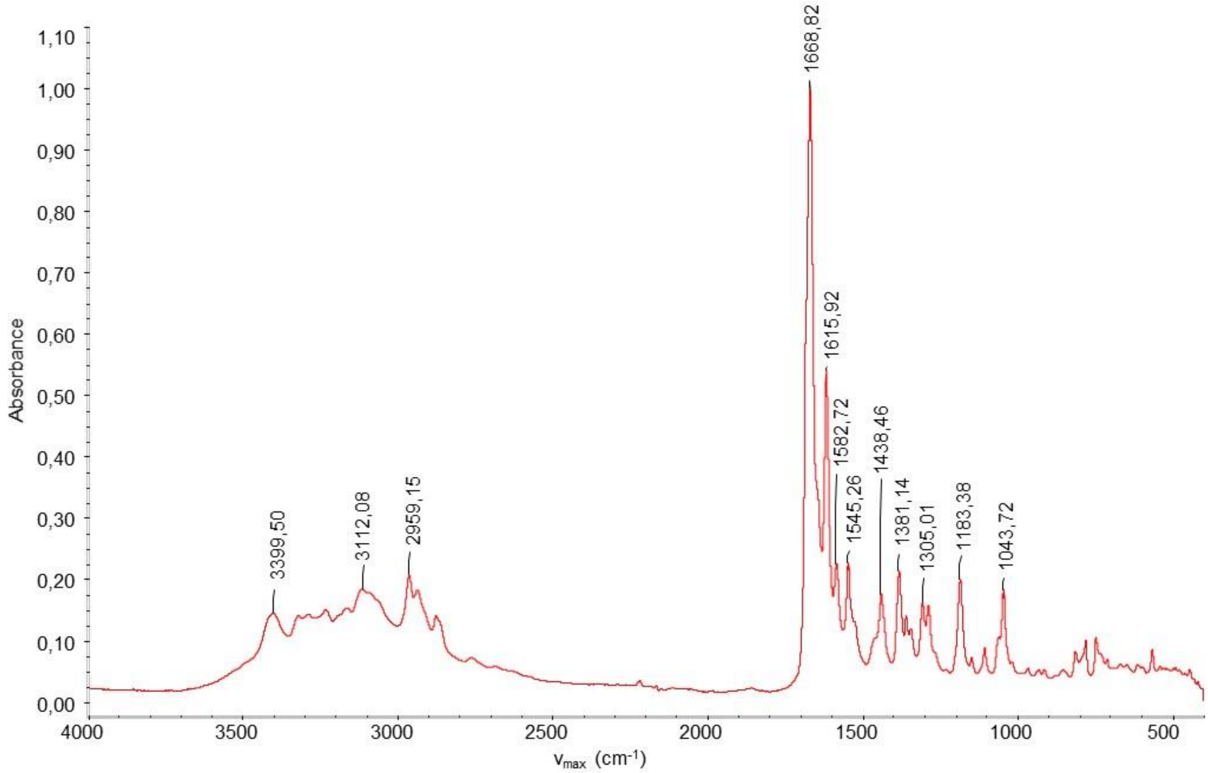

Figure S29. ATR-FTIR spectrum of the compound **3e**.

Visualizations of Nuclear magnetic resonance (NMR) and Infrared (IR) spectra of the compound 3f

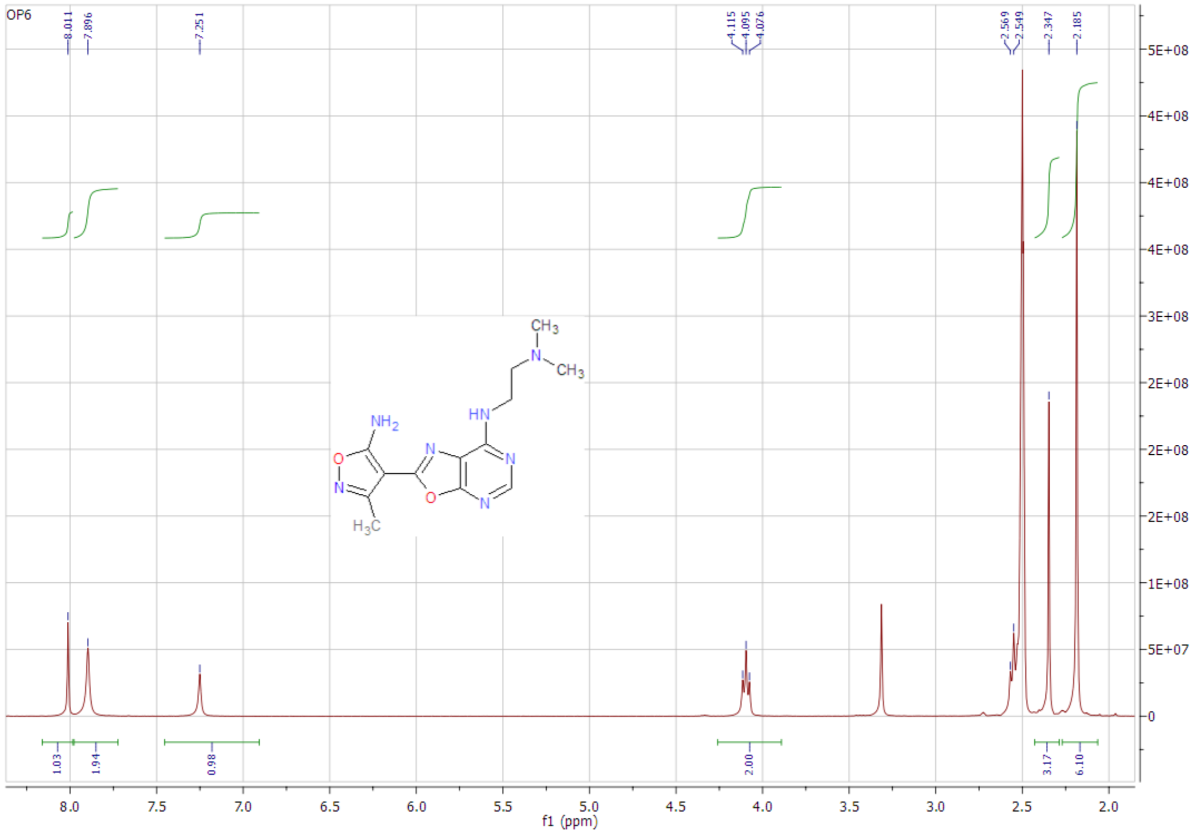

Figure S30. ^{1}H-NMR spectrum of the compound **3f** in DMSO-d_6.

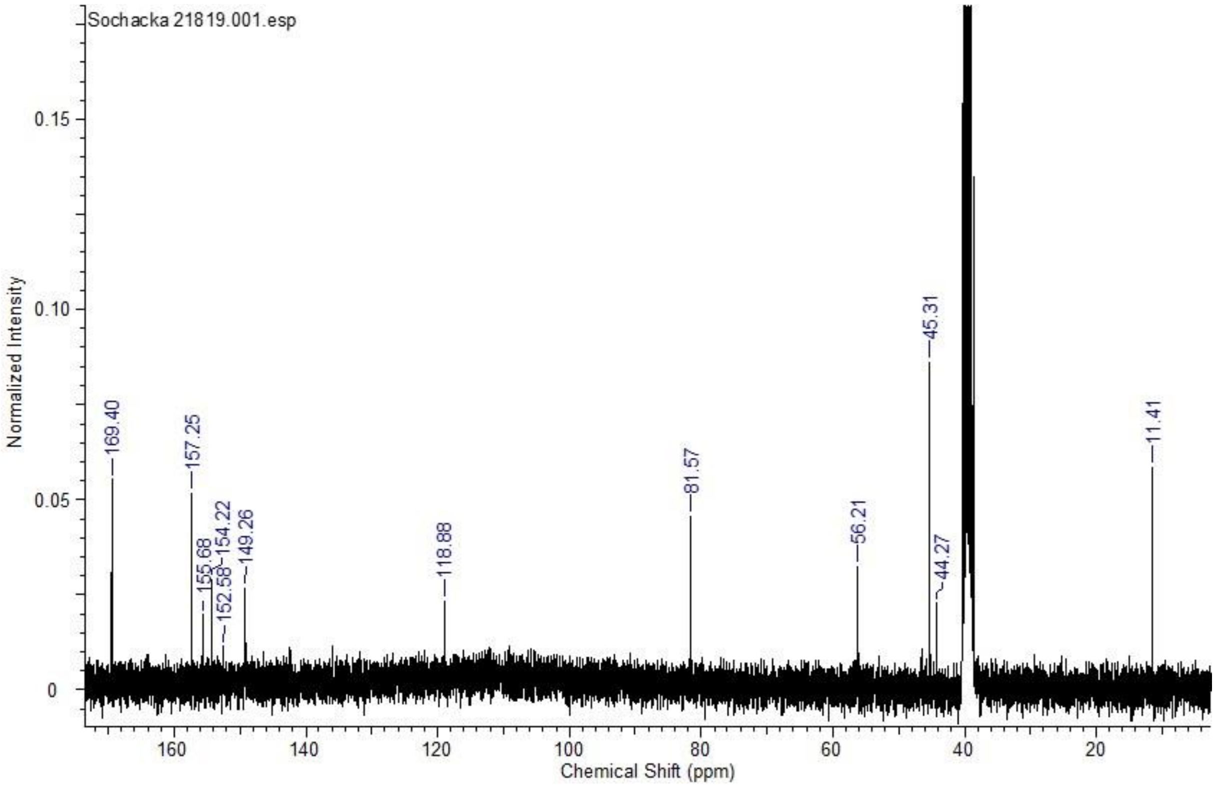

Figure S31. ^{13}C-NMR spectrum of the compound **3f** in DMSO-d_6.

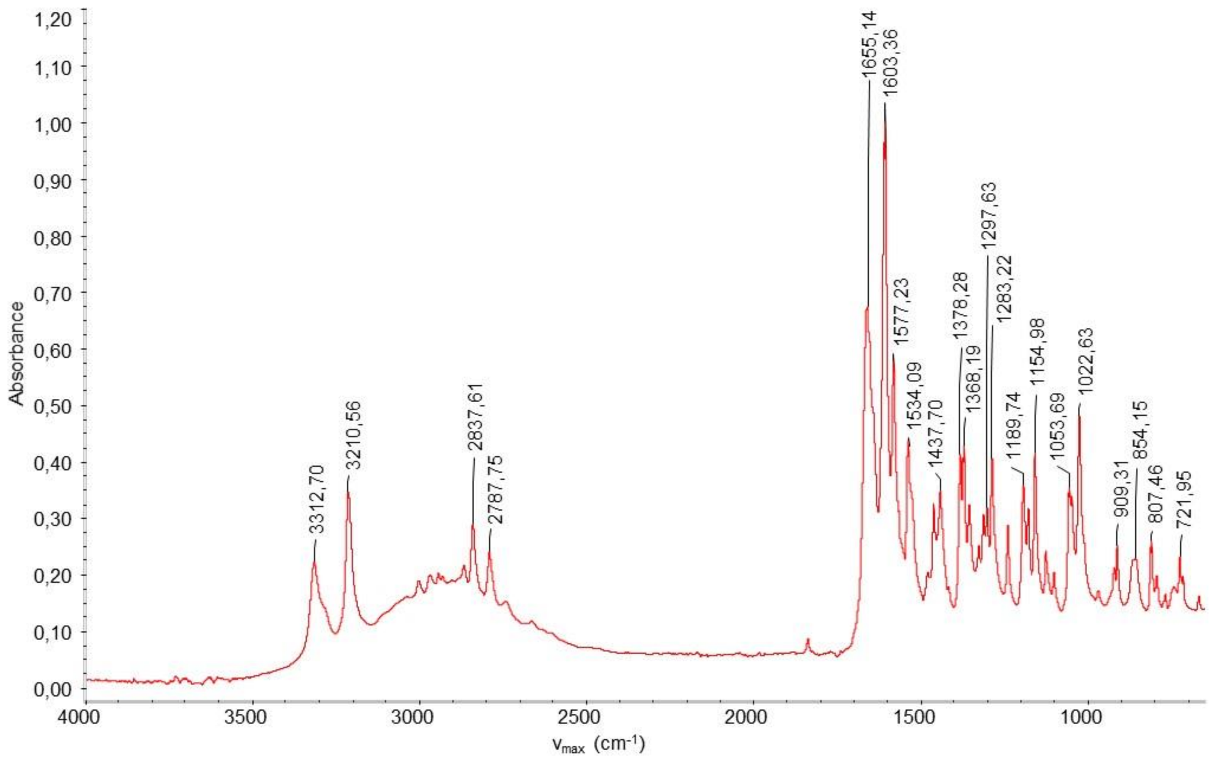

Figure S32. ATR-FTIR spectrum of the compound **3f**.

Visualizations of Nuclear magnetic resonance (NMR) and Infrared (IR) spectra of the compound 3g

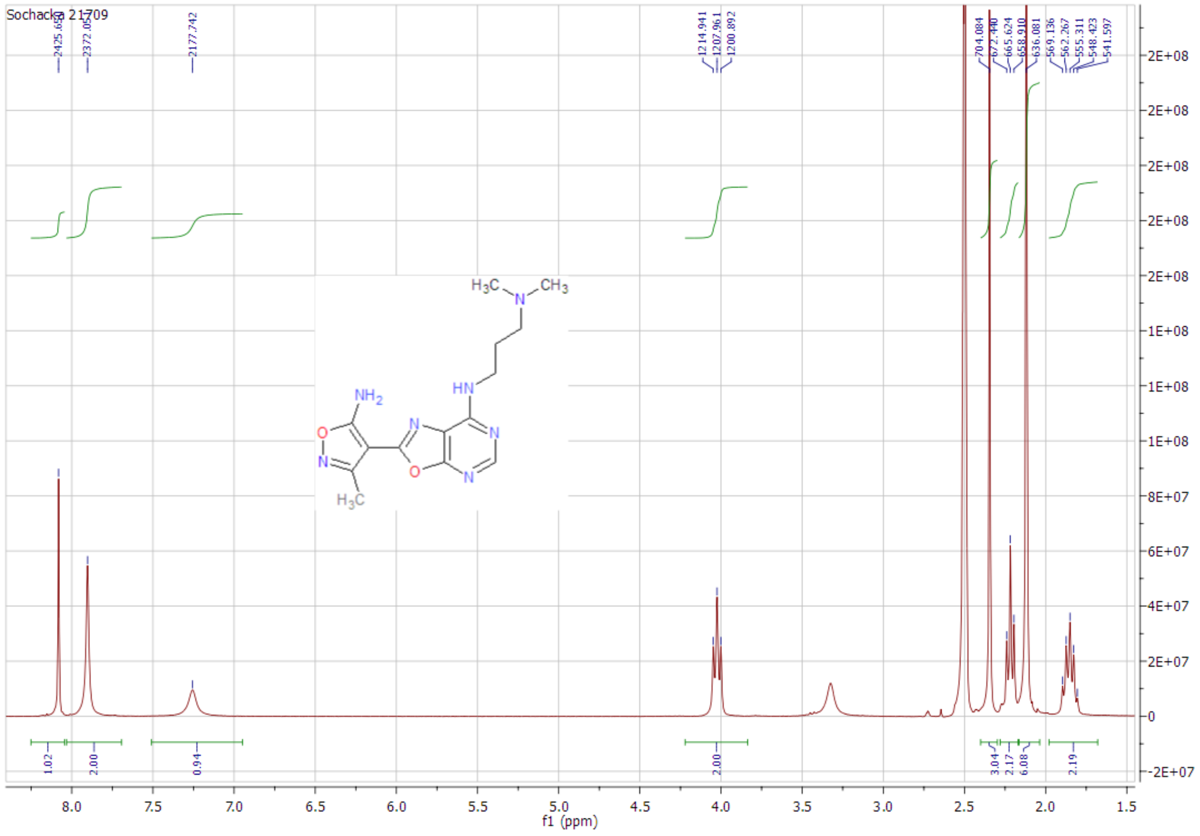

Figure S33. ^{1}H-NMR spectrum of the compound **3g** in DMSO-d_6.

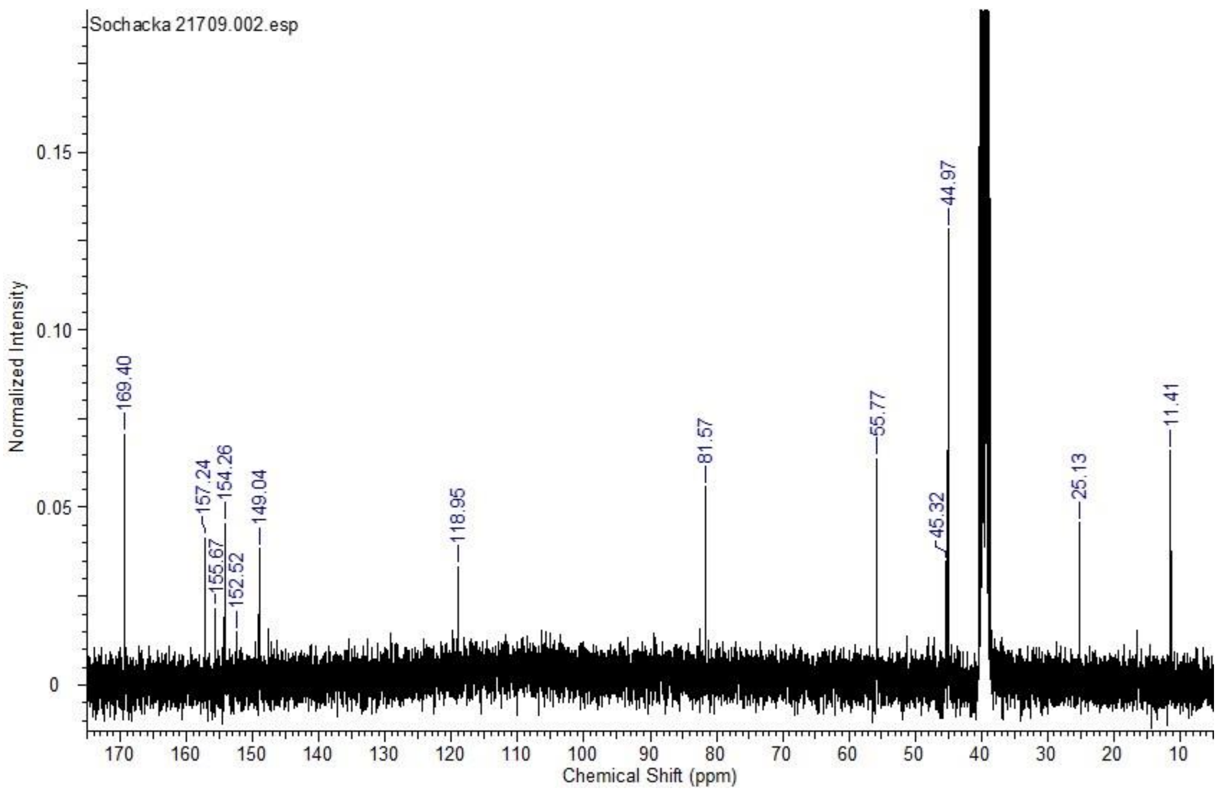

Figure S34. ^{13}C-NMR spectrum of the compound **3g** in DMSO-d_6.

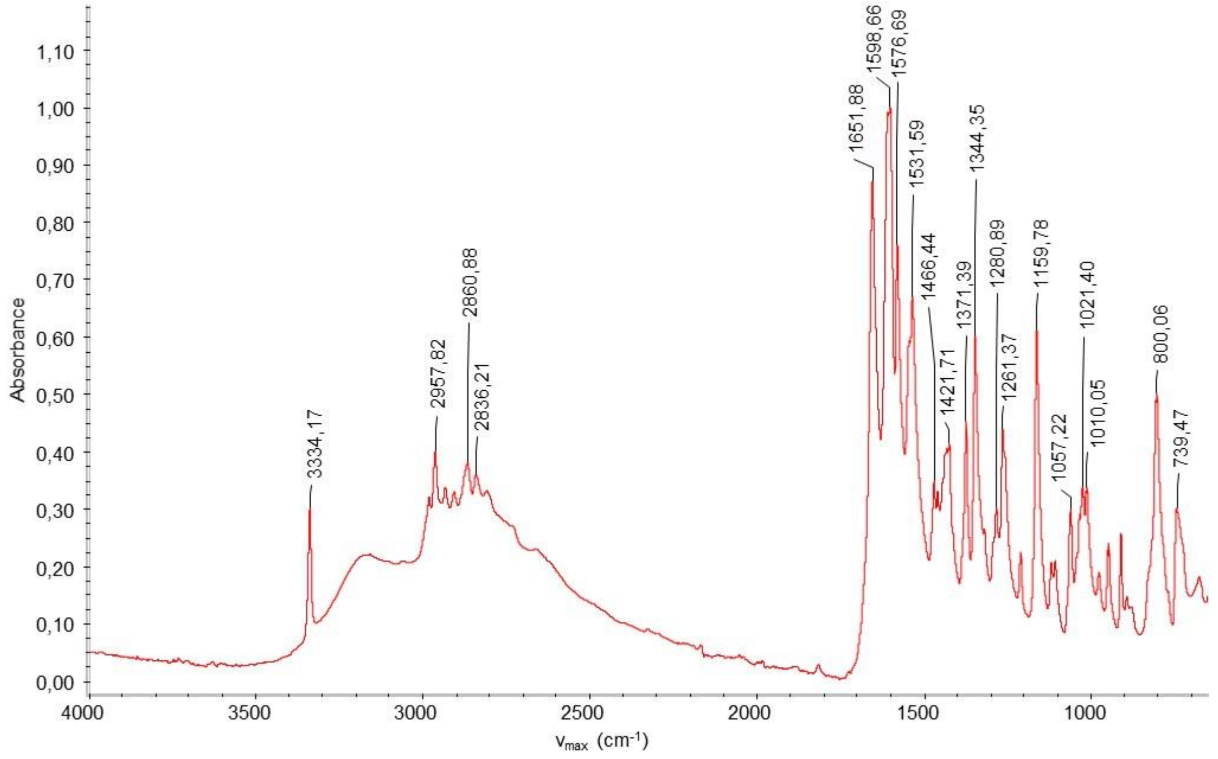

Figure S35. ATR-FTIR spectrum of the compound **3g**.

Visualizations of Nuclear magnetic resonance (NMR) and Infrared (IR) spectra of the compound 3h

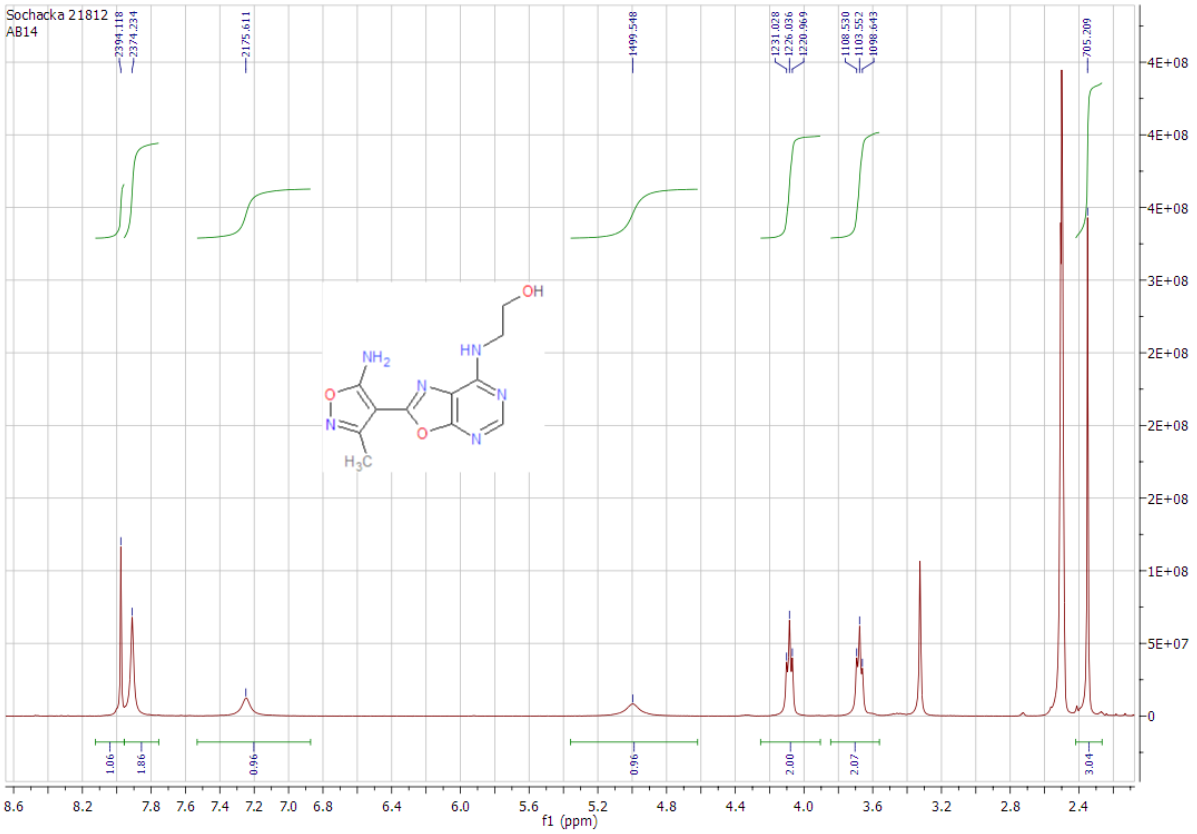

Figure S36. ^{1}H-NMR spectrum of the compound **3h** in DMSO-d_6.

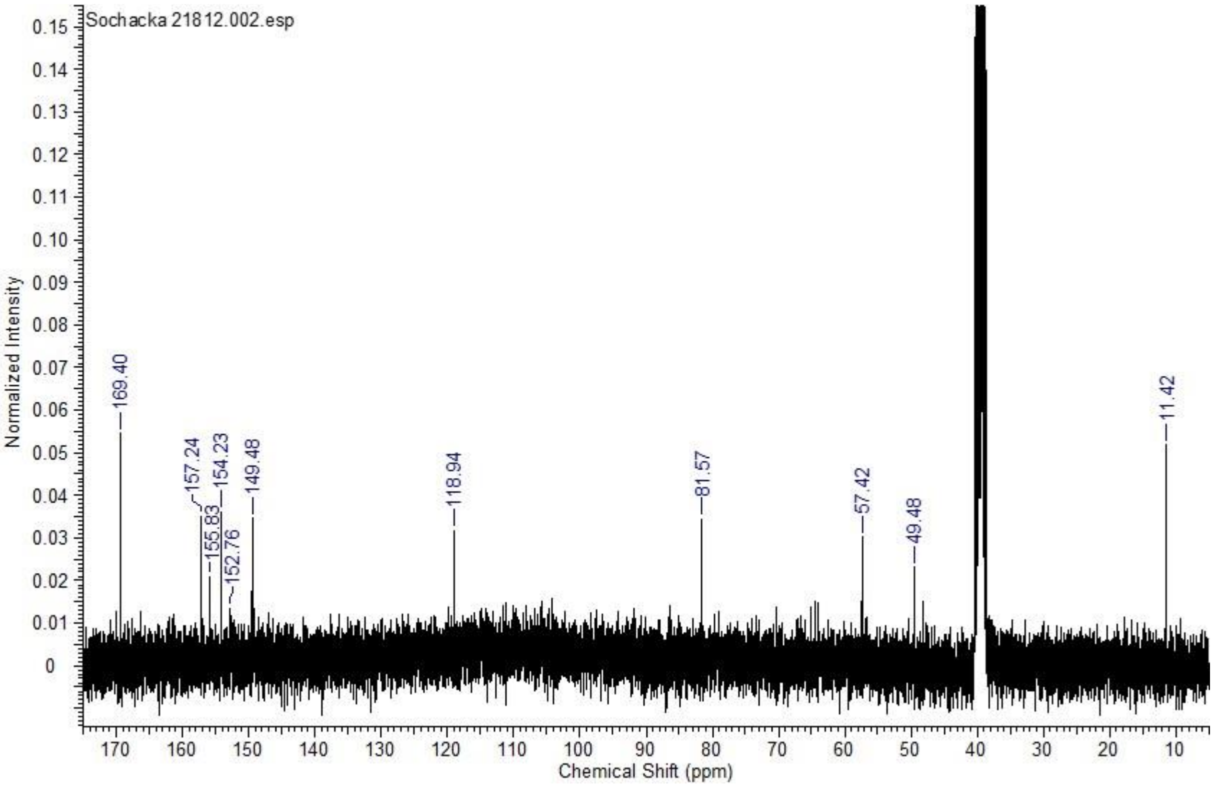

Figure S37. ^{13}C-NMR spectrum of the compound **3h** in DMSO-d_6.

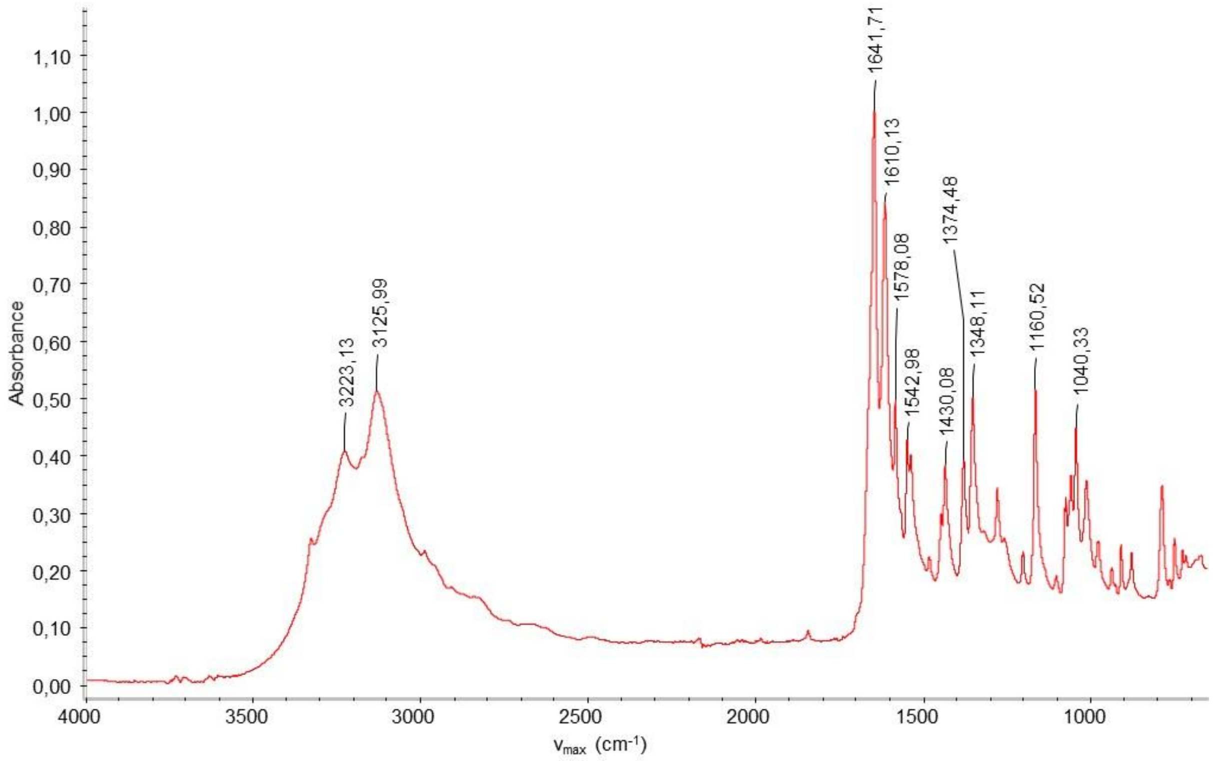

Figure S38. ATR-FTIR spectrum of the compound **3h**.

Visualizations of Nuclear magnetic resonance (NMR) and Infrared (IR) spectra of the compound 3i

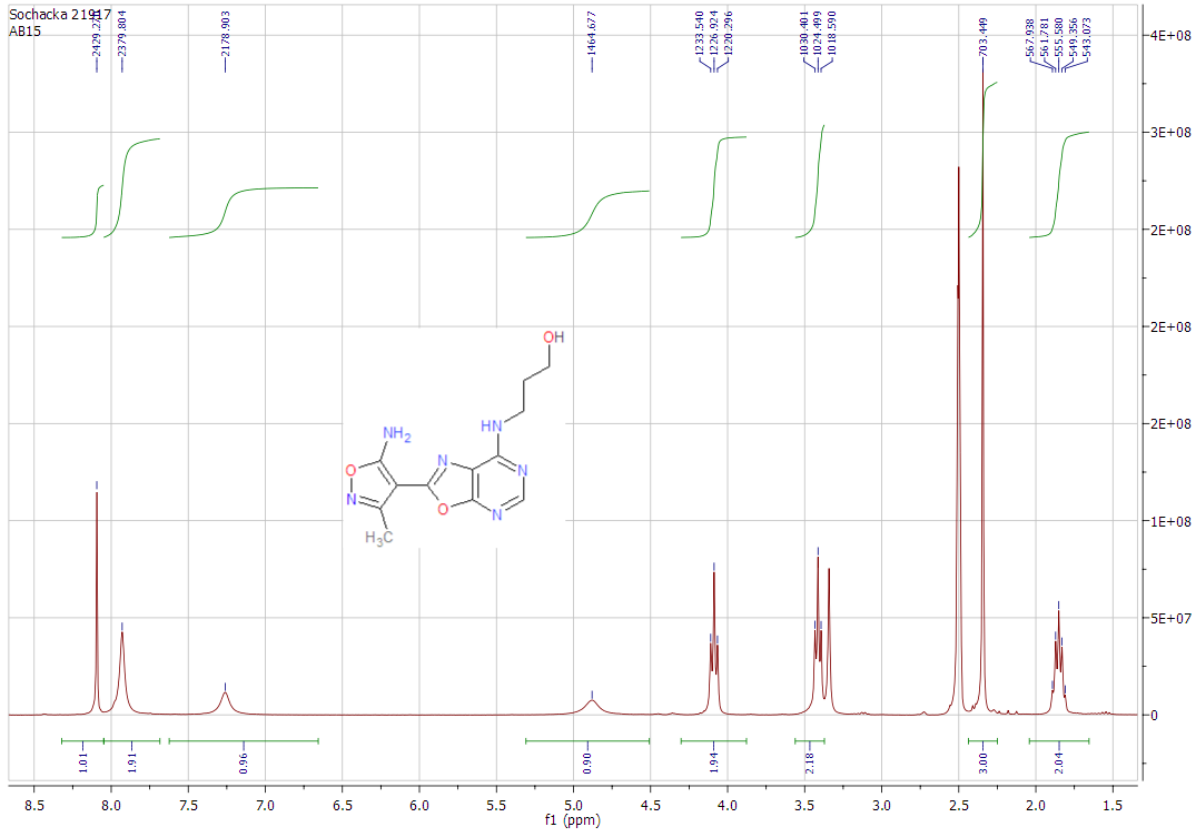

Figure S39. ^{1}H-NMR spectrum of the compound **3i** in DMSO-d_6.

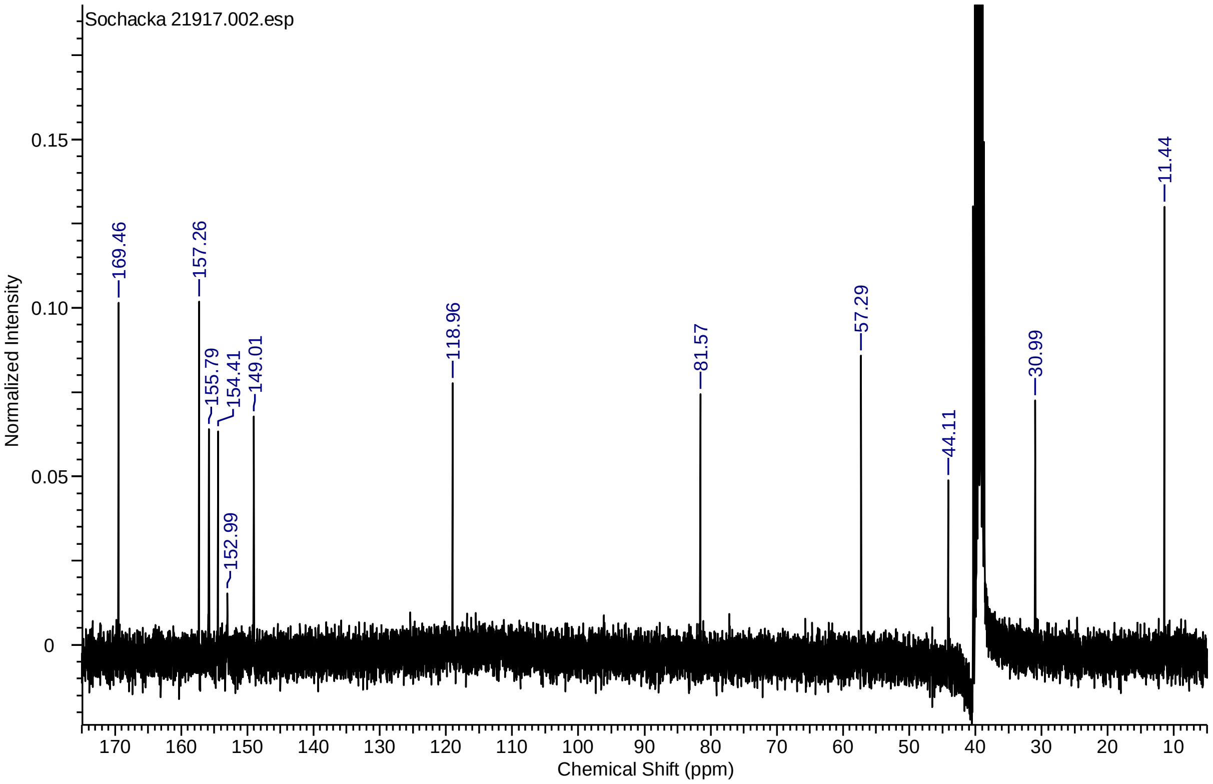

Figure S40. ^{13}C-NMR spectrum of the compound **3i** in DMSO-d_6.

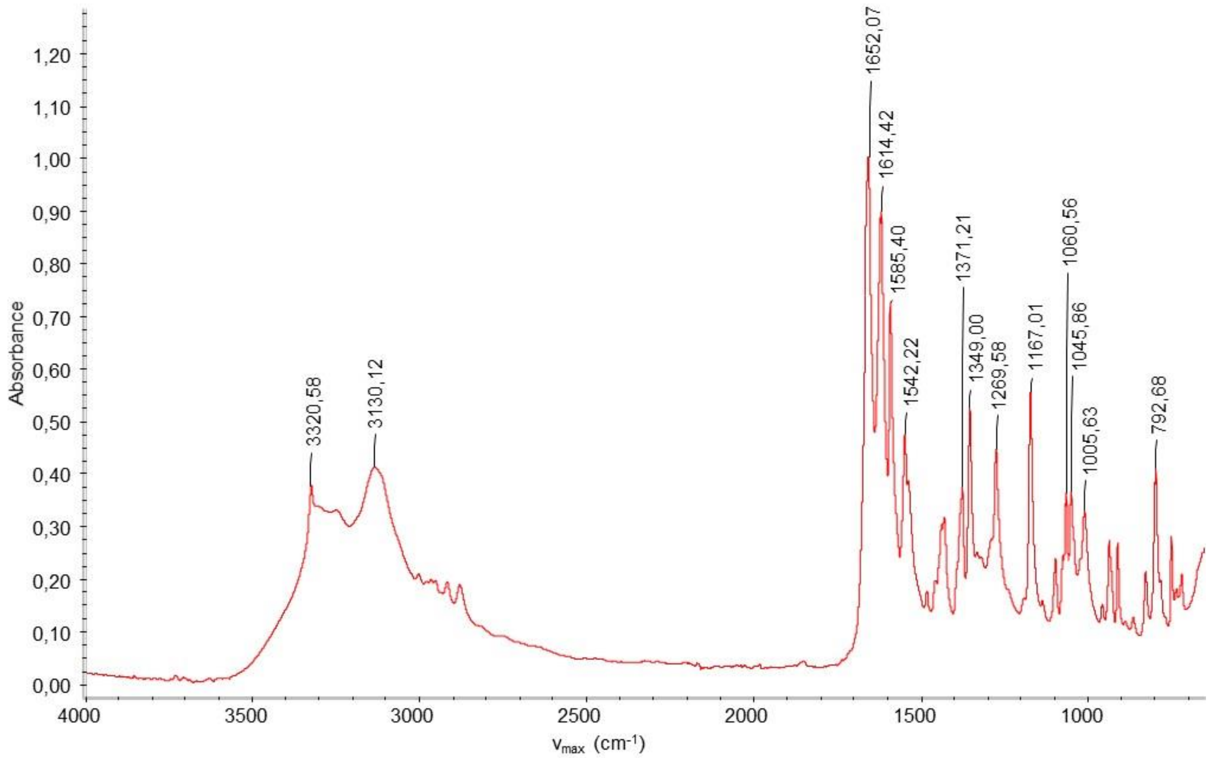

Figure S41. ATR-FTIR spectrum of the compound **3i**.

Visualizations of Nuclear magnetic resonance (NMR) and Infrared (IR) spectra of the compound 3j

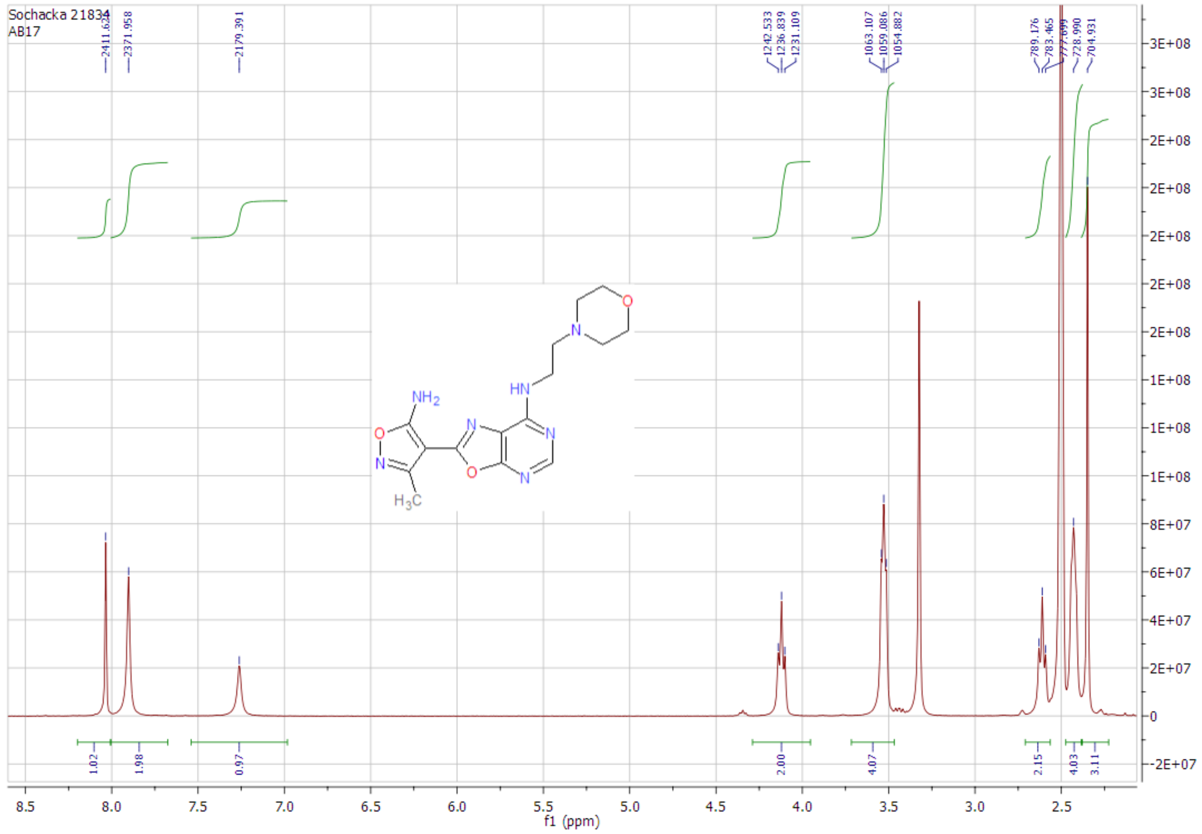

Figure S42. ^{1}H-NMR spectrum of the compound **3j** in DMSO-d_6.

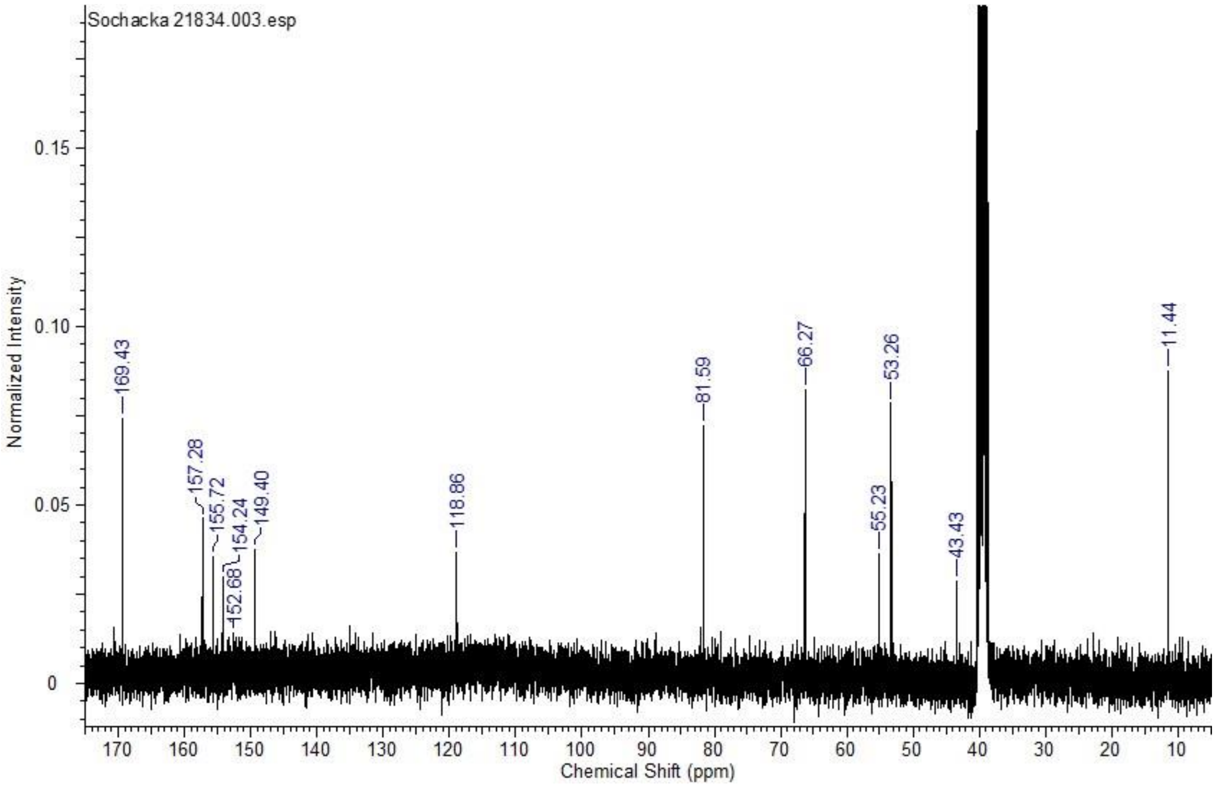

Figure S43. ^{13}C-NMR spectrum of the compound **3j** in DMSO-d_6.

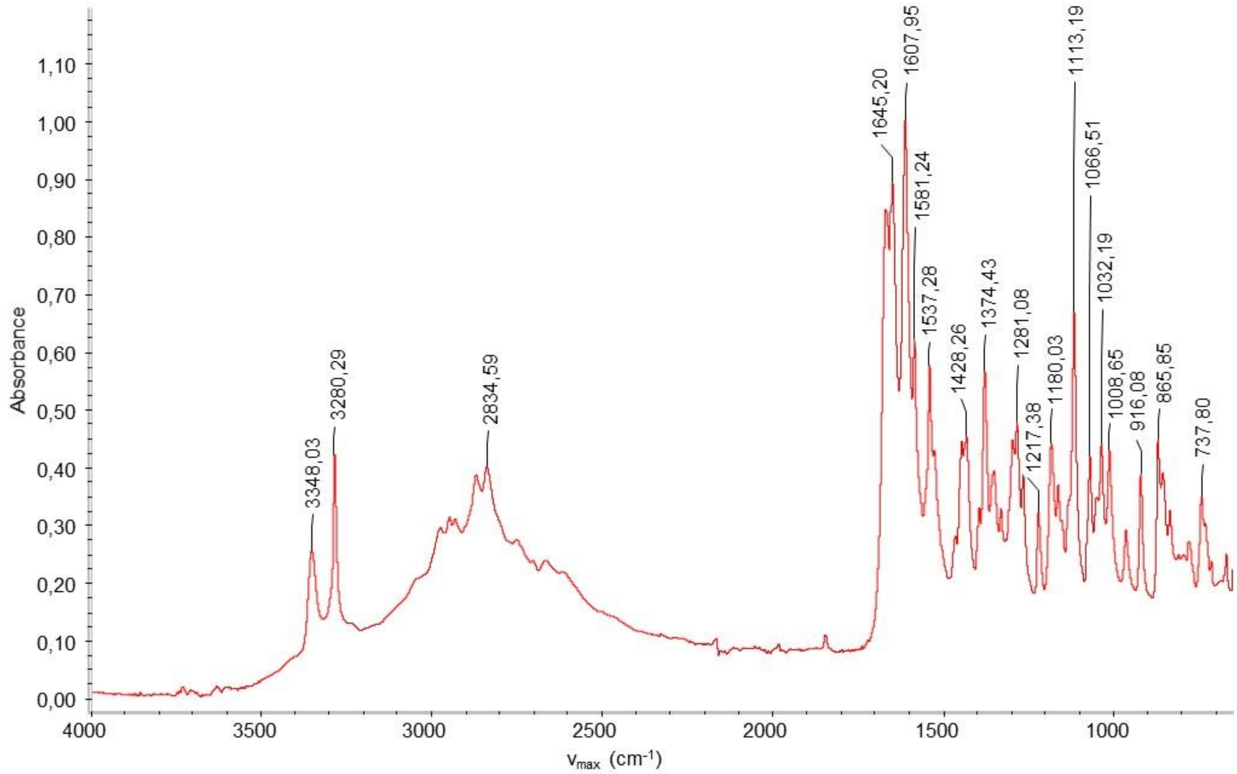

Figure S44. ATR-FTIR spectrum of the compound **3j**.

BIOLOGY

Table S14. P-glycoprotein inhibitory ability of compounds **3a**, **3e-f** and **3j**.

Compound	Concetration [µM]									
	1		2		5		10		20	
	E/E_0									
	mean	SD	mean	SD	mean	SD	mean	SD	mean	SD
**3a**	0.99	0.02	0.99	0.04	0.99	0.02	1.00	0.03	1.00	0.03
**3e**	1.12	0.11	1.19	0.07	1.21	0.07	1.24	0.05	1.21	0.09
**3f**	1.13	0.09	1.17	0.08	1.20	0.10	1.22	0.03	1.24	0.05
**3g**	1.15	0.07	1.23	0.07	1.25	0.04	1.25	0.09	1.24	0.11
**3j**	1.09	0.02	1.10	0.02	1.20	0.04	1.23	0.02	1.26	0.06
**Cisplatin**	1.02	0.02	1.04	0.03	1.05	0.02	1.05	0.02	1.07	0.02
**5-FU**	1.04	0.02	1.06	0.03	1.11	0.02	1.10	0.03	1.09	0.03

Table S15. Pro-apoptotic activity of compounds **3a**, **3e-f** and **3j**.

**Compound**	**Concentration [µM]**	**mean**	**apoptosis**		**late apoptosis**		**necrosis**	
		**life**	**mean**	**SD**	**mean**	**SD**	**mean**	**SD**
**Control**	**-**	94.78	3.91	0.56	1.16	0.74	0.15	0.18
**3a**	**1**	77.51	6.89	1.21	11.16	1.97	4.43	0.70
	**2**	74.42	5.35	7.37	11.15	3.30	9.07	4.07
	**5**	76.34	9.38	1.22	11.51	1.50	2.76	0.87
	**10**	71.40	10.99	1.91	12.07	2.43	5.55	2.06
**3e**	**1**	78.17	6.20	1.39	11.11	0.81	4.53	1.29
	**2**	73.80	4.33	1.88	12.44	0.92	9.43	1.22
	**5**	66.07	5.40	2.21	18.31	0.79	10.22	1.71
	**10**	52.63	4.05	2.24	22.59	1.26	20.74	5.00
**3g**	**1**	76.90	3.74	2.16	8.08	0.36	11.28	1.63
	**2**	74.65	9.72	11.22	9.30	2.90	6.34	5.44
	**5**	23.81	51.54	3.15	23.48	2.25	1.18	1.67
	**10**	24.62	45.24	15.02	29.23	14.73	0.90	22.04
**3f**	**1**	89.62	7.07	3.36	3.15	2.06	0.16	1.13
	**2**	83.82	3.08	7.55	7.34	1.44	5.76	3.77
	**5**	74.77	6.76	1.87	12.74	2.70	5.73	0.91
	**10**	72.06	18.64	19.26	6.51	19.20	2.78	5.22
**3j**	**1**	57.30	5.45	2.71	29.25	1.02	8.01	3.18
	**2**	61.47	8.18	7.55	26.44	0.71	3.92	6.70
	**5**	57.69	8.28	5.60	30.59	7.48	3.45	10.76
	**10**	52.76	1.34	2.93	13.55	0.42	32.35	0.63
**Cisplatin**	**1**	50.30	4.45	2.16	26.25	0.79	7.81	0.23
	**2**	52.47	7.18	7.55	24.44	2.70	4.42	1.67
	**5**	59.69	6.28	3.15	29.59	1.63	3.95	0.36
	**10**	48.76	2.34	15.02	16.55	2.90	32.35	1.63
**5-FU**	**1**	59.77	7.92	2.24	31.72	1.63	10.48	5.00
	**2**	63.94	10.65	11.22	28.91	2.90	8.86	5.44
	**5**	60.16	10.75	7.55	33.06	2.06	8.39	1.13
	**10**	55.23	3.81	19.26	16.02	1.63	34.82	14.73

Table S16. Effect on cell migration of compounds **3a**, **3e-f** and **3j**.

Compound	Concetration [µM]							
	10		25		50		100	
	mean	SD	mean	SD	mean	SD	mean	SD
**3g**	180844	11569.6	218581.6	4012.6	245687.8	8957.5	314566.8	15735.5
**Cisplatin**	171562.8	8085.4	209621	13992.6	216006.4	6219.8	253653.8	10770.3
**5-FU**	155098.248	13405.5	192391.405	11490.6	198312.887	6719.7	282974.4	8580.6
**Control**			mean			SD		
			155558.2			35476.2		
**Control T_0**			mean			SD		
			544366.45			28332.4		

MOLECULAR DOCKING

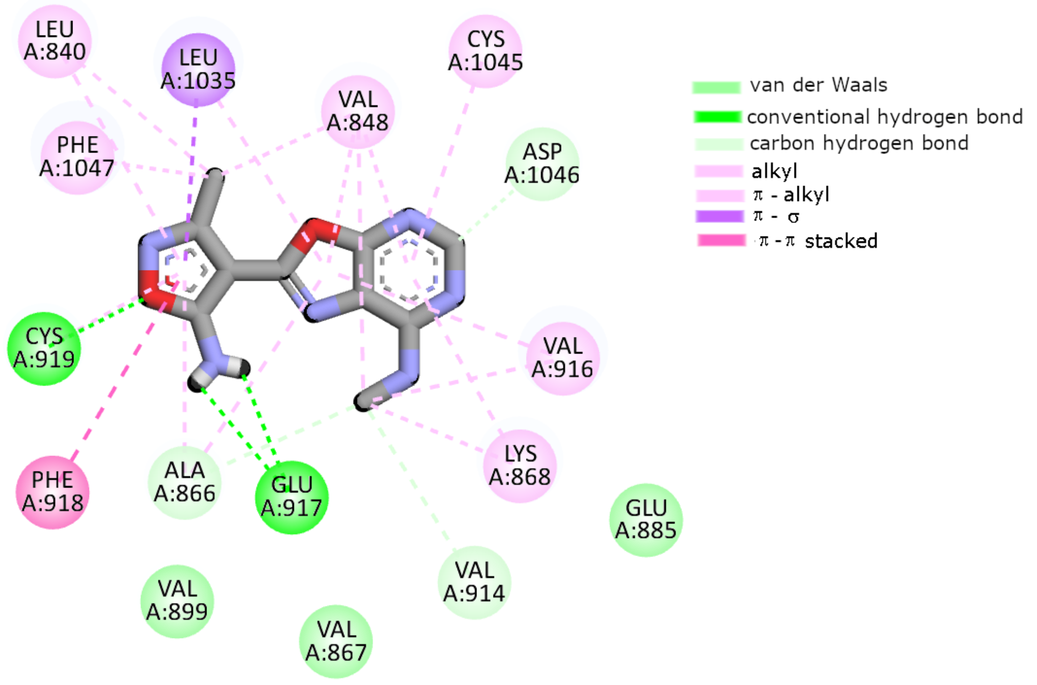

Figure S45. The possible intermolecular interactions the active site of VEGFR2 (2D representation) with **3a**.

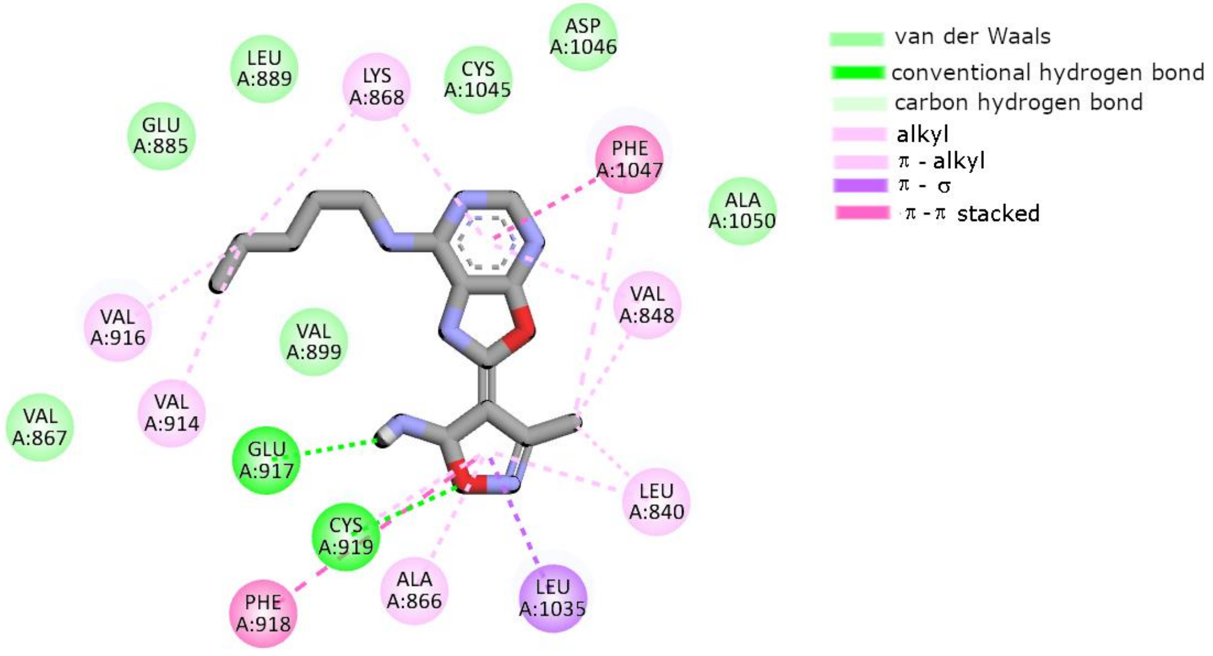

Figure S46. The possible intermolecular interactions the active site of VEGFR2 (2D representation) with **3e**.

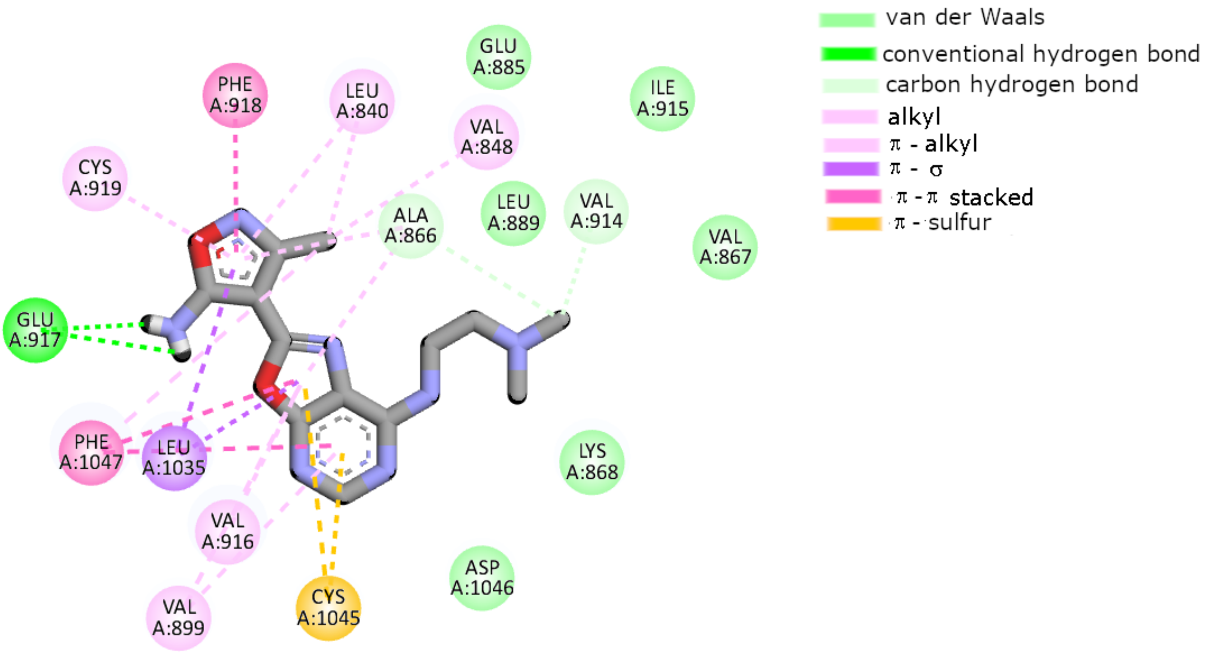

Figure S47. The possible intermolecular interactions the active site of VEGFR2 (2D representation) with **3f**.

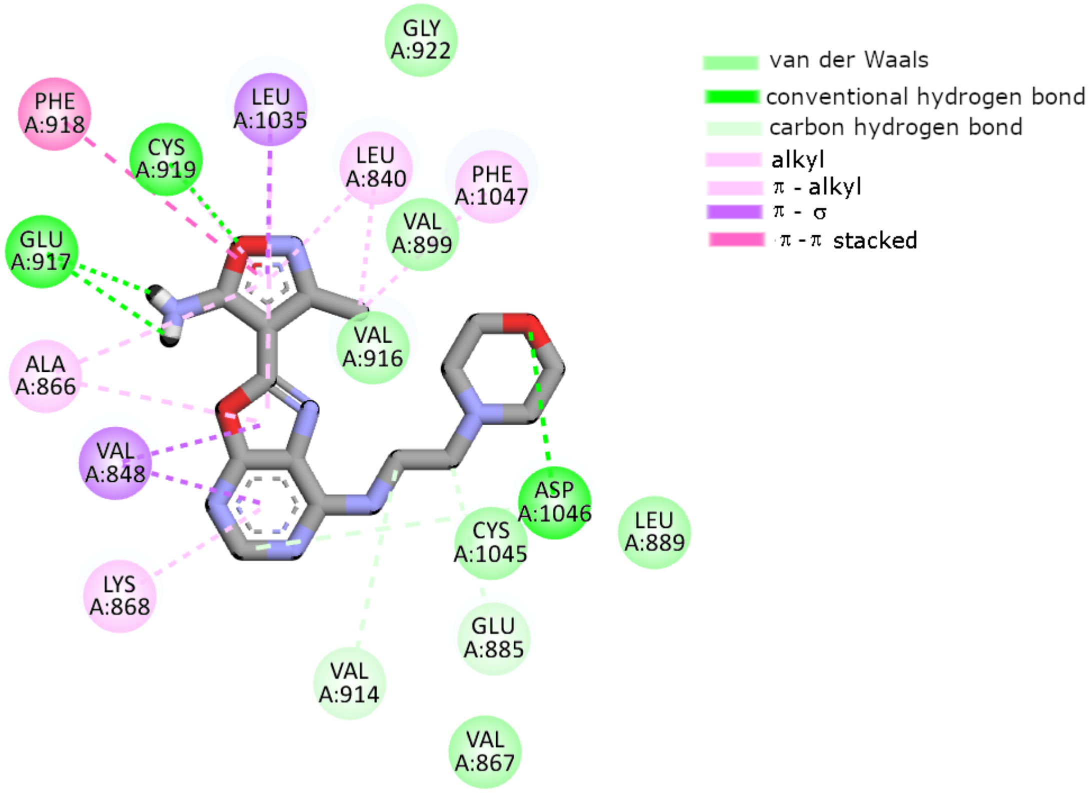

Figure S48. The possible intermolecular interactions the active site of VEGFR2 (2D representation) with **3j**.

PHYSICOCHEMICAL PROPERTIES, PHARMACOKINETICS AND ADME ACTIVITY

Table S17. Physicochemical properties of compounds **3a**, **3e-f** and **3j**.

Parameter	Compound				
	3a	3e	3f	3g	3j
MW (molecular weight) Optimal 100-600	239.03	304.16	303.14	317.16	345.13
nHA (number of hydrogen bond acceptors) Optimal 0 -12	8	8	9	9	10
nHD (number of hydrogen bond donors) Optimal 0-7	3	4	3	3	3
TPSA (topological polar surface area) Optimal 0-140	119.85	115.23	123.09	123.9	132.32
nRot (number of rotatable bonds) Optimal 0-11	1	6	4	5	4
nRing (number of rings) Optimal 0-6	3	3	3	3	4
nHet (number of heteroatoms) Optimal 1-15	8	8	9	9	10
logP (Log of the octanol/water partition coefficient) Optimal 0-3	-2.23	0.37	-0.47	-0.09	-0.30
logD (logP at physiological pH) Optimal 1-3	-0.451	2.52	0.43	0.46	0.94

Table S18. Medicinal chemistry of compounds **3a**, **3e-f** and **3j**.

Parameter	Compound				
	3a	3e	3f	3g	3j
QED (measure of drug-likenes based on the concept of desirability; attractive > 0.67, unattractive 0.49-0.67, too complex < 0.34)	0.54	0.61	0.64	0.60	0.61

SAscore (synthetic accessibility score is designed to estimate ease of synthesis of drug-like molecules; ≥ 6 – difficult, < 6 -easy to synthesize	5.801	4.43	4.05	3.96	3.81
Fsp3 (number of sp3 hybridized carbons / total carbon count, correlating with melting point and solubility; ≥0.42 is considered a suitable value)	0.0	0.5	0.38	0.43	0.46
Lipinski Rule (MW ≤ 500; logP ≤ 5; Hacc ≤ 10; Hdon ≤ 5; if two properties are out of range, a poor absorption or permeability is possible, one is acceptable)	accepted	accepted	accepted	accepted	accepted
Pfizer Rule (compounds with a high log P (>3) and low TPSA (<75) are likely to be toxic)	accepted	accepted	accepted	accepted	accepted
GSK Rule (MW ≤ 400; logP ≤ 4; compounds satisfying the GSK rule may have a more favorable ADMET profile)	accepted	accepted	accepted	accepted	accepted

Table S19. Absorption and distribution of compounds **3a**, **3e-f** and **3j**.

Parameter	Compound				
	3a	3e	3f	3g	3j
HIA (Human Intestinal Absorption; Category 1: HIA+(HIA < 30%); Category 0: HIA-(HIA < 30%); The output value is the probability of being HIA+)	0.016	0.399	0.019	0.019	0.026
F 20% (20% Bioavailability; Category 1: F20%+ (bioavailability < 20%); Category 0: F20%- (bioavailability ≥ 20%); The output value is the probability of being F20% +)	0.019	0.004	0.007	0.007	0.096

F 30% (30% Bioavailability; Category 1: F30%+ (bioavailability < 30%); Category 0: F30%- (bioavailability ≥ 30%); The output value is the probability of being F30% +)	0.011	0.548	0.002	0.002	0.044
PPB (Plasma Protein Binding; Optimal < 90%. Drugs with high protein-bound may have a low therapeutic index)	93.67%	61,04%	77.59%	75.57%	84.10%
VD (Volume Distribution; Optimal 0.04-20L/kg)	0.19	1.3	1.8	1.71	1.45
BBB Penetration (Blood-Brain Barrier Penetration Category; 1: BBB+; Category 0: BBB-; The output value is the probability of being BBB+)	0.09	0.47	0.49	0.441	0.398

Table S20. Metabolism and excretion of compounds **3a**, **3e-f** and **3j**.

Parameter	Compound				
	3a	3e	3f	3g	3j
CYP1A2 inhibitor	NO	YES	YES	YES	YES
CYP2C19 inhibitor	NO	NO	NO	NO	NO
CYP2C9 inhibitor	NO	NO	NO	NO	NO
CYP2D6 inhibitor	NO	NO	NO	NO	NO
CYP3A4 inhibitor	NO	YES	YES	YES	YES
CL Clearance (High: >15 mL/min/kg; moderate: 5-15 mL/min/kg; low)	2.05	2.19	6.543	6.618	6.87
T 1/2Category (1: long half-life; Category 0: short half-life; long half-life: >3h; short half-life: <3h)	0.84	0.723	0.77	0.742	0.557

Table S21. Calculated LogP (Decimal logarithm of the n-octanol/water partition coefficient) of the compounds series **3a-j**.

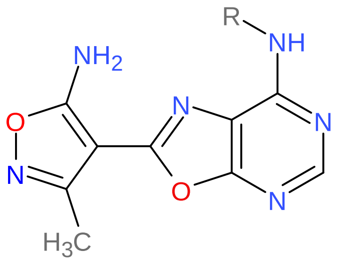

the compound series **3a-j**

Compound	Calculated LogP				
	**Method1** with ALOGPS 2.1 software	**Method2** with MarvinSketch (ChemAxon software)	**Method3** ChemSketch (ACDLabs software)	**Method4** with BIOVIA Draw 2019 software	**Method5**[a] with ADMETlab **LogP/LogD**
**3a, R=Me**	1.02	0.02	1.14±1.41	0.1635	1.205/1.284
**3b, R=Et**	1.64	0.37	1.67±1.41	0.5123	1.595/1.467
**3c, R=n-Pr**	1.91	0.83	2.20±1.41	1.036	1.985/1.781
**3d, R=n-Bu**	2.33	1.23	2.73±1.41	1.4922	2.375/1.936
**3e, R=n-pentyl)**	2.74	1.63	3.26±1.41	1.9484	2.766/2.382
**3f, R=Me_2NEt**	1.02	0.00	0.98±1.41	0.3022	1.137/1.379
**3g, R=Me_2NPr**	**1.35**	**0.05**	**1.32±1.41**	**0.3646**	**1.527/1.556**

**3h, R=HOEt**	0.37	-0.72	0.36±1.43	-0.3759	0.568/0.211
**3i, R=HOPr**	0.65	-0.67	0.11±1.43	-0.3135	0.958/1.093
**3j, R=2-(morpholin-4-yl)ethyl)**	**0.73**	**-0.34**	**0.59±1.42**	**-0.0101**	**0.908/1.3**

[a] **Method5 -** Wildman-Crippen LogP value. S. A. Wildman and G. M. Crippen, J CHEM INF COMP SCI, 1999, 39(5): 868-873; For LogD – “Methods and principles in medicinal chemistry “, 18 (pp. 21–45). Weinheim: Wiley-VCH.
